# Nonmonotonic Screening and Solvation Dynamics of the Electrical Double Layer in Concentrated Lithium Salt Electrolytes

**DOI:** 10.1038/s41467-026-75999-2

**Published:** 2026-07-29

**Authors:** Xiaoting Yin, Feng Wang, Zengming Zhang, Ru-Yu Zhou, Zhaobin Chen, Tairui Wu, Li Zhang, Jun Huang, Deyin Wu, Jun Cheng, Bingwei Mao, Jiawei Yan

**Affiliations:** 1 State Key Laboratory of Physical Chemistry of Solid Surfaces, College of Chemistry and Chemical Engineering, Xiamen University, Xiamen, 361005 China; 2 Laboratory of AI for Electrochemistry (AI4EC), IKKEM, Xiamen, 361005 China; 3 Institute of Energy and Climate Research, IET-3: Theory and Computation of Energy Materials, Forschungszentrum Jülich GmbH, 52425 Jülich, Germany; 4 Theory of Electrocatalytic Interfaces, Faculty of Georesources and Materials Engineering, RWTH Aachen University, Aachen, 52062 Germany

**Keywords:** Batteries, Computational chemistry, Characterization and analytical techniques

## Abstract

Understanding the electrical double layer in lithium-ion battery electrolytes is fundamental to improving interfacial processes that govern battery performance and lifetime. However, the microstructure and dynamics of the electrical double layer in highly concentrated lithium salt solutions remain elusive. Herein, combining electrochemical analyses, in situ gap-enhanced Raman spectroscopy and molecular dynamics simulations, we reveal nonmonotonic variation of electrostatic screening with concentration and different exchange kinetics of anions and solvent molecules in the solvation sheath of lithium ions. We find that increasing solvation entropy favors the formation of an anion-rich solvation environment in highly concentrated electrolytes, accelerating Li^+^–anion exchange relative to Li^+^–solvent exchange. This dynamic coordination behavior enables Li^+^ to reorganize its solvation structure more readily during intercalation and deintercalation, thereby improving the kinetics and reversibility of these processes. Overall, this work provides thermodynamic and kinetic insights for the electrolyte design of advanced lithium-ion battery systems.

## Abstract (web summary)

The electrical double layer in concentrated lithium electrolytes remains unclear. This study shows nonmonotonic screening and entropy-driven anion-rich solvation that accelerates Li^+^ exchange kinetics and improves Li^+^ interfacial transport.

## Introduction

All electrochemical energy conversion and storage systems rely on electrolytes to maintain charge separation and enable ion transport, thereby sustaining electrochemical reactions and device operation^1–3^. At the electrode/electrolyte interface, the electrical double layer (EDL) regulates the local electrostatic and chemical environment in which interfacial reactions occur, including the electrolyte reduction reactions that ultimately govern the formation and composition of the solid electrolyte interphase (SEI) in lithium-ion batteries (LIBs)^4^. However, our knowledge of EDL in the concentration regime of practical interest is very limited. The relationship between the EDL, ion solvation structures, and electrochemical performance in concentrated LIBs electrolytes remains unclear. Early descriptions of the EDL evolved into the classical Gouy–Chapman–Stern (GCS) model, which treats the interface as a compact Helmholtz layer in series with a diffuse layer of thermally distributed ions^5^. The GCS framework provides a convenient continuum description of interfacial screening but fundamentally relies on the Debye–Hückel theory, which assumes point-like ions, weak electrostatic correlations, and dilute-solution behavior^6^. The Debye-Hückel theory provides a first-order description of ion-ion interactions in dilute aqueous solutions, but it fails at high concentrations where short-range correlations play important roles in electrolyte dissociation and ion clustering^7–9^. As electrolyte concentration increases beyond the dilute regime assumed by the Debye–Hückel and GCS frameworks, short-range ion-ion and ion-solvent interactions begin to dominate the interfacial structure. In this regime, complex competitive coordination among anions, solvents, and cations gives rise to three distinct cation solvation structures: solvent-separated ion pairs (SSIPs), contact ion pairs (CIPs), and aggregates (AGGs)^10,11^. These coordination states reflect not only local electrostatic correlations but also the entropic driving forces that emerge in crowded solvation environments^12–14^. Viewing the EDL through this combined framework of interfacial structure and entropic effects yields a more continuous and physically grounded description of screening, thereby overcoming key limitations of the classical GCS model in practical lithium-ion battery electrolytes.

Within the EDL of lithium-ion batteries, the interfacial solvation environment governs Li^+^ desolvation, a key step prior to its intercalation into the host material^15^. Importantly, this desolvation process is intrinsically coupled to the reorganization of interfacial electric fields, the evolution of local solvation structures, and the development of ion-concentration gradients. Under realistic electrolyte conditions, these interconnected processes occur simultaneously, which makes it challenging to resolve their dynamics in a synchronous manner^16,17^. Consequently, most existing studies capture only one aspect of this coupled process, such as measuring the electrochemical response or characterizing bulk solvation structure, and therefore cannot capture the nanoscale interfacial restructuring that ultimately determines how Li^+^ sheds its solvation sheath at the electrode surface. Among various lithium salts, lithium bis(fluorosulfonyl)imide (LiFSI) is particularly effective in facilitating Li^+^ desolvation due to the relatively weak interaction between Li^+^ and the smaller FSI^**–**^ anion, which significantly enhances interfacial reaction kinetics^18^. Beyond the direct Li^+^-anion interactions, the desolvation process is intimately coupled to ion transport within the EDL. Furthermore, the salt concentration also plays a crucial role in determining ion transport mechanisms. In liquid electrolytes with varying salt concentrations, Li⁺ migration can occur via vehicular transport, structural transport, or a combination of both, with the dominant pathway closely related to the electrolyte composition and coordination structure^19,20^. These transport processes are tightly coupled with interfacial solvation-desolvation dynamics, which directly regulate the kinetics of Li^+^ intercalation into the anode material^21,22^. However, at high electrolyte concentrations, the dynamic coupling between solvation-desolvation and intercalation kinetics remains poorly understood, despite its decisive role in governing interfacial charge transfer.

Deciphering these coupled interfacial phenomena is far from straightforward using electrochemical analyses alone, due to the inherent complexity, dynamic behavior, and nanoscale heterogeneity of electrode-electrolyte interfaces^23,24^. Thus, integrating advanced in situ characterization techniques is essential for elucidating the evolution of solvation structures within the EDL and the kinetics of interfacial Li^+^ transport. Complementary to these experimental approaches, computational simulations have primarily focused on rapidly screening combinations of solvents, salts, and additives to predict electrolyte performance^25–27^. Classical molecular dynamics (CMD) and ab initio molecular dynamics (AIMD) are commonly used to investigate the solvation structures and transport properties of electrolytes^28,29^. However, existing research has yet to fully consider the coupling between thermodynamic entropy and interfacial kinetic exchange, nor to systematically explore how variations in electrolyte concentration modulate electrochemical behavior. This oversight creates a persistent disconnect between computational predictions and experimental observations, limiting the predictive power and mechanistic understanding of interfacial phenomena. To bridge this gap, we integrate electrochemical measurements and in situ spectroscopic characterization with molecular dynamics simulations based on the Constant Potential Method (CPM). This combined approach enables a detailed description of ion-solvent coordination in electrolytes, while explicitly accounting for potential-dependent restructuring of the electrical double layer and entropy-mediated interfacial effects.

In this work, we aim at interconnecting electrical double-layer properties and ion-solvent exchange dynamics in lithium-based electrolytes. A model electrolyte system composed of lithium bis(fluorosulfonyl)imide (LiFSI) salt and tetraglyme (G4) is employed to probe both GCS-compliant and GCS-deviant interfacial regimes. By combining electrochemical atomic force microscopy (EC-AFM) with in situ gap-enhanced Raman spectroscopy, we resolve the nanoscale microstructural evolution of the EDL. To quantitatively bridge interfacial ordering and molecular-level ion transport behavior, atomistic molecular dynamics simulations (including classical and machine-learning-based models) are further employed. The simulation reveals an entropy-mediated mechanism that governs the competition between Li^+^–anion and Li^+^–solvent exchange dynamics at different salt concentrations, ultimately influencing the reversibility of Li^+^ intercalation/deintercalation at the electrode. These results identify entropy as a design lever for optimizing solvation dynamics and guiding next-generation electrolyte development.

## Results

### Electrochemical analyses of interfacial behavior

Electrolyte concentration regimes can be classified according to the nature of the Li^+^-solvation sheath: in highly concentrated electrolytes, few or no free solvent molecules are present, whereas in diluted electrolytes, uncoordinated free solvent molecules exist^30^. In this paper, we study the capacitive behavior of highly oriented pyrolytic graphite (HOPG) within the ideally polarizable potential window, with particular emphasis on the differential capacitance near the potential of zero charge (PZC). For electrodes in contact with dilute electrolytes, the interfacial capacitance is commonly described by the Gouy-Chapman-Stern (GCS) model^31^. In this framework, the double-layer capacitance is modeled as two capacitors connected in series, expressed as

1 $$\frac{1}{{C}_{{{\rm{dl}}}}}=\frac{1}{{C}_{{{\rm{H}}}}}+\frac{1}{{C}_{{{\rm{GC}}}}},$$

where *C*_H_ is the inner layer or Helmholtz capacitance and *C*_GC_ is the diffuse layer capacitance, also known as the Gouy–Chapman capacitance^6^. However, it has been shown for many cases that the GCS model does not hold for highly concentrated electrolytes, where ion correlations and crowding effects become significant.

The differential capacitance was measured using real-time, non-invasive electrochemical impedance spectroscopy (EIS) at a series of electrolyte concentrations, aiming to elucidate the underlying EDL structures and assess the validity of the GCS model for our cases. The EIS data were interpreted using the equivalent circuit model shown in Supplementary Fig. 1, in which the high-frequency semicircle corresponds to bulk solution, the intermediate-frequency semicircle represents processes associated with chemisorption and double layer charging, and the low-frequency semicircle is attributed to side reactions at the interface^32–34^. The model neatly fits EIS data at different potentials for different electrolyte concentrations (Supplementary Figs. 2–6). The adsorption capacitance (*C*_ad_) and the double-layer capacitance (*C*_dl_) exhibit distinct dependencies on both electrolyte concentration and applied potential (Supplementary Fig. 7).

**Fig. 1:**
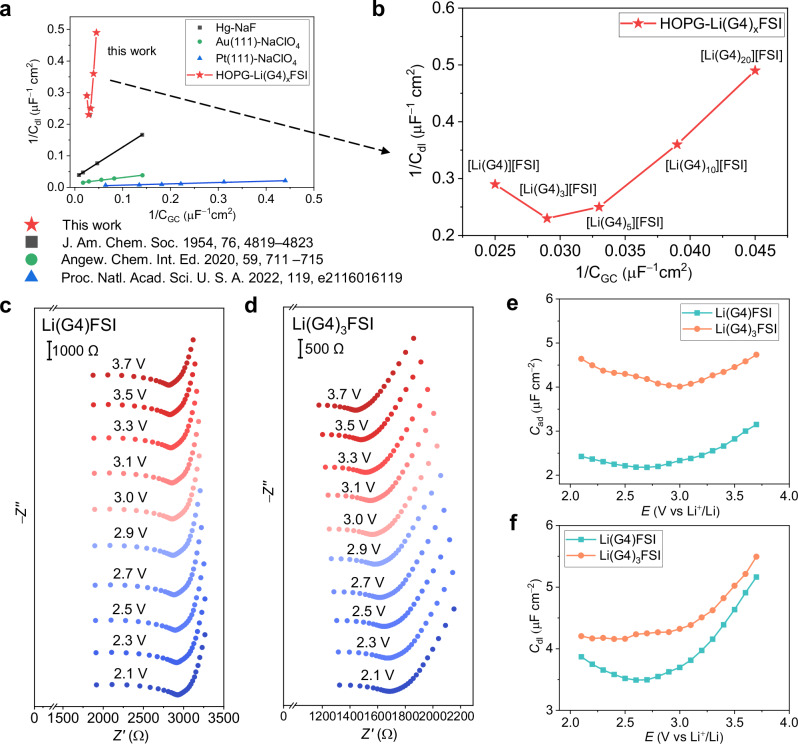
Electrochemical measurements in glyme-based electrolytes with different concentrations. **a, b** Parsons-Zobel plots comparison between the previous reported work and this work. **c, d** Nyquist plots of Li(G4)FSI (**c**) and Li(G4)_3_FSI (**d**) at different potentials. **e** *C*_ad_-*E* curves of highly oriented pyrolytic graphite (HOPG) in Li(G4)FSI and Li(G4)_3_FSI. **f** *C*_dl_-*E* curves of HOPG in Li(G4)FSI and Li(G4)_3_FSI.

We performed a Parsons–Zobel (PZ) analysis (Fig. 1a), where the measured inverse capacitance at various ionic strengths is plotted against the inverse diffuse layer capacitance predicted by Gouy–Chapman theory. A detailed derivation of the calculation of *C*_GC_ is provided in the Supporting Information. Figure 1a gives the PZ plots of our data for HOPG, together with the previous data for Pt(111)^35^, Au(111)^36^, and the classical data of Grahame for a mercury electrode^37^. Remarkably, in non-aqueous electrolytes, the inverse capacitance (1*/C*_dl_) exhibits a non-monotonic trend as salt concentration increases. Specifically, in highly concentrated [Li(G4)][FSI] systems (Fig. 1b), the double-layer capacitance decreases with electrolyte concentration, in stark contrast to the predictions of the GCS model. We refer to this as the non-GCS regime.

To better understand this anomalous behavior, we compared [Li(G4)][FSI] (1:1 molar ratio of lithium bis(fluorosulfonyl)imide to tetraglyme) with a diluted counterpart, [Li(G4)_3_][FSI] (1:3 molar ratio). Cyclic voltammetry (CV) reveals that at potentials below 2.5 V_Li_, the cathodic current density of the low-concentration electrolyte ([Li(G4)_3_][FSI]) exceeds that of the concentrated system ([Li(G4)][FSI]), indicating distinct interfacial adsorption characteristics (Supplementary Fig. 8). Simultaneously, [Li(G4)][FSI] exhibits markedly higher interfacial resistance compared to [Li(G4)_3_][FSI] (Fig. 1c and d), reflecting enhanced ion-ion interactions in the concentrated regime. The potential-dependent, area-normalized values for electrolytes with two concentrations are shown in Fig. 1e and f. In the [Li(G4)][FSI], the *C*_ad_ and the *C*_dl_ exhibit a clear potential-dependence, but it is significantly lower than that in the dilute [Li(G4)_3_][FSI] system. The smaller interfacial capacitance in the high-concentration electrolyte is likely attributed to the changes of the cation solvation structure. A detailed analysis of the cation solvation structure will be presented in a later section.

The potential of zero charge (PZC) refers to the electrode potential at which the net surface charge density becomes zero in a given electrolyte, typically corresponding to a local minimum in differential capacitance^35^. In the [Li(G4)][FSI], the PZC is located at approximately 2.8 V_Li_. However, the presence of free solvent molecules likely shifts the PZC to around 3.0 V_Li_, corresponding to the inflection point where the interfacial capacitance begins to increase significantly. When *E* > 3.0 V_Li_, the electrode surface starts to accumulate positive charge, resulting in a pronounced increase in capacitance (Fig. 1f). This interpretation is subsequently confirmed by force-distance measurements. Supplementary Fig. 9 shows the total capacitance of the interface, defined as

2 $${C}_{{{\rm{tot}}}}={C}_{{{\rm{dl}}}}+{C}_{{{\rm{ad}}}}$$

In the [Li(G4)_3_][FSI], from the PZC to positive polarization, FSI⁻ anions act as counterions for charge screening, and the potential dependence of total capacitance exhibits similar trends as in [Li(G4)][FSI]. Conversely, from the PZC to negative polarization, the interfacial capacitance is strongly influenced by solvent adsorption, exhibiting a turning point at approximately 2.5 V_Li_, suggesting a preferential adsorption of free solvent molecules onto the electrode surface.

As a summary of EIS results, we observe a pronounced deviation from classical Gouy–Chapman–Stern screening behavior in highly-concentrated solutions. Instead of decreasing monotonically with increasing salt concentration, the effective decay length of interfacial charge screening exhibits a nonmonotonic dependence on concentration. This anomalous screening likely originates from concentration-induced reorganization of the local solvation structure and ion correlations, rather than from conventional long-range electrostatic underscreening^38^. Ultrahigh-concentration electrolytes exhibit substantially increased viscosity^39^, leading to suppressed interfacial charge-storage capability, as reflected in a persistently low capacitance that is independent of potential (Supplementary Fig. 10). Under such conditions, the classical GCS model becomes inadequate due to pronounced ion-electrode interactions.

### Non-aggregated anionic structures in the positive PZ slope regime

Salt concentration is a particularly intriguing factor that merits further investigation, as it directly shapes the solvation structure of Li^+^ ions in solution and subsequently influences all other electrolyte properties^40^. To elucidate the impact of concentration variations on Li^+^ solvation structure, we employed in situ atomic force microscopy (AFM) and gap-enhanced Raman spectroscopy to probe interfacial solvation structures and their dependence on potential, particularly under conditions where the classical GCS model breaks down. Additionally, molecular dynamics simulations with the Constant Potential Method (CPM) were conducted to complement and corroborate our experimental observations.

**Fig. 2:**
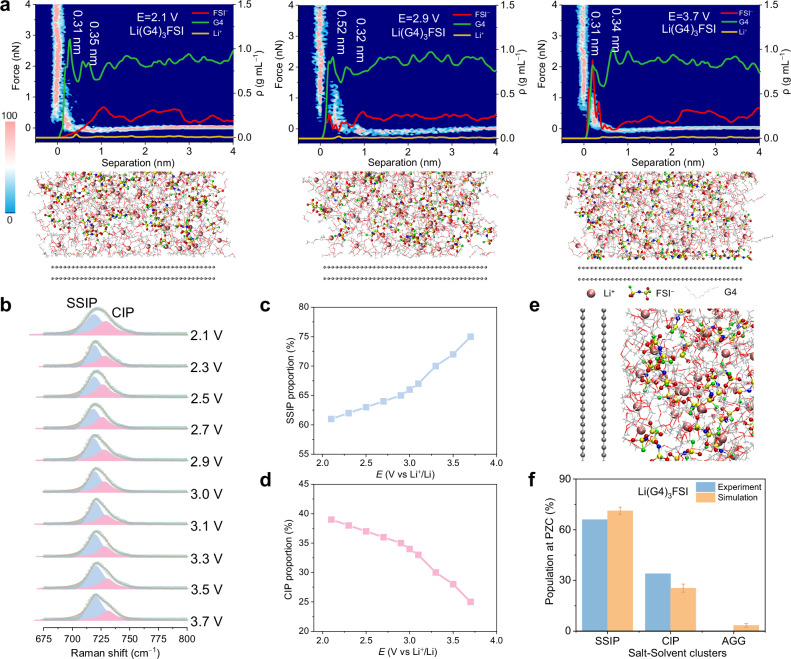
Characterization of the Li^+^ solvation structure in low-concentration [Li(G4)_3_][FSI] electrolyte. **a**, Two-dimensional atomic force microscopy force-separation histograms from 20 independent force curves for [Li(G4)_3_][FSI] obtained at potentials of *E* = 2.1 V_Li_, *E* = 2.9 V_Li_, and *E* = 3.7 V_Li_, with the probability distribution indicated by the color scale shown on the left. The corresponding ion number density profiles of FSI^–^ (red), G4 (green) and Li^+^ (yellow) obtained from MD simulations at PZC − 1.0 V, PZC, PZC + 1.0 V are shown on the right (top row). Representative snapshots from the simulation at −1.0 V, 0 V and +1.0 V relative to the PZC (bottom row), shown from left to right. **b** Potential-dependent Raman spectra of coordination structures. **c, d** Potential-dependent contents of SSIP (blue), CIP (pink). SSIP, solvent-separated ion pairs; CIP, contact ion pairs. **e** Snapshots of the graphite-electrolyte interface obtained from constant-potential molecular dynamics (CPM MD) simulations at E = 0.0 V. **f** Comparison of interfacial structural characteristics at E = 0.0 V obtained from experiment (blue) and simulations (orange). Simulation data represent averages over 200 independent samples, with error bars representing the standard error of the mean. Minor discrepancies can be attributed to limitations of the OPLS/AA force field and residual uncertainties in both experiment and simulation.

**Fig. 3:**
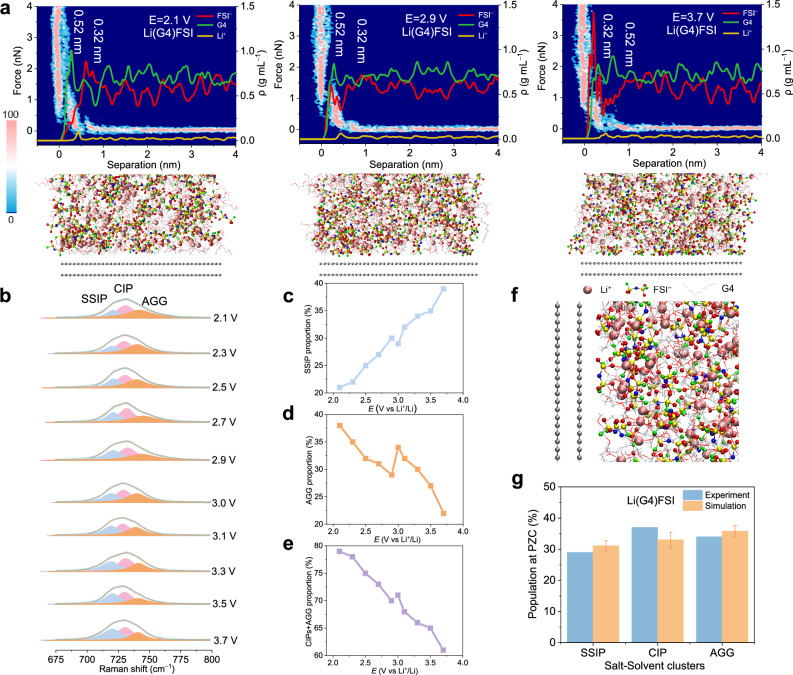
Characterization of the Li^+^ solvation structure in a high-concentration [Li(G4)][FSI] electrolyte. **a** Two-dimensional atomic force microscopy force-separation histograms from 20 independent force curves for [Li(G4)][FSI] acquired at potentials of *E* = 2.1 V_Li_, *E* = 2.9 V_Li_, and *E* = 3.7 V_Li_, with the probability distribution indicated by the color scale shown on the left. The corresponding ion number density profiles of FSI^–^ (red), G4 (green), and Li^+^ (yellow) obtained from MD simulations at PZC − 1.0 V, PZC, PZC + 1.0 V are shown on the right (top row). Representative snapshots from the simulation at −1.0 V, 0 V and +1.0 V relative to the PZC (bottom row), shown from left to right. **b** Potential-dependent Raman spectra of coordination structures. **c–e** Potential-dependent contents of SSIP (blue), AGG (orange), and CIP + AGG (purple). SSIP, solvent-separated ion pairs; CIP, contact ion pairs; AGG, aggregates. **f** Snapshots of the graphite-electrolyte interface obtained from constant-potential molecular dynamics (CPM MD) simulations at E = 0.0 V. **g** Comparison of interfacial structural characteristics at E = 0.0 V obtained from experiment (blue) and simulations (orange). Simulation data represent averages over 200 independent samples, with error bars representing the standard error of the mean. Minor discrepancies can be attributed to limitations of the OPLS/AA force field and residual uncertainties in both experiment and simulation.

In the low-concentration electrolyte, defined as the GCS regime, in situ AFM was used to probe the layered structure on the HOPG electrode interface, focusing on the effects of free solvent molecules and applied potential. Structural variations were examined at potentials negative of the PZC (*E* = 2.1 V_Li_ and 2.5 V_Li_), near the PZC (*E* = 2.9 V_Li_), and positive of the PZC (*E* = 3.3 V_Li_ and 3.7 V_Li_). Representative force-separation profiles at each potential are presented in Figs. 2a and 3a, with each profile comprising 20 individual curves. The rupture force is defined as the maximum force recorded at each step, where higher values generally reflect a greater degree of structural ordering, attributed to the stronger cohesive interactions within the ion layer^41^. The force increases as the separation decreases, revealing more distinct nanoscale structures near the surface. The width of each step observed in the force-separation (approach and retract) curves reflects the characteristic dimension of the interfacial species (anions, cations, or solvent molecules) involved in the layer rupture or rearrangement process, and thus serves as an indicator of the composition of the interfacial layer^42,43^. The AFM force-separation distribution of [Li(G4)_3_][FSI] reveals distinct interfacial structures at varying electrode potentials (Fig. 2a and Supplementary Figs. 11–15). At *E* = 2.9 V_Li_, near the PZC, Li(G4)^+^ cations accumulate at the HOPG interface due to the poor solvent affinity of the surface^44^. Upon shifting the potential negatively to *E* = 2.5 V_Li_, significant adsorption of free G4 molecules occurs at the interface, forming a step thickness of approximately 0.32 nm. Long-chain molecules like glymes tend to assemble into thinner interfacial layers, consistent with their planar geometry observed in previous AFM studies^45^. The second layer exhibits a step thickness of 0.50 nm, corresponding to the enrichment of Li(G4)^+^ ions, which effectively screens excess negative surface charge. Further decreasing the potential to *E* = 2.1 V_Li_, the innermost layer continues to exhibit substantial free G4 adsorption, while the adsorption configuration of Li(G4)^+^ shifts to a more tilted orientation due to steric hindrance, reducing the layer thickness. At positive polarization (*E* = 3.3 V_Li_ and 3.7 V_Li_), step thicknesses of ~0.31 and 0.33 nm were observed, which are attributed to the adsorption of FSI^**–**^ anions and free G4 molecules, effectively screening excess positive surface charge. Prompted by the EIS-determined PZC, we employed AFM to independently probe the interfacial environment, which confirmed the PZC assignment and provided direct evidence of potential-dependent interfacial structuring.

Surface-enhanced Raman spectroscopy (SERS) is a powerful technique for probing chemical information at electrochemical interfaces^46^. However, the Raman signals of molecules adsorbed on carbon electrodes are typically too weak for direct detection^47^. To address this, we designed and developed a gap-enhanced Raman spectroscopy in our previous work, which enables the investigation of potential-dependent solvation structures at the interface of six-layer graphene with graphite-like properties^48^. Combined with AFM force curve measurements, this approach provides a quantitative analysis of solvation structures in electrolytes. As shown in Supplementary Fig. 16, under mild negative polarization (*E* = 2.7 V_Li_), the scissoring CH_2_ group exhibits a Raman signal broadening band (1400-1500 cm^**–**1^) and a redshift compared to that of the pure solvent. Additionally, the Li(G4)^+^ crown ether-like breathing mode peak (868 cm^**–**1^) gradually intensifies^49^, indicating that the first interfacial layer consists of Li(G4)^+^ adsorption, while the second layer is composed of G4 molecules. Under stronger negative polarization (*E* shifting from 2.9 to 2.1 V_Li_), the CH_2_ peak profile gradually becomes similar to that of the pure solvent, and the peak assigned to the coupled C-C stretching and CH_2_ wagging modes^50,51^ redshift from 1145 cm^**–**1^ at *E* = 2.9 V_Li_ to 1135 cm^**–**1^ at *E* = 2.1 V_Li_ (Supplementary Fig. 17). This transition suggests a structural rearrangement at the interface, where free G4 molecules are adsorbed in the first layer, while Li(G4)^+^ forms the second layer. Additionally, spectral peaks observed at 260 cm^**–**1^ and 292 cm^**–**1^ are attributed to the rocking vibrations of S-F and SO_2_, respectively^52^. A closer inspection of these bands under positive polarization reveals a two-stage potential dependence (Supplementary Fig. 18). As the potential is increased from 3.0 to 3.3 V_Li_, the intensities of both the S-F and SO_2_ peaks rise, consistent with enhanced accumulation of FSI^**–**^ at the electrode interface. Upon further increasing the potential from 3.3 to 3.7 V_Li_, the S-F peak continues to grow in intensity while the SO_2_ peak decreases; concurrently the SO_2_ blueshifts from 292 cm^**–**1^ (*E* = 3.0 V_Li_) to 298 cm^**–**1^ (*E* = 3.7 V_Li_). These observations indicate a potential-driven reorientation of adsorbed FSI^**–**^ in which the S-F moiety approaches the electrode surface while the SO_2_ group tilts toward the solution. Such reconfiguration promotes a higher surface adsorption density of anions and alleviates interfacial steric hindrance, confirming that the first interfacial layer is dominated by specifically adsorbed FSI^**–**^. The coupled C-C stretching and CH_2_ wagging mode undergoes a blueshift as the potential increases, whereas the Li(G4)^+^ crown ether-like breathing mode peak weakens and eventually disappears. These observations suggest that the second layer consists of free G4 molecules, with alternating adsorption of counterions and free solvent molecules at the surface.

To provide an atomistic resolution of the interfacial structure and corroborate the experimental inferences, we performed molecular dynamics simulations using the Constant Potential Method (CPM), as shown in Fig. 2a. The simulation explicitly captures the potential-dependent restructuring of the electrical double layer (EDL) at the graphite-electrolyte interface. At potentials negative relative to the PZC, the simulated density profiles and snapshots reveal that the innermost Helmholtz plane is predominantly occupied by adsorbed free G4 solvent molecules rather than desolvated Li^+^ ions. These interfacial G4 molecules preferentially adopt a flattened orientation parallel to the basal plane, forming a dense solvent layer that is consistent with the ~0.32 nm step thickness observed in AFM. The solvated Li(G4)^+^ cations are statistically located in the second layer, separated from the electrode by this solvent cushion. Conversely, under positive polarization, the simulation shows the accumulation of FSI^**–**^ anions co-adsorbed with G4 molecules to screen the positively charged surface (Supplementary Fig. 19). These trends are in well agreement with the potential-dependent interfacial layering resolved by AFM. Beyond structural layering, the simulations further provide molecular-level insight into the solvation structures probed by Raman spectroscopy. The coordination of Li^+^ with solvent molecules gives rise to distinct solvation structures including SSIP, CIP, and AGG, which can be identified through Raman spectral peaks^53–55^. Consistent with the Raman analysis in Fig. 2b–d, the EDL is dominated by SSIP and free G4 molecules, with only a minor contribution from CIP and a negligible population of AGG. In particular, in the [Li(G4)_3_][FSI] electrolyte, AGG are virtually absent at the interface (Fig. 2e), reflecting a relatively loose solvation sheath. At the PZC, the simulated fractions of SSIP, CIP, and AGG quantitatively agree with the experimentally extracted values within ~10% (Fig. 2f), offering a clear physical explanation for the distinct structural ordering and layer compositions detected by in situ spectroscopic observations. Across other applied potentials, despite quantitative deviations, the simulated evolution of SSIP and CIP follows the Raman trends, exhibiting clear potential dependence (Supplementary Fig. 20).

### Aggregated anionic structures in the Non-GCS Regime

High-concentration electrolytes (HCEs) exhibit unique solvation structures, differing from conventional electrolytes with free solvent molecules. This distinct solvation behavior significantly impacts interfacial chemistry and electrochemical performance in batteries^56^. However, these interfacial solvation structures and their interactions remain incompletely understood, necessitating further exploration. To this end, we employed AFM to study the high-concentration [Li(G4)][FSI] system with the non-GCS regime (Fig. 3a and Supplementary Figs. 21–25). At *E* = 2.1 V_Li_ and 2.5 V_Li_, [Li(G4)][FSI] forms a two-layered interfacial structure, with the first layer 0.52 nm and the second 0.32 nm. These step thicknesses align well with the sizes of Li(G4)^+^ and FSI^**–**^ ions^57^. At *E* = 2.9 V_Li_, near the PZC, the force curve data remain comparable to those at *E* = 2.5 V_Li_ and can be interpreted similarly. At more positive potentials (*E* = 3.3 V_Li_ and 3.7 V_Li_), the applied potential to the HOPG electrode reorganizes the interfacial layers. This rearrangement arises from the electrostatic repulsion of cations and the simultaneous attraction of anions. Consequently, the first step thickness decreases to 0.32 nm, indicating FSI^**–**^ enrichment at the innermost interfacial layer. Meanwhile, the second step thickness increases to 0.52 nm, corresponding to Li(G4)^+^ accumulation in the outer layer. AFM provides direct structural evidence of increased EDL thickness in concentrated electrolytes, reinforcing the anomalous screening behavior revealed by our capacitance measurements.

Simultaneously, gap-enhanced Raman spectroscopy was employed to obtain chemical insights into the electrolyte interface. In the [Li(G4)][FSI] system (Supplementary Fig. 26), applying a positive electrode potential relative to the PZC increases the intensity of the S-F peak while decreasing that of the SO_2_ peak. This trend suggests a transition in the adsorption orientation of FSI^**–**^ anions from a flat-lying to a tilted configuration. Meanwhile, the Raman signal of the CH_2_ bending/scissoring modes (1400–1500 cm^**–**1^) progressively weakens and eventually disappears, indicating the desorption of Li(G4)^+^ cations from the innermost layer. Conversely, when a potential negative than the PZC is applied, the S-F peak intensity decreases as FSI^**–**^ anions rotate and desorb from the innermost layer. Simultaneously, the Raman signal of the CH_2_ bend intensifies with the negative shift of the potential, suggesting that Li(G4)^+^ cations adopt a flat adsorption configuration at the surface. These findings indicate an alternating adsorption of cations and anions at the electrode interface. In this high-concentration electrolyte, the high salt-to-solvent ratio causes anions to remain within the solvation sheath, enhancing Li-anion affinity. Consequently, the solvation structures are primarily composed of CIP and AGG, with only a minor presence of SSIP (Fig. 3b–e). Under further negative polarization, anions remain within the EDL, actively participating in interfacial interactions prior to SEI formation. Furthermore, the CPM simulation captures a high degree of ionic correlation within the double layer; unlike the diffuse distribution in dilute systems, the interfacial species in the high-concentration electrolyte regime exist predominantly as CIP and AGG (Fig. 3f and g). Their potential-dependent evolution is also consistent with the experimental trends (Supplementary Fig. 27).

In contrast to the dilute regime, where free solvent molecules dominate the interface (Fig. 3a and Supplementary Fig. 28), the simulation reveals that in highly-concentrated electrolytes the scarcity of free G4 molecules precludes the formation of a solvent-passivated layer. Instead, the interface exhibits a distinct alternating ion-layering mechanism. At potentials negative of the PZC, the innermost Helmholtz plane is populated primarily by bulky Li(G4)^+^ cations, corresponding to the larger step thickness (~0.52 nm) detected by AFM. Conversely, under positive polarization, the interface becomes saturated by a compact layer of FSI^**–**^ anions, aligning perfectly with the reduced step thickness (~0.32 nm) (Supplementary Fig. 29). These results demonstrate that electrolyte concentration exerts a decisive control over the interfacial nanostructure by shifting the positions of discrete layering steps, reflecting changes in the physical dimensions and composition of ion accumulation near the electrode. This concentration-dependent structural reorganization is further manifested in the mechanical response of the EDL. Specifically, comparison of rupture forces measured in [Li(G4)][FSI] and [Li(G4)_3_][FSI] (Supplementary Fig. 30) shows that the non-GCS regime sustains larger rupture forces than the classical GCS regime. This observation indicates the formation of a more ordered layered structure within the EDL, exhibiting an anomalous screening length. Such an “ion-crowding” effect, accurately reproduced by the CPM simulation, explains the enhanced rupture forces and the pronounced, potential-driven interfacial restructuring. By contrast, in electrolytes containing excess free solvent, anions are largely excluded from the Li⁺ solvation sheath and do not directly participate in the innermost interfacial layer. Together, these findings clarify how the solvation state of lithium ions and the availability of free solvent dictate the nanoscale organization of the EDL, with direct consequences for interfacial electrochemical reactions. In particular, during solid electrolyte interphase (SEI) formation on the anode, the EDL structure plays a decisive role in steering competing reduction pathways, as the spatial proximity of electrolyte components within the EDL governs their likelihood of incorporation into the SEI. Bent and co-workers demonstrated that species positioned closer to the electrode are preferentially incorporated into the SEI during electrolyte decomposition^58^.

### Li^**+**^ transport mechanism and electrochemical performance

**Fig. 4:**
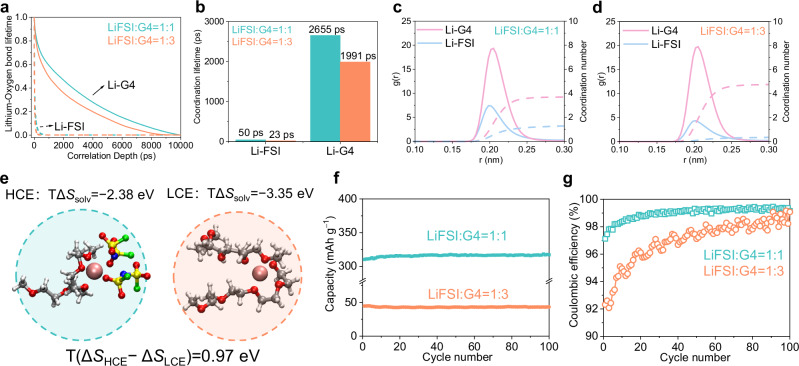
The connection between thermodynamics and dynamics. **a** [Li(G4)][FSI] (green) and [Li(G4)_3_][FSI] (orange) lithium-oxygen bond autocorrelation functions. **b** The coordination lifetime of Li-FSI and Li-G4 in [Li(G4)][FSI] and [Li(G4)_3_][FSI]. **c, d** Radial distribution functions and coordination numbers of [Li(G4)][FSI] (**c**) and [Li(G4)_3_][FSI] (**d**) from machine learning molecular dynamics (MLMD) results. **e** Average solvation structure of [Li(G4)][FSI] and [Li(G4)_3_][FSI] extracted from MLMD analysis, *S*_solv_ represents solvation entropy, HCE: high concentration electrolyte, LCE: low concentration electrolyte. **f** Cycle performance of [Li(G4)][FSI] and [Li(G4)_3_][FSI]. **g** Coulombic Efficiency of graphite | |Li cells using [Li(G4)][FSI] and [Li(G4)_3_][FSI], measured with a charge rate of 1 C and a discharge rate of 0.1 C.

Interfacial solvation structures not only define the local chemical environment of Li^+^ but also directly regulate its desolvation process and transport kinetics. To investigate these effects at the molecular scale, we used machine learning molecular dynamics (MLMD) to analyze solvation dynamics, focusing on the coordination lifetimes of anions and solvent molecules surrounding Li^+^. The dynamic characteristics of Li^+^ coordination were quantified using the time autocorrelation function (TACF)^59^, allowing for the evaluation of Li-G4 and Li-FSI coordination lifetimes. In the [Li(G4)][FSI] (Fig. 4a, b and Supplementary Fig. 31), G4 molecules exhibit prolonged binding with Li^+^, indicative of strong interactions and slow solvent exchange within the solvation structure. Meanwhile, FSI^**–**^ anions consistently participate in the formation of the primary solvation sheath and display significant exchange behavior, with coordination numbers of 3.7 for Li-G4 and 1.3 for Li-FSI (Fig. 4c). In contrast, in the [Li(G4)_3_][FSI], multiple G4 molecules coordinate around Li^+^, but their binding time is relatively short, and solvent exchange occurs more frequently. FSI^**–**^ anions only occasionally contribute to the primary solvation sheath and exhibit negligible exchange dynamics, with Li-G4 and Li-FSI coordination numbers of 4.7 and 0.3, respectively (Fig. 4d). Thus, increasing the lithium salt concentration significantly enhances the dynamic exchange frequency of anions in the Li^+^ solvation sheath while reducing the number of coordinated G4 molecules. This promotes the desolvation process and facilitates anion participation in interfacial reactions near the electrode surface, which is critical for improving interfacial stability.

The rearrangement and exchange of anions during Li^+^ migration is a dynamic process governed by the stability of the solvation structure and the associated thermodynamic parameters. Among these parameters, entropy plays a crucial role, as an increase in entropy generally corresponds to a greater number of accessible microstates and enhanced kinetic exchange rates, thereby influencing Li^+^ diffusion, interfacial reactions, and overall electrochemical performance. The Gibbs free energy (G) is defined as

4 $${{\rm{G}}}={{\rm{H}}}-{{\rm{TS}}},$$

where a larger entropy increase affects G through the −TS term, effectively lowering its value. To quantify the concentration-dependent evolution of solvation entropy, we employed the two-body excess entropy (S_2_) approximation derived from liquid-state statistical mechanics. This framework captures the entropy contribution arising from pairwise spatial correlations and has been widely used to characterize structural ordering in complex fluids and ionic systems^60,61^. Specifically, the solvation entropy associated with Li^+^ was evaluated from the radial distribution functions between Li^+^ and surrounding species, according to

5 $${{{\rm{S}}}}_{2}=-2{{\rm{\pi }}}{{\rm{\rho }}}{{{\rm{k}}}}_{{{\rm{B}}}}{\int }_{0}^{{\infty }}\left[{{\rm{g}}}\left({{\rm{r}}}\right){\mathrm{ln}}{{\rm{g}}}\left({{\rm{r}}}\right)-{{\rm{g}}}\left({{\rm{r}}}\right)+1\right]{{{\rm{r}}}}^{2}{{\rm{dr}}}$$

where g(r) is the radial distribution function, ${{\rm{\rho }}}$ is the number density, and ${{{\rm{k}}}}_{{{\rm{B}}}}$ is the Boltzmann constant.

Within this formalism, a more negative S_2_ indicates corresponds to a more rigid environment, while a less negative value indicates a more labile sheath. At 298.15 K, relative to the ideal gas state, the solvation entropy of the high-concentration [Li(G4)][FSI] system was −93 ${{{\rm{k}}}}_{{{\rm{B}}}}$ ( − 2.38 eV), whereas that of the low-concentration [Li(G4)_3_][FSI] system was −129 ${{{\rm{k}}}}_{{{\rm{B}}}}$ ( − 3.35 eV), yielding T(Δ*S*_HCE_–Δ*S*_LCE_) = 0.97 eV (Fig. 4e). As salt concentration increases, the system contains almost no free solvent, and the entropy gain promotes the formation of AGG structures while enhancing the prevalence of anion-rich solvation configurations. Entropy-mediated effects likely dominate solvation structure evolution, as the directional nature of entropy dictates that solvation sheaths do not form randomly but instead adopt a preferentially structured organization. Subsequently, we examined the impact of entropy tuning on battery cycling performance by recording galvanostatic charge-discharge curves of graphite | |Li coin cells. Figure 4f and 4g show the cycling performance and Coulombic efficiency of [Li(G4)][FSI] and [Li(G4)_3_][FSI]. Notably, [Li(G4)][FSI] achieves a higher reversible capacity of 320 mAh g^**–**1^, approaching the theoretical limit of fully lithiated graphite (LiC_6_, 370 mAh g^**–**1^). In contrast, the [Li(G4)_3_][FSI]-based cell shows poor reversibility, yielding only 50 mAh g^**–**1^. Furthermore, during the initial charge-discharge cycles, its Coulombic efficiency is lower than that of [Li(G4)][FSI], which is closely associated with solvent co-intercalation at the anode. Specifically, solvent co-intercalation disrupts the graphite interlayer structure, increasing direct exposure of the anode to electrolyte solvent molecules and triggering the irreversible decomposition of G4 on the anode surface. This process accelerates undesirable electrolyte decomposition, resulting in an initial irreversible capacity loss and ultimately lowering the overall Coulombic efficiency.

**Fig. 5:**
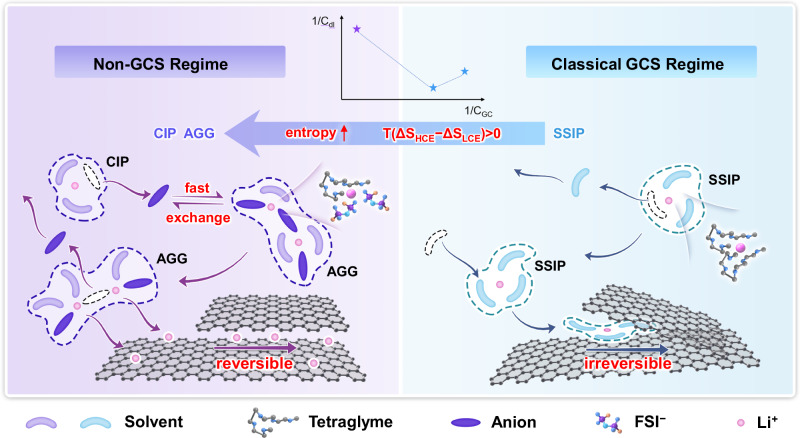
Schematic illustration of the Li^+^ transport mechanism and charge transfer process in electrolytes with different concentrations. Left: Purple, in the highly concentrated electrolyte (HCE) exhibiting a non-Gouy−Chapman−Stern (non-GCS) regime. Strong ion-ion correlations and anion-rich solvation structures promote efficient Li^+^ desolvation at the electrode interface, enabling a reversible Li^+^ intercalation/deintercalation process in graphite. Right: Blue, low-concentration electrolyte (LCE) following the classical Gouy−Chapman−Stern (GCS) regime, insufficient Li^+^ desolvation leads to solvent co-intercalation into graphite, causing graphite exfoliation and irreversible interfacial processes. The inset illustrates the transition from the non-GCS regime to the classical GCS regime through the relationship between the diffuse-layer capacitance (*C*_GC_) and double-layer capacitance (*C*_dl_). The increase in solvation entropy, expressed as T(Δ*S*_HCE_ − Δ*S*_LCE_) > 0, drives the evolution from SSIP-rich to CIP/AGG-rich solvation structures. Pink spheres represent Li⁺ ions, curved solvent molecules represent tetraglyme (G4). FSI^−^ bis(fluorosulfonyl)imide anion, CIP contact ion pair, AGG aggregate, SSIP solvent-separated ion pair.

Based on in situ spectroscopic experiments (Figs. 2b–d and 3b–e) and MLMD calculations (Fig. 4a–d), we elucidated the entropy-mediated mechanism governing solvation structure evolution and battery performance by analyzing the dynamic exchange frequency within the solvation sheath (Fig. 5). In the non-GCS regime, high solvation entropy promotes the formation of CIP and AGG solvation structures at the interface. During Li^+^ transport, a G4 molecule remains coordinated as Li^+^ moves from one coordinating anion to the next due to the fast exchange. This molecular-level transient kinetic process is characterized by a higher dynamic exchange frequency between Li^+^ and FSI^**–**^ than that between Li^+^ and G4, significantly reducing transport barriers in the electrolyte. The abundance of AGG structures indicates strong ion-ion interactions, lowering the interfacial desolvation energy^62,63^. With minimal free solvent present, AGG or CIP structures approaching the electrode-electrolyte interface face a reduced desolvation barrier, effectively suppressing solvent co-intercalation. After 100 cycles, the delithiation capacity remained at 320 mAh g^**–**1^, indicating stable Li^+^ intercalation and deintercalation in the graphite electrode. This suggests the formation of a favorable interface between the graphite electrode and the Li(G4)FSI electrolyte. In contrast, in the classical GCS regime, lower solvation entropy favors the formation of a SSIP-rich interface. When free G4 molecules approach Li^+^, a pre-coordinated solvent molecule is displaced. This continuous and rapid exchange process leads to dominant Li^+^-G4 interactions at the interface, with negligible Li^+^-FSI^**–**^ exchange, resulting in the accumulation of solvated cations. The hindered desolvation process fails to prevent solvent co-intercalation, ultimately compromising battery cycling stability. Thus, we propose that electrolyte concentration-dependent solvation structure evolution is fundamentally governed by entropy-mediated Li^+^ interactions with anions and solvent molecules. By tuning their dynamic exchange frequencies and solvation structures, the dynamic exchange of anionic aggregates can be promoted. In this mechanism, Li^+^-anion exchange plays a crucial role: the effective participation of anions not only facilitates the formation of a well-defined solvation structure but also significantly lowers the desolvation energy barrier, thereby influencing interfacial Li^+^ transport kinetics. This mechanism provides a design strategy for improving electrochemical stability and optimizing battery performance.

## Discussion

In summary, a combined experimental and computational approach was taken to understand the influence of electrolyte concentration on the interfacial electrical double layer in non-aqueous lithium-ion battery systems. The electrolyte concentration fundamentally alters the EDL via an entropy-mediated reorganization of the interfacial solvation structure. This entropic contribution facilitates the formation of ion-aggregated configurations at the electrode interface, leading to an anion-enriched solvation environment. Notably, the Parsons–Zobel plot exhibits nonmonotonic behaviors, revealing a clear deviation from classical GCS behavior. This deviation is consistent with the presence of interfacial structural reorganization in concentrated electrolytes, where entropy-mediated effects contribute to anomalous EDL screening. Furthermore, dynamic exchange within the solvation sheath was identified during ion transport. When the exchange frequency between Li^+^ and FSI^**–**^ surpasses that between Li^+^ and G4, the number of anions in the solvation sheath increases while the coordinated G4 molecules decrease. This shift promotes desolvation, thereby enhancing the efficiency and reversibility of Li^+^ intercalation/deintercalation. This study advances understanding of electrolyte systems, demonstrating that entropy-mediated effects extend beyond enhancing molecular diversity to significantly influence battery performance by regulating solvation structures and dynamics. The entropy-mediated mechanism unveiled here provides a theoretical framework for the design of next-generation high-concentration electrolytes.

## Methods

### Materials

Tetraglyme (G4, Suzhou Duoduo Chemical Technology Co., Ltd.‌, purity ≥99.8 %) and Lithium Bis(fluorosulfonyl)imide (LiFSI, Nippon Shokubai, purity ≥99.9 %) were mixed in a glovebox under an argon atmosphere (VG1500, H_2_O < 0.1 ppm, O_2_ < 0.1 ppm) with molar ratios of LiFSI to G4 set at 1:1 and 1:3, respectively. The mixtures were stirred at 298 ± 1 K for 24 hours, yielding homogeneous liquids. These liquids, referred to as [Li(G4)][FSI] and [Li(G4)_3_][FSI], were stored and handled inside the glovebox to avoid moisture contamination. Lithium electrodes (≥99.9 %) were purchased from China Energy Lithium Co., Ltd. The graphite electrodes were purchased directly from Shenzhen Kejing STAR Technology Company.

### Assembly of Au@SiO_2_/graphene/Au Structure

The fabrication began by annealing a 25 µm-thick copper foil in a hydrogen atmosphere at 1000 °C. Graphene was subsequently grown on the foil in a H_2_/CH_4_ environment while maintaining the temperature at 1150 °C. After the growth process, the system was cooled to room temperature (298 ± 1 K)^64^. By modifying the growth duration, multilayer graphene could be produced. A layer of PMMA was then spin-coated onto the graphene/Cu foil and cured by heating to approximately 100 °C. The copper foil was removed using an ammonium persulfate etchant, leaving the PMMA/graphene layer floating on the solution. This layer was thoroughly rinsed with deionized water before being transferred onto a silicon substrate pre-coated with 100 nm of gold by thermal evaporation. Once dried, the PMMA was dissolved using acetone, resulting in a graphene/Au substrate at 298 ± 1 K^65^. SHINs^66^ were dispersed in ultrapure water, deposited onto the graphene/Au surface, and dried under vacuum. The electrochemical cell was assembled in an argon glovebox with H_2_O and O_2_ levels kept below 0.1 ppm at 298 ± 1 K.

### In situ Raman experiment

Raman microscopy (Nanophoton Corporation Vis-NIR-XU) with a 785 nm laser was utilized to capture Raman spectra. A graphene/Au substrate (10 mm × 10 mm) decorated with SHINs was prepared as the working electrode, while lithium foils served as both reference and counter electrodes. The system used 500 μL of electrolyte and was assembled in an argon glovebox at 298 ± 1 K. Raman measurements were conducted using a 50× objective lens with a numerical aperture of 0.45 and a 300 grooves/mm grating. The laser power was kept around 0.6 mW, and each measurement had an accumulation time of 60 seconds.

### Electrochemical AFM measurement

All force-distance measurements were performed using a Bruker Icon atomic force microscope operated inside an argon-filled glovebox (MIKROUNA, H_2_O < 0.1 ppm, O_2_ < 0.1 ppm) to eliminate moisture and oxygen interference. The electrochemical AFM (EC-AFM) configuration consisted of a three-electrode cell integrated with the AFM system. The working electrode, reference electrode, and counter electrode were immersed in the electrolyte and connected to a potentiostat (CHI660E, Chenhua Instruments) for constant-potential control. A CSG30 cantilever (TipsNano) with a spring constant of 0.6 N m^**–**1^ was used to acquire force curves. Before each experiment, the cantilever sensitivity and spring constant were calibrated against a standard reference surface (e.g., sapphire) under an inert atmosphere to ensure accurate force quantification. A freshly cleaved 20 mm × 20 mm highly oriented pyrolytic graphite (HOPG, ZYB grade) served as the working electrode, while two lithium wires were used as the counter and reference electrodes, respectively. The electrochemical cell contained 600 μL of electrolyte, and at least 20 force-distance curves were collected at each applied potential. The processing of the force curve data is described in the Supplementary Information. All measurements were conducted at 298 ± 1 K.

### Electrochemical measurements

Cyclic voltammetry experiments were performed using a three-electrode setup on an electrochemical workstation (CHI760E, Chenhua Instruments). A freshly cleaved HOPG (10 mm×10 mm) was used as the working electrode, and two lithium strips were employed as the counter and reference electrodes, respectively. The electrolyte volume was ~600 µL, and all reported potentials were referenced to the Li⁺/Li electrode. Electrochemical impedance spectroscopy (EIS) measurements were conducted using a PGSTAT128N (Metrohm) electrochemical workstation, with the frequency range spanning from 100 kHz to 100 Hz and an amplitude of 10 mV. All measurements were conducted at 298 ± 1 K. The EIS data were analyzed using RelaxIS (version 3.0.15, rhd Instruments GmbH & Co. KG, Darmstadt, Germany).

### Measurements of electrochemical performances

All electrochemical tests were conducted using CR2016-type coin cells assembled in an Ar-filled glovebox. Each cell employed a single layer of Celgard 2325 as the separator and contained 100 μL of electrolyte. Galvanostatic charge-discharge measurements were carried out using a LANHE CT2001A battery testing system, with charging performed at 1 C and discharging at 0.1 C. For graphiteⅡLi half-cells, the voltage window was set to 0.005 ~ 2.0 V (vs. Li^+^/Li). All electrochemical measurements were carried out at 298 ± 1 K.

### Calculation methods

Classical Molecular Dynamics (CMD) simulations were employed to investigate the interfacial structure and dynamics between a graphite electrode and LiFSI/G4 electrolytes. The electrolyte systems were constructed with molar ratios of LiFSI:G4 at 1:1 and 1:3. To ensure adequate statistical sampling and minimize finite-size effects, each simulation system contained approximately 15,000 atoms^67^. The intermolecular interactions for the electrolyte species were described as follows: the parameters for G4 were generated using the LigParGen web server^68^ within the OPLS-AA (Optimized Potentials for Liquid Simulations-All Atom) framework^69^, whereas the bonded and nonbonded parameters for LiFSI were taken from the ionic-liquid force field developed by Canongia Lopes and Pádua^70^. The atomic partial charges of LiFSI were recalculated using the restrained electrostatic potential (RESP)^71^ scheme based on Gaussian 16 calculations at the B3LYP/6-31 + G(d) level^72–75^ with electrostatic potential fitting, and the charges of symmetry-equivalent atoms were averaged before being assigned in the simulations. As shown in Fig. S32, we further performed CMD simulations for Li(G4)FSI electrolytes using the original ILFF, the 0.8 charge-scaled ILFF model, and the RESP-refitted force field used in this work and the Li^+^–O_FSI_^**–**^ coordination numbers were compared with the MLMD benchmark. The results show a consistent trend ILFF overestimates Li^+^–O_FSI_^**–**^ coordination charge scaling reduces the coordination as expected but still does not reach the MLMD result while the RESP-refitted model provides the best agreement with MLMD. Therefore, the RESP-refitted force field was used in the subsequent EDL simulations. A critical feature of this study is the realistic treatment of the electrode electrostatics using the Constant Potential Method (CPM)^76,77^. Unlike conventional fixed-charge simulations, the CPM allows the partial charges on the graphite electrode atoms to fluctuate dynamically. At every time step, the charge distribution on the electrode surface is updated self-consistently to maintain the specified electrode potential while minimizing the total energy of the system. This approach accurately captures the electronic polarization of the electrode and the complex structure of the electrical double layer (EDL) under applied voltages.2.2. Simulation Protocol. All MD simulations were carried out using the LAMMPS package^78^. The equations of motion were integrated using the velocity Verlet algorithm with a time step of 2.0 fs. The temperature was maintained at 300 K using a Langevin thermostat with a damping parameter of 100 fs. Although the integration was performed in the NVE ensemble, the coupling with the Langevin thermostat effectively realized a canonical (NVT) sampling distribution^79^. The simulation protocol proceeded as follows. The system was first equilibrated for 10 ns at 0 V, which was taken as the reference potential in our CPM setup, to relax the interfacial structure. Following equilibration, production runs were performed for 10 ns under various constant potential conditions. The electrode potentials were set to ϕ= 0, 0.5, and 1.0 V (corresponding to effective cell voltages of 0, 1, and 2 V). Trajectories were saved every 1.0 ps for post-analysis to ensure sufficient sampling of the ion dynamics and interfacial properties. The fractions of CIP, SSIP, and AGG were determined by analyzing the local solvation structures of Li^+^ ions located within 10 Å of the graphite surface at each applied potential. Specifically, the classification was based on the number of coordinated FSI⁻ anions, as defined in the literature^80^.

Minor quantitative discrepancies are observed between the simulation and Raman results in the relative populations of CIP, SSIP, and AGG. These differences likely arise from the intrinsic limitations of the theoretical model and finite sampling in molecular dynamics simulations. Given the complex and dynamic nature of ionic association, the limited spatiotemporal sampling inevitably introduces statistical uncertainties in the extracted solvation populations. Given the complex dynamics of ionic association, this finite spatiotemporal scale inevitably introduces some statistical error. Nevertheless, the overall trend and the physical picture of the solvation structure remain consistent between the simulation and experiment. Moreover, the ionic conductivity was recalculated using the Einstein–Helfand formalism^81^, which explicitly accounts for correlated ionic motion. The resulting conductivity values remain in agreement with the experimental measurements, confirming that the simulations capture the essential macroscopic transport behavior and provide a reliable basis for qualitative analysis.

The simulated systems included 1:1 and 1:3 molar ratios of G4 (tetraglyme) to LiFSI, modeled using the universal machine learning potential (uMLP) for electrolytes^82^. The 1:1 system contained 43 G4 and 43 LiFSI molecules, while the 1:3 system consisted of 51 G4 and 17 LiFSI molecules. MLMD simulations were conducted using the Large-scale Atomic/Molecular Massively Parallel Simulator (LAMMPS) package. The densities of electrolytes under different temperatures were calculated using the NpT ensemble. The time step was set to 0.5 fs, and the pressure was maintained at 1 bar. The NpT ensemble simulations utilized the Nose-Hoover thermostat for temperature control and the Parrinello-Rahman barostat for pressure control, with temperature and stress damping parameters set to 50 fs and 500 fs, respectively. Subsequently, NVT ensemble simulations were performed for over 5 ns to compute ionic conductivity at various temperatures. The NVT ensemble employed the Nose-Hoover thermostat for temperature control, with the temperature damping parameter set to 50 fs. To compute solvation lifetimes, 10 ns NVT MLMD trajectories were generated for both systems, and the Time Autocorrelation Function was analyzed using the TRAVIS software.

### Reporting summary

Further information on research design is available in the Nature Portfolio Reporting Summary linked to this article.

## Supplementary information

Supplementary information

Reporting Summary

Transparent Peer Review file

## Source data

source data

## Data Availability

The data that support the findings of this study have been included in the main text and Supplementary Information. Source data are provided with this paper.
