# Supplementary material for: Nonmonotonic Screening and Solvation Dynamics of the Electrical Double Layer in Concentrated Lithium Salt Electrolytes
Source: Nat Commun. 2026 Jul 29;17:7587. doi: 10.1038/s41467-026-75999-2 (PMC13421668; doi:10.1038/s41467-026-75999-2)
Supplement: Supplementary file 1 — Supplementary information [file 41467_2026_75999_MOESM1_ESM.pdf]

Supplementary Information

Nonmonotonic Screening and Solvation Dynamics of the Electrical Double Layer in Concentrated Lithium Salt Electrolytes

Xiaoting Yin[1#], Feng Wang[2#], Zengming Zhang[3], Ru-Yu Zhou[1], Zhaobin Chen[1], Tairui Wu[1], Li Zhang[1], Jun Huang[3,4]*, Deyin Wu[1]*, Jun Cheng[1]*, Bingwei Mao[1], Jiawei Yan[1]*

[1]State Key Laboratory of Physical Chemistry of Solid Surfaces, College of Chemistry and Chemical Engineering, Xiamen University, Xiamen 361005, China
[2] Laboratory of AI for Electrochemistry (AI4EC), IKKEM, Xiamen 361005, China
[3]Institute of Energy and Climate Research, IET-3: Theory and Computation of Energy Materials, Forschungszentrum Jülich GmbH, 52425 Jülich, Germany
[4]Theory of Electrocatalytic Interfaces, Faculty of Georesources and Materials Engineering, RWTH Aachen University, Aachen 52062, Germany

*e-mail: jwyan@xmu.edu.cn; chengjun@xmu.edu.cn; dywu@xmu.edu.cn; ju.huang@fz-juelich.de

Suppl. Note 1. The detailed derivation of calculation C_{GC}[1-3]

The Poisson-Boltzmann (PB) equation describes the distributions of the electric potential and the ion concentrations in the electrolyte solution. Poisson equation reads,

$$\nabla\left(\epsilon_{\mathrm{s}}^{\mathrm{b}}\nabla\phi\right) = -\sum_i z_i F c_i \tag{S1}$$

where $\epsilon_{\mathrm{s}}^{\mathrm{b}}$ is the bulk dielectric permittivity of the electrolyte solution, ϕ the electric potential, z_i the charge number of ion i, F the Faraday's constant, c_i the concentration of ion i. Boltzmann equation further connects c_i and ϕ,

$$c_i = c_i^{\mathrm{b}} \exp\left(-\frac{z_i F}{RT}\phi\right) \tag{S2}$$

where c_i^{b} is the concentration of ion i in the solution bulk, R the gas constant, T the temperature. For a monovalent electrolyte solution in a one-dimensional space, the PB equation is rewritten as,

$$\nabla\left(\epsilon_{\mathrm{s}}^{\mathrm{b}}\nabla\phi\right) = -Fc^{\mathrm{b}}\left(\exp\left(-\frac{F\phi}{RT}\right) - \exp\left(\frac{F\phi}{RT}\right)\right) \tag{S3}$$

where c^{b} the concentration of total anions (cations) in the solution bulk. The dimensionless form of the PB equation is shown as,

$$\frac{\partial^2 U}{\partial X^2} = \sinh(U) \tag{S4}$$

with the dimensionless quantities, $U = F\phi/RT,\ X = x/\lambda_{\mathrm{D}}$, and the Debye length $\lambda_{\mathrm{D}} = \sqrt{\frac{RT\epsilon_{\mathrm{s}}^{\mathrm{b}}\epsilon_0}{2F^2 n_{\mathrm{i}}^{\mathrm{b}}}}$.

The boundary conditions to close Eq. (S4), a second-order differential equation, are,

$$U(X = 0) = U_{\mathrm{HP}} \tag{S5}$$

$$U(X = \infty) = 0 \tag{S6}$$

where $X = 0$ represents the left boundary, at the HP, and $X = \infty$ is the right boundary, in the solution bulk. U_{HP} can be calculated from the electrode side,

$$\phi_{\mathrm{HP}} = E_{\mathrm{M}} - E_{\mathrm{pzc}} + \left(\frac{\partial\phi}{\partial x}\right)_{x=0^+} \frac{\epsilon_{\mathrm{s}}^{\mathrm{b}}}{\epsilon_{\mathrm{HP}}} \delta_{\mathrm{HP}} \tag{S7}$$

where ϵ_{HP} and δ_{HP} are the dielectric permittivity and the thickness of the space between the electrode and the HP, respectively. The coefficient $\epsilon_{\mathrm{s}}^{\mathrm{b}}/\epsilon_{\mathrm{HP}}$ is resultant from the following equality in terms of surface charge density on the electrode surface,

$$\sigma_{\mathrm{M}} = -\epsilon_{\mathrm{s}}^{\mathrm{b}} \left(\frac{\partial\phi}{\partial x}\right)_{x=0^+} = -\epsilon_{\mathrm{HP}} \left(\frac{\partial\phi}{\partial x}\right)_{x=0^-} \tag{S8}$$

Solving Eq. (S4) in the following steps,

$$2\frac{\partial^2 U}{\partial X^2}\frac{\partial U}{\partial X} = 2\sinh(U)\frac{\partial U}{\partial X} \tag{S9}$$

$$\mathrm{d}\left(\frac{\partial U}{\partial X}\right)^2 = \mathrm{d}(2\cosh U) \tag{S10}$$

$$\left(\frac{\partial U}{\partial X}\right)_{X=0^+}^{2} = \left(2\sinh\left(\frac{U_{\mathrm{HP}}}{2}\right)\right)^2 \tag{S11}$$

we obtain the relationship between the excess free surface charge density and the electric potential at the HP,

$$\sigma_{\mathrm{free}} = -\int (c_+ - c_-)F\mathrm{d}x = -\epsilon_{\mathrm{s}}^{\mathrm{b}}\left(\frac{\partial\phi}{\partial x}\right)_{x=0^+} = \frac{2\epsilon_{\mathrm{S}}RT}{F\lambda_{\mathrm{D}}}\sinh\left(\frac{F\phi_{\mathrm{HP}}}{2RT}\right) \tag{S12}$$

The Bikerman-Poisson-Boltzmann (BPB) model treats the electrolyte solution using the lattice-gas approach. Each ion occupies a volume of a_{t}^3, where a_{t} is the lattice size. The maximum particle number density is $n_{\mathrm{t}} = a_{\mathrm{t}}^{-3}$. The electrochemical potential for ion i reads,

$$\tilde{\mu}_i = \mu_i^0 + z_i e_0 \phi + k_{\mathrm{B}}T\ln\frac{a_{\mathrm{t}}^3 n_i}{1 - a_{\mathrm{t}}^3\sum_i \gamma_i n_i} \tag{S13}$$

where μ_i^0 is the chemical potential under standard conditions, e_0 is the elementary charge, $\mathrm{k_B}$ the Boltzmann constant, n_i the number density of ion i, $\gamma_{\mathrm{i}} = \left(\frac{2r_{\mathrm{i}}}{R_{\mathrm{S}}}\right)^3$ is the relative size of ions referenced to solvent with r_i being the radius of solvated ion and R_{s} the diameter of solvent. $(1 - a_{\mathrm{t}}^3\sum_i \gamma_i n_i)/a_{\mathrm{t}}^3$ the number density of solvent

molecules. For a binary electrolyte solution, we have $n_{\mathrm{a}}^{0}=n_{\mathrm{c}}^{0}=n_{0}^{\mathrm{b}}$, with n_{0}^{b} the number density of total anions (cations) in the solution bulk. Under equilibrium conditions, the electrochemical potential for ion i is uniform in the whole EDL,

$$\tilde{\mu}_i=\mu_i^0+z_i e_0\phi+k_{\mathrm{B}}T\ln\frac{{a_{\mathrm{t}}}^3 n_i}{1-{a_{\mathrm{t}}}^3\sum_i\gamma_i n_i}=\mu_i^0+k_{\mathrm{B}}T\ln\frac{{a_{\mathrm{t}}}^3 n_0^{\mathrm{b}}}{1-{a_{\mathrm{t}}}^3\sum_i\gamma_i n_0^{\mathrm{b}}} \tag{S14}$$

The number density of ion i is obtained as,

$$n_{\mathrm{c/a}}=\frac{n_0^{\mathrm{b}}\exp\left(\frac{\mp z_i e_0\phi}{k_{\mathrm{B}}T}\right)}{1+\frac{v}{2}\left(\gamma_{\mathrm{c}}\exp\left(\frac{-z_i e_0\phi}{k_{\mathrm{B}}T}\right)+\gamma_{\mathrm{a}}\exp\left(\frac{z_i e_0\phi}{k_{\mathrm{B}}T}\right)-\gamma_{\mathrm{c}}-\gamma_{\mathrm{a}}\right)} \tag{S15}$$

where the bulk volume fraction of solvated ions is $v=2{a_{\mathrm{t}}}^3 n^{\mathrm{b}}$. The GCS model assumes $v=0$.

Combining Eq. (S1) and Eq. (S15), the BPB model is described as,

$$\nabla\left(\epsilon_{\mathrm{s}}^{\mathrm{b}}\nabla\phi\right)=\frac{2n_0^{\mathrm{b}}z_i e_0\sinh\left(\frac{\mp z_i e_0\phi}{k_{\mathrm{B}}T}\right)}{1+\frac{v}{2}\left(\gamma_{\mathrm{c}}\exp\left(\frac{-z_i e_0\phi}{k_{\mathrm{B}}T}\right)+\gamma_{\mathrm{a}}\exp\left(\frac{z_i e_0\phi}{k_{\mathrm{B}}T}\right)-\gamma_{\mathrm{c}}-\gamma_{\mathrm{a}}\right)} \tag{S16}$$

The dimensionless form is,

$$\frac{\partial^2 \boldsymbol{U}}{\partial \boldsymbol{X}^2}=\frac{\sinh\boldsymbol{U}}{1+\frac{\boldsymbol{v}}{2}(\boldsymbol{\gamma}_{\mathrm{c}}\boldsymbol{e}^{-\boldsymbol{U}}+\boldsymbol{\gamma}_{\mathrm{a}}\boldsymbol{e}^{\boldsymbol{U}}-\boldsymbol{\gamma}_{\mathrm{c}}-\boldsymbol{\gamma}_{\mathrm{a}})} \tag{S17}$$

And then the diffuse layer capacitance, C_{GC}, can be obtained from solving the modified PB equation considering the ion size effect,

$$\boldsymbol{C}_{\mathrm{GC}}=\frac{\partial\boldsymbol{\sigma}_{\mathrm{free}}}{\partial\boldsymbol{U}_{\mathrm{HP}}}=-\frac{\partial}{\partial\boldsymbol{U}_{\mathrm{HP}}}\left(\frac{\partial\boldsymbol{U}}{\partial\boldsymbol{X}}\right)_{\boldsymbol{X}=\mathrm{HP}^+} \tag{S18}$$

Suppl. Note 2. Force curve data analysis

In AFM measurements, the interaction force is determined from the cantilever deflection recorded by the position-sensitive photodetector (PSD). The deflection signal (in volts) is converted into the cantilever deformation using the calibrated deflection sensitivity[4], according to

$$deformation\ (nm) = deflection\ (V) \times deflection\ sensitivity\ (nm \cdot V^{-1})$$

The interaction force is then obtained from Hooke's law $(f = k\Delta z)$, where k is the spring constant of the cantilever.

$$Force\ (nN) = tip\ spring\ constant\ (nN \cdot nm^{-1}) \times deformation\ (nm)$$

The rupture force is defined as the maximum force observed immediately before the sudden drop in the force-separation curve.

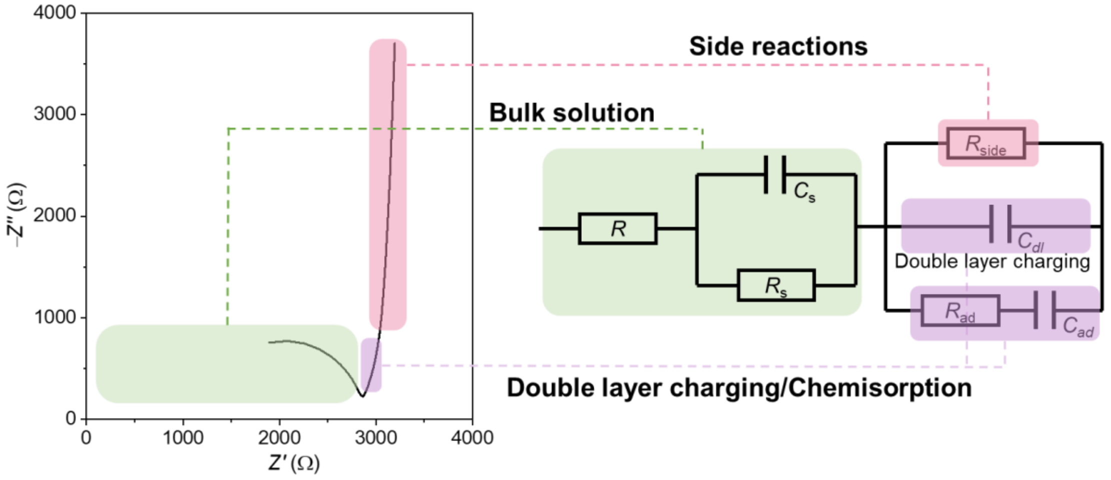

Supplementary Fig. 1. Typical Nyquist plot and the corresponding equivalent circuit model for electrolytes on HOPG electrode.

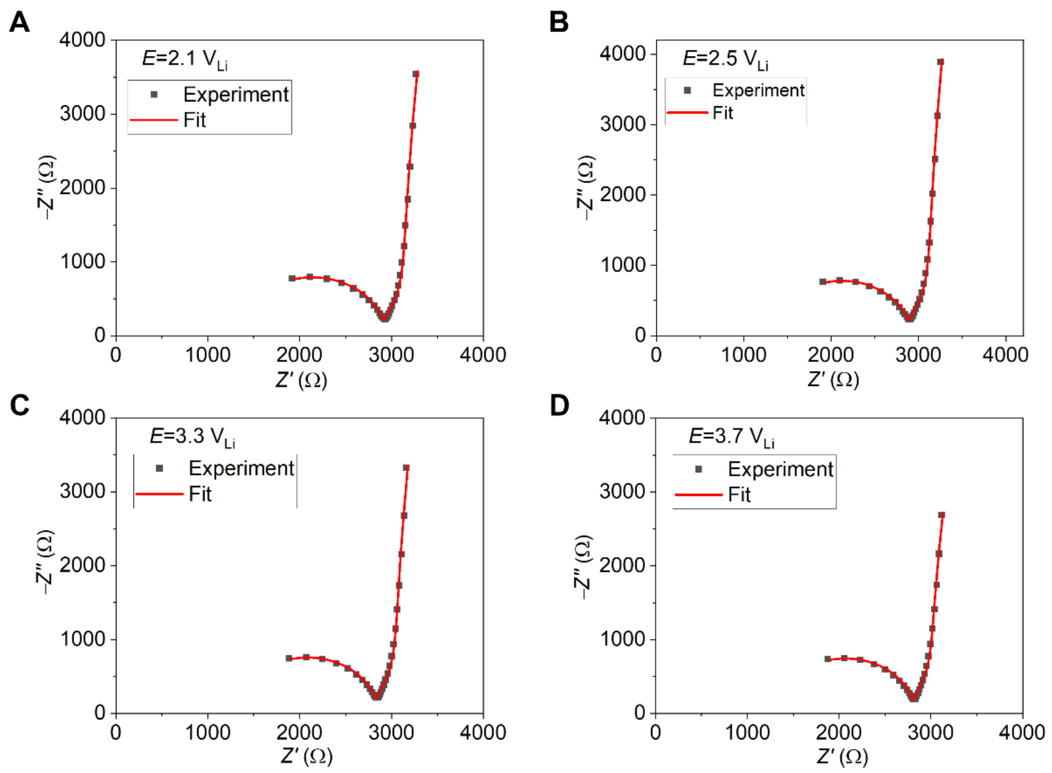

Supplementary Fig. 2. Comparison of fitted curves and experimental data for Li(G4)FSI on a HOPG electrode at different potentials. (**A**) E=2.1 V_{Li}; (**B**) E=2.5 V_{Li}; (**C**) E=3.3 V_{Li}; (**D**) E=3.7 V_{Li}.

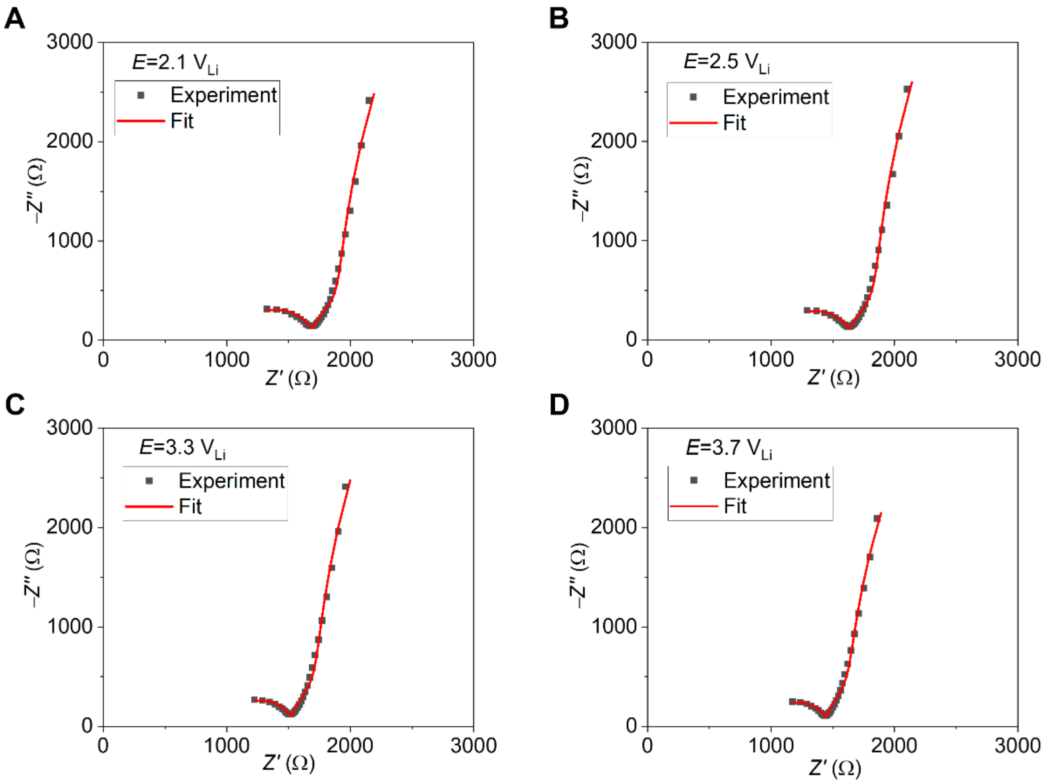

Supplementary Fig. 3. Comparison of fitted curves and experimental data for Li(G4)$_3$FSI on a HOPG electrode at different potentials. (**A**) E=2.1 V_{Li}; (**B**) E=2.5 V_{Li}; (**C**) E=3.3 V_{Li}; (**D**) E=3.7 V_{Li}.

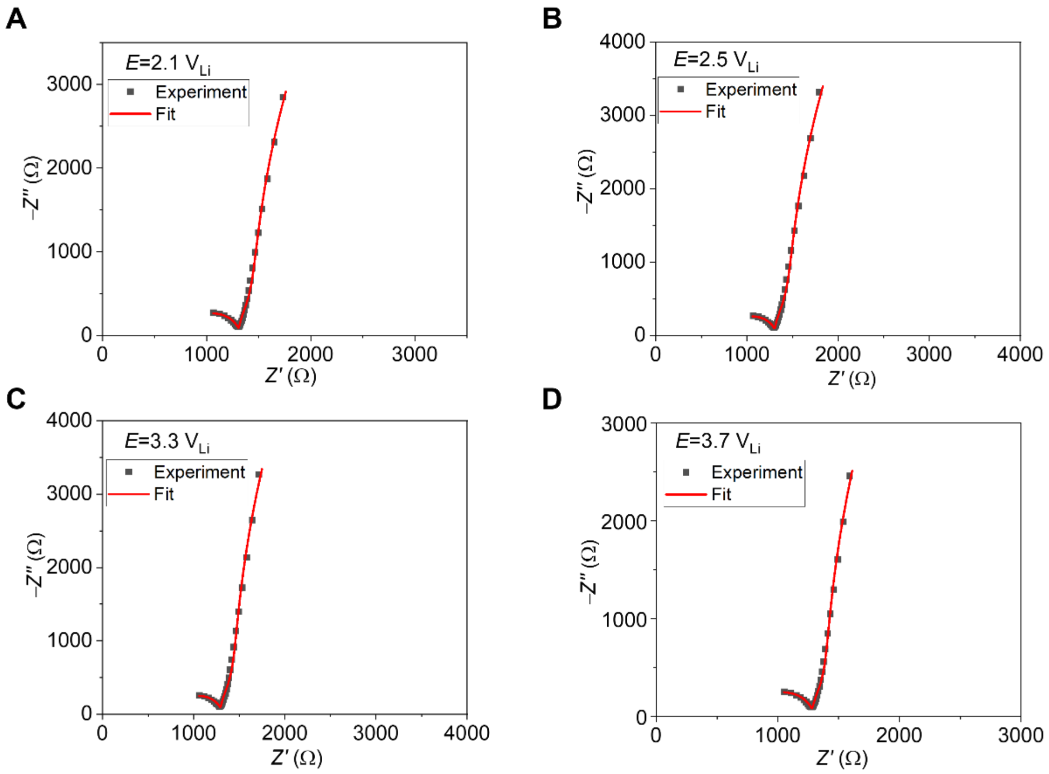

Supplementary Fig. 4. Comparison of fitted curves and experimental data for Li(G4)$_5$FSI on a HOPG electrode at different potentials. (**A**) E=2.1 V$_{Li}$; (**B**) E=2.5 V$_{Li}$; (**C**) E=3.3 V$_{Li}$; (**D**) E=3.7 V$_{Li}$.

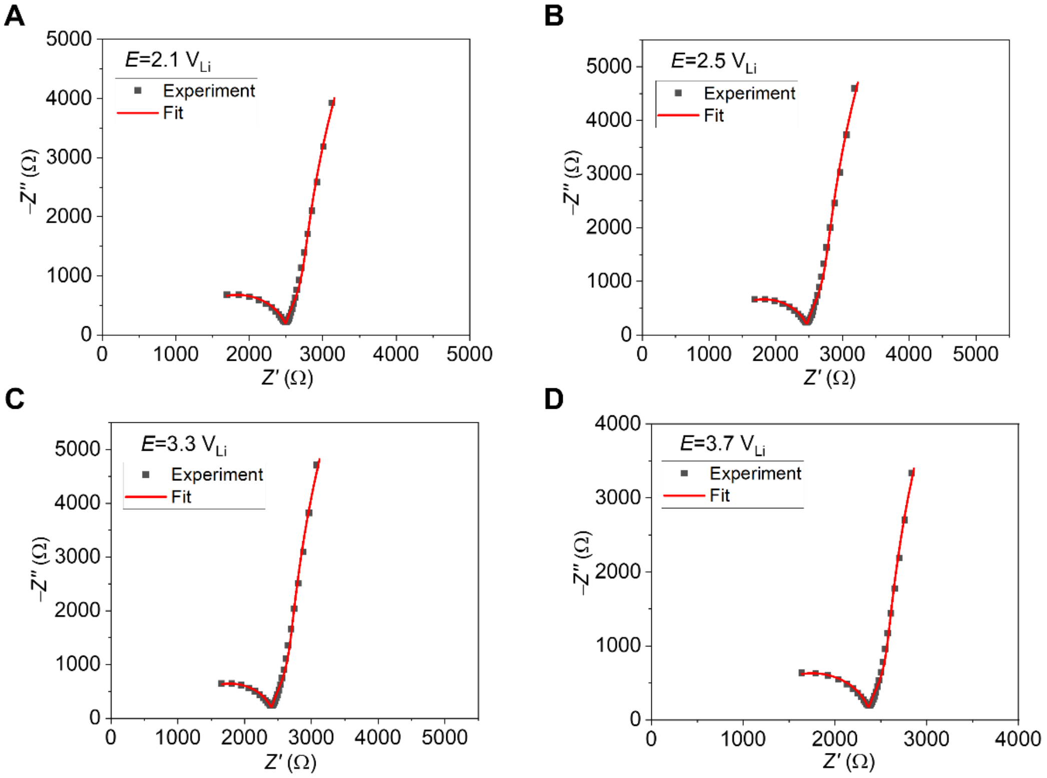

Supplementary Fig. 5. Comparison of fitted curves and experimental data for $Li(G4)_{10}FSI$ on a HOPG electrode at different potentials. (**A**) E=2.1 V_{Li}; (**B**) E=2.5 V_{Li}; (**C**) E=3.3 V_{Li}; (**D**) E=3.7 V_{Li}.

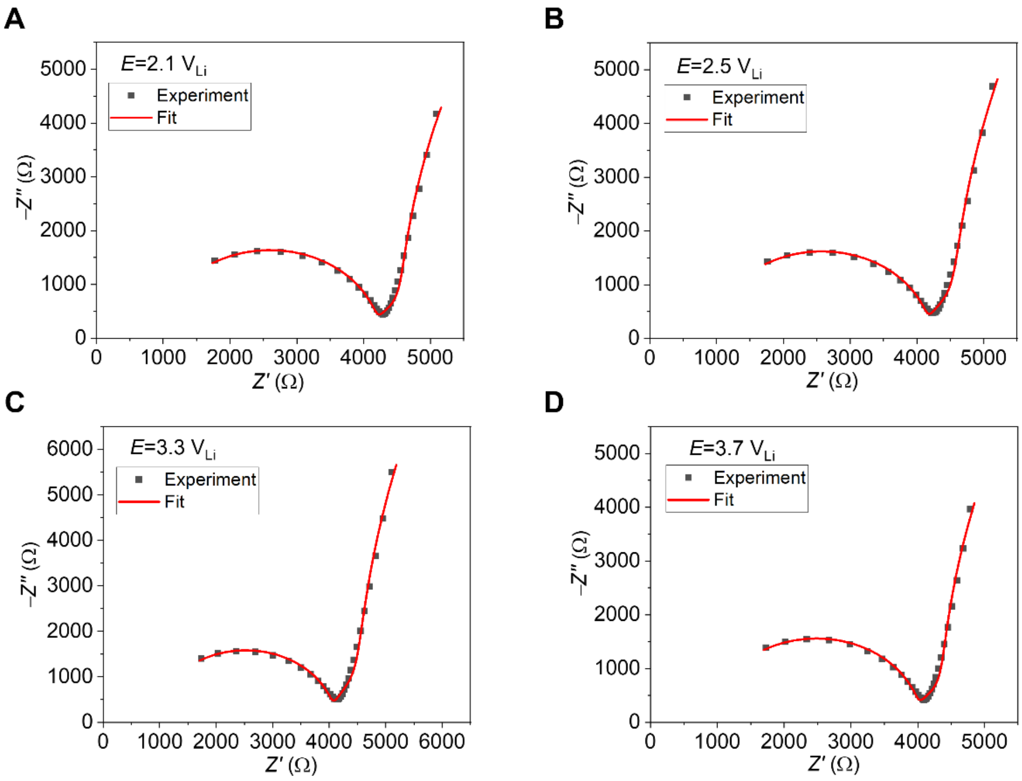

Supplementary Fig. 6. Comparison of fitted curves and experimental data for $Li(G4)_{20}FSI$ on a HOPG electrode at different potentials. (**A**) E=2.1 V_{Li}; (**B**) E=2.5 V_{Li}; (**C**) E=3.3 V_{Li}; (**D**) E=3.7 V_{Li}.

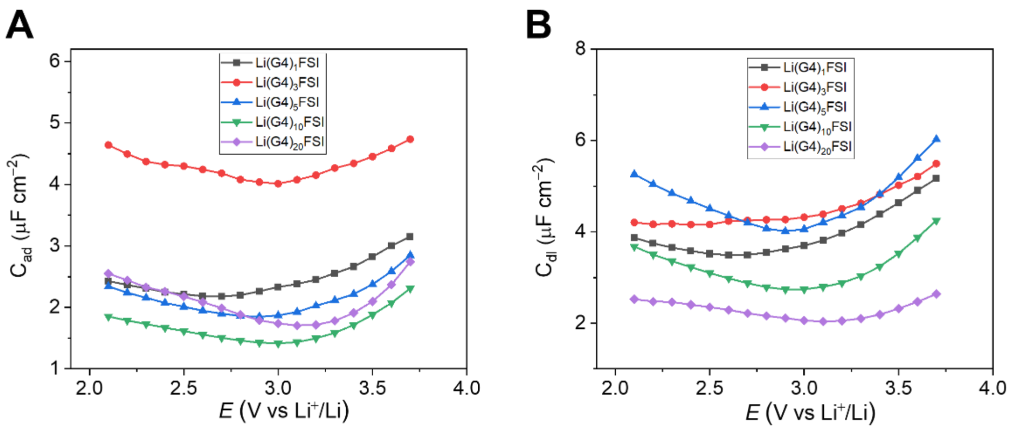

Supplementary Fig. 7. C_{ad}-E (**A**) and C_{dl}-E (**B**) curves of HOPG in Li(G4)$_x$FSI

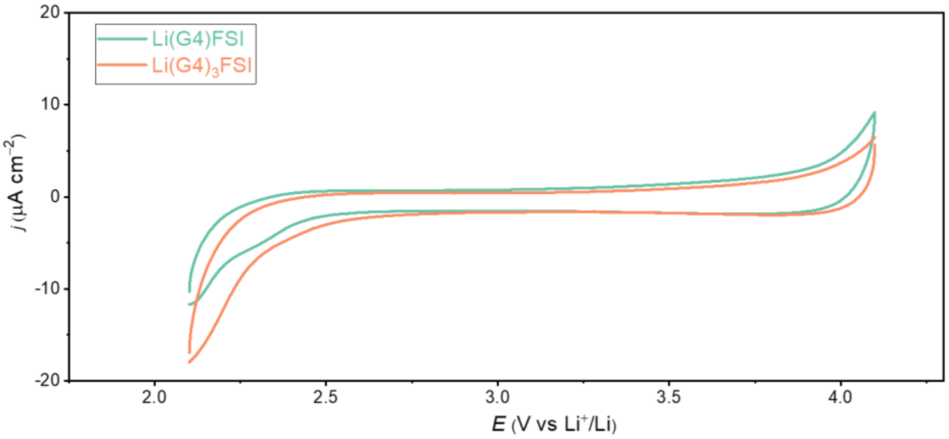

Supplementary Fig. 8. Cyclic Voltammograms of Li(G4)FSI and Li(G4)$_3$FSI on HOPG electrode. Scan rate: 10 mV s^{-1}.

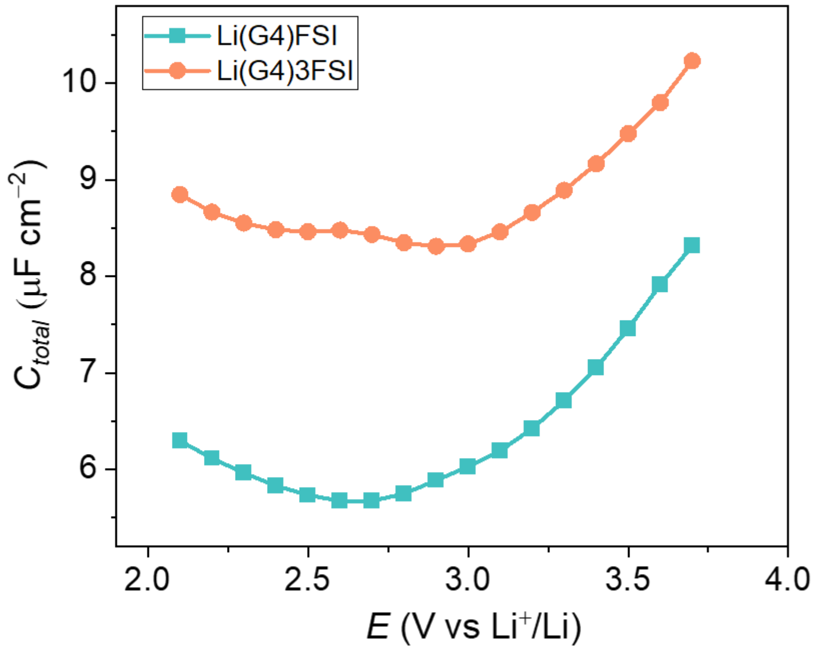

Supplementary Fig. 9. C_{tot}-E curves of HOPG in Li(G4)FSI and $\mathrm{Li(G4)_3FSI}$.

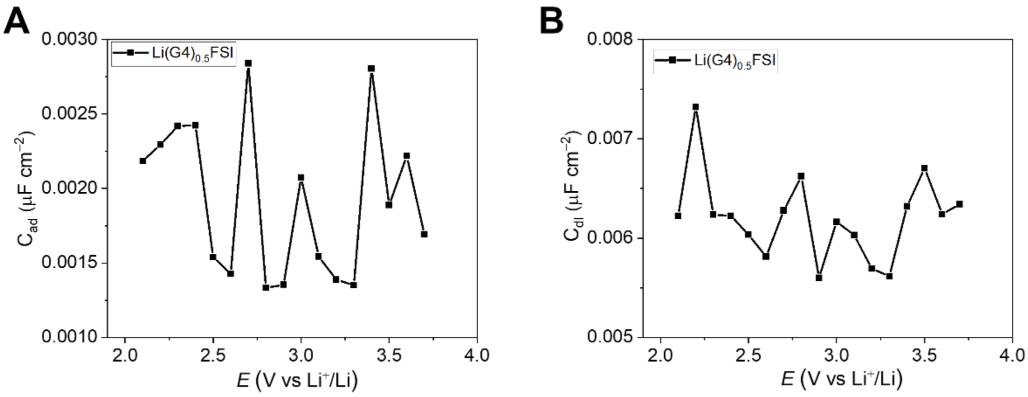

Supplementary Fig. 10. C_{ad}-E (**A**) and C_{dl}-E (**B**) curves of HOPG in $Li(G4)_{0.5}FSI$

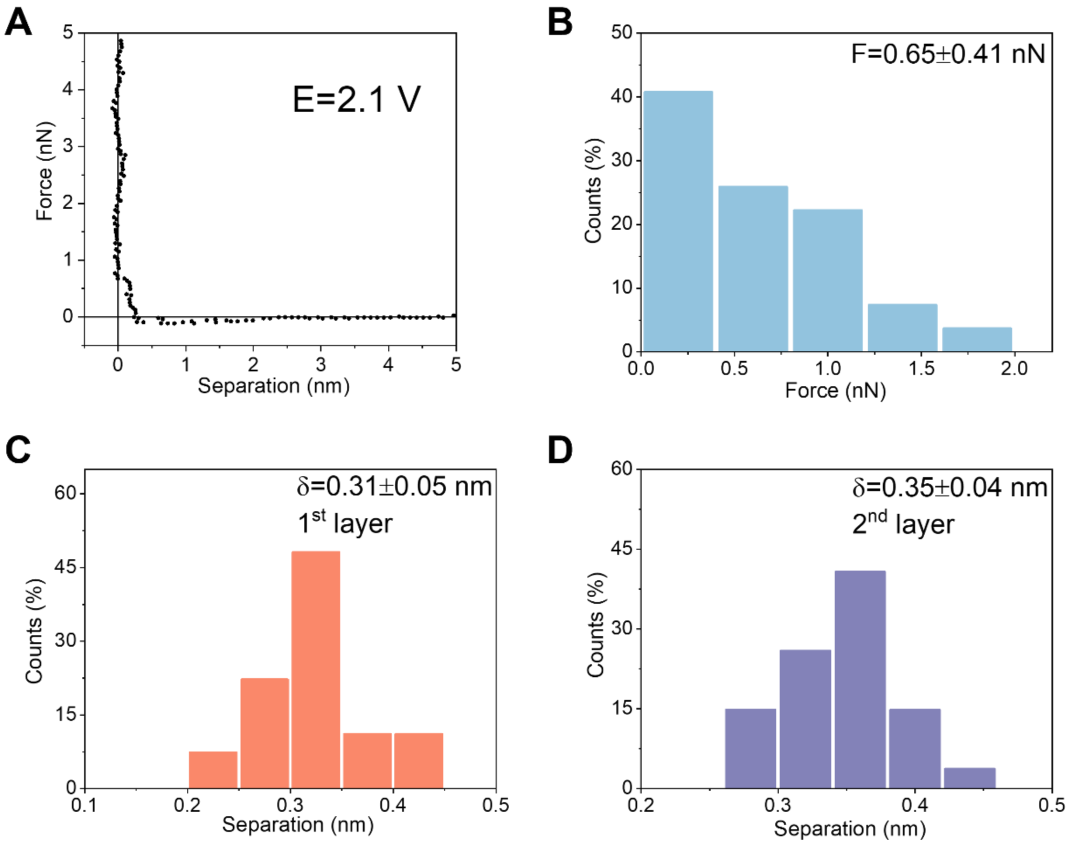

Supplementary Fig. 11. In situ AFM characterization of Li(G4)$_3$FSI when E=2.1 V$_{Li}$. (**A**) Individual typical AFM force curve. (**B**) Two-dimensional histogram of the rupture force. (**C**) and (**D**) Two-dimensional histograms of the first layer thickness and the second layer thickness.

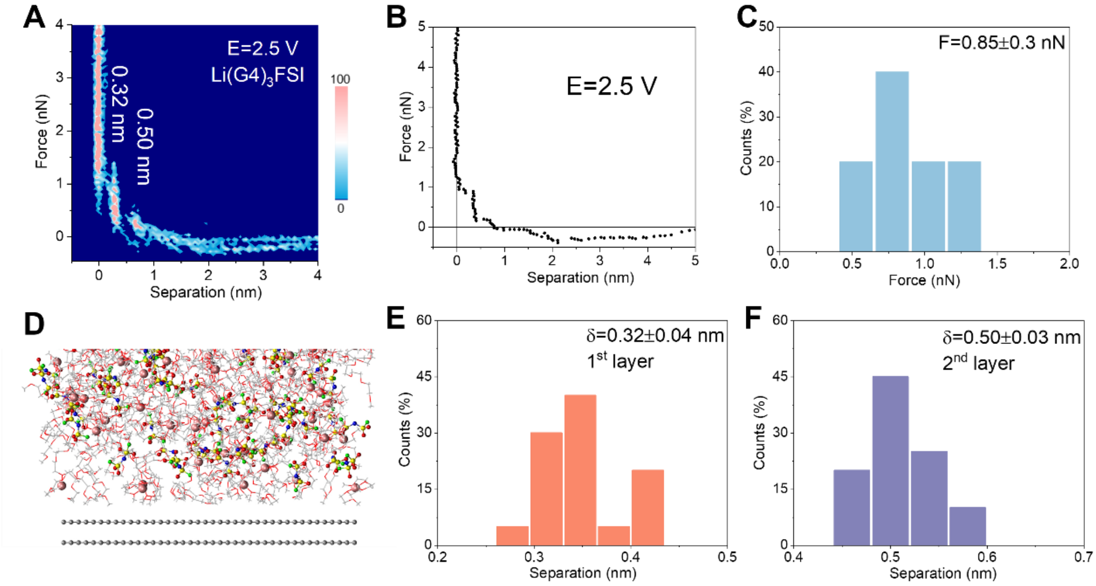

Supplementary Fig. 12. In situ AFM characterization of Li(G4)$_3$FSI when E=2.5 V_{Li}. (**A**) Two-dimensional AFM force-separation histograms from 20 independent force curves with probability colour bar on the right-hand side. (**B**) Individual typical AFM force curve. (**C**) Two-dimensional histogram of the rupture force. (**D**) Representative snapshots from the simulation. (**E**) and (**F**) Two-dimensional histograms of the first layer thickness and the second layer thickness.

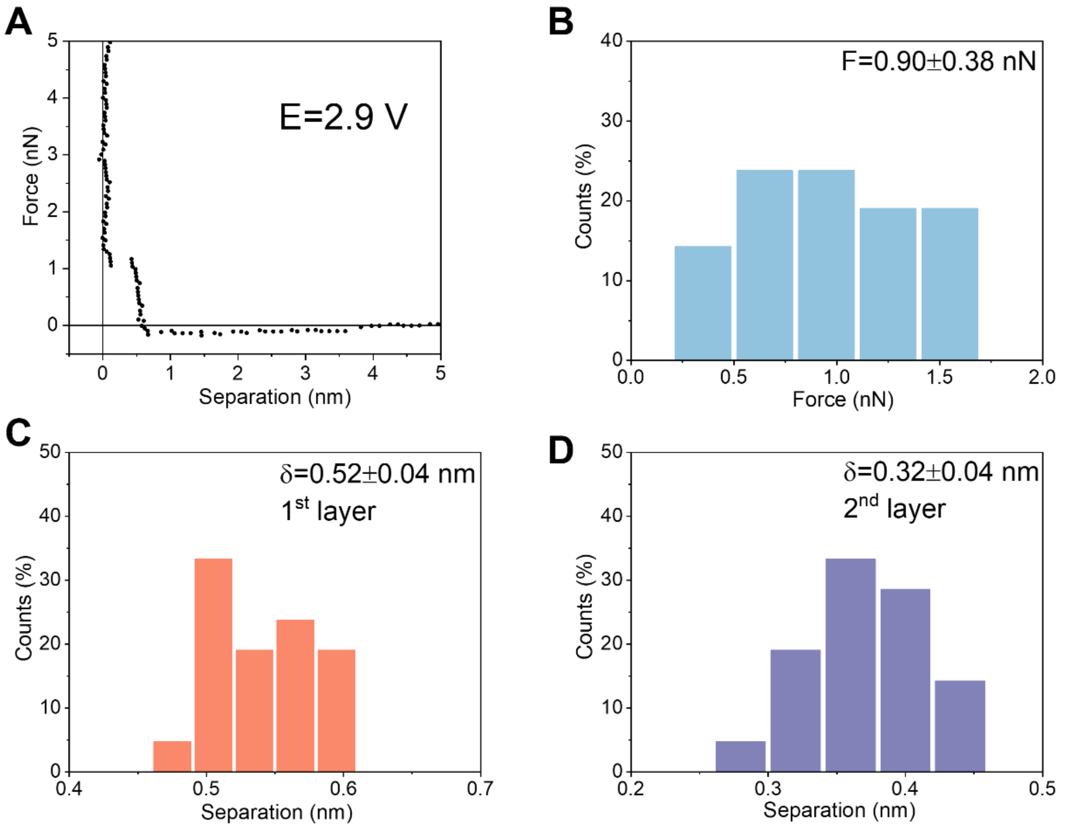

Supplementary Fig. 13. In situ AFM characterization of Li(G4)$_3$FSI when E=2.9 V$_{Li}$. (**A**) Individual typical AFM force curve. (**B**) Two-dimensional histogram of the rupture force. (**C**) and (**D**) Two-dimensional histograms of the first layer thickness and the second layer thickness.

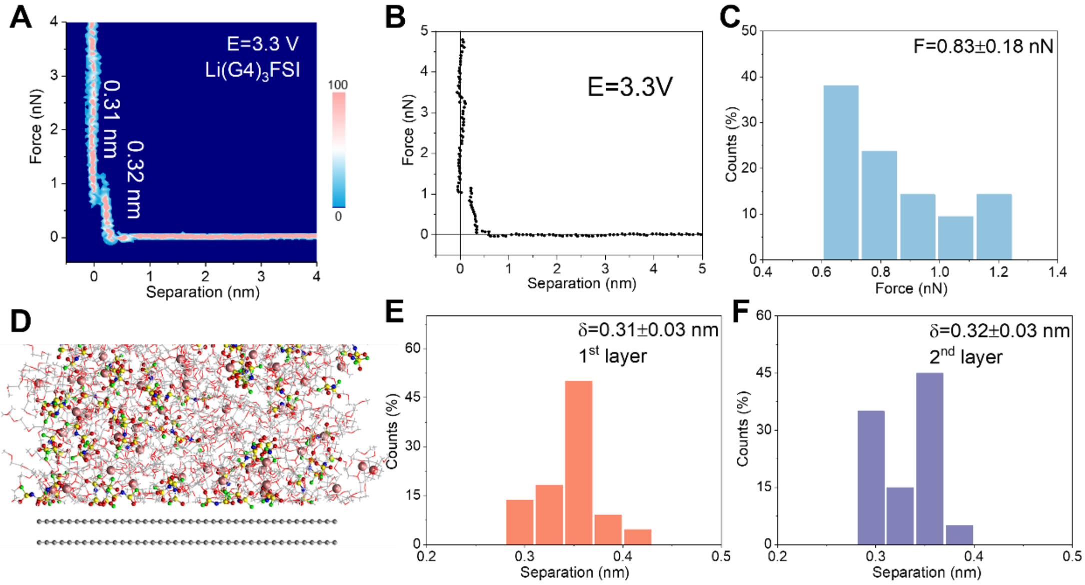

Supplementary Fig. 14. In situ AFM characterization of Li(G4)$_3$FSI when E=3.3 V$_{Li}$. (**A**) Two-dimensional AFM force-separation histograms from 20 independent force curves with probability colour bar on the right-hand side. (**B**) Individual typical AFM force curve. (**C**) Two-dimensional histogram of the rupture force. (**D**) Representative snapshots from the simulation. (**E**) and (**F**) Two-dimensional histograms of the first layer thickness and the second layer thickness.

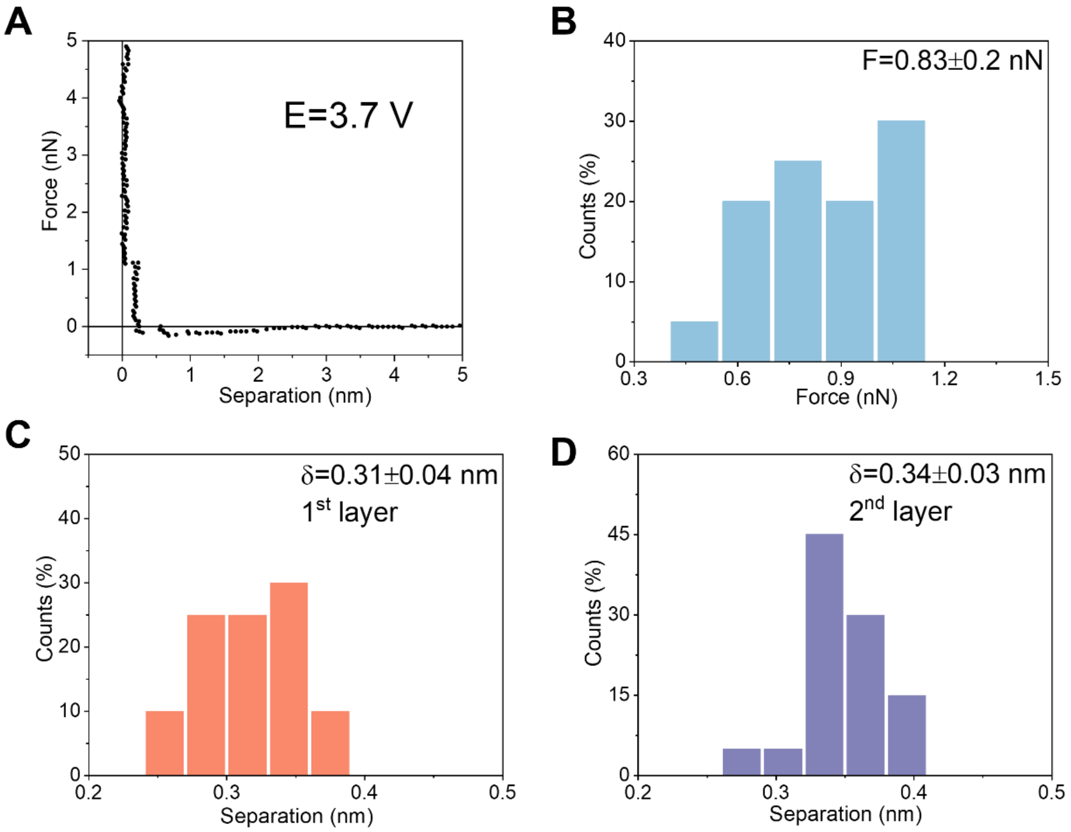

Supplementary Fig. 15. In situ AFM characterization of Li(G4)$_3$FSI when E=3.7 V_{Li}. (**A**) Individual typical AFM force curve. (**B**) Two-dimensional histogram of the rupture force. (**C**) and (**D**) Two-dimensional histograms of the first layer thickness and the second layer thickness.

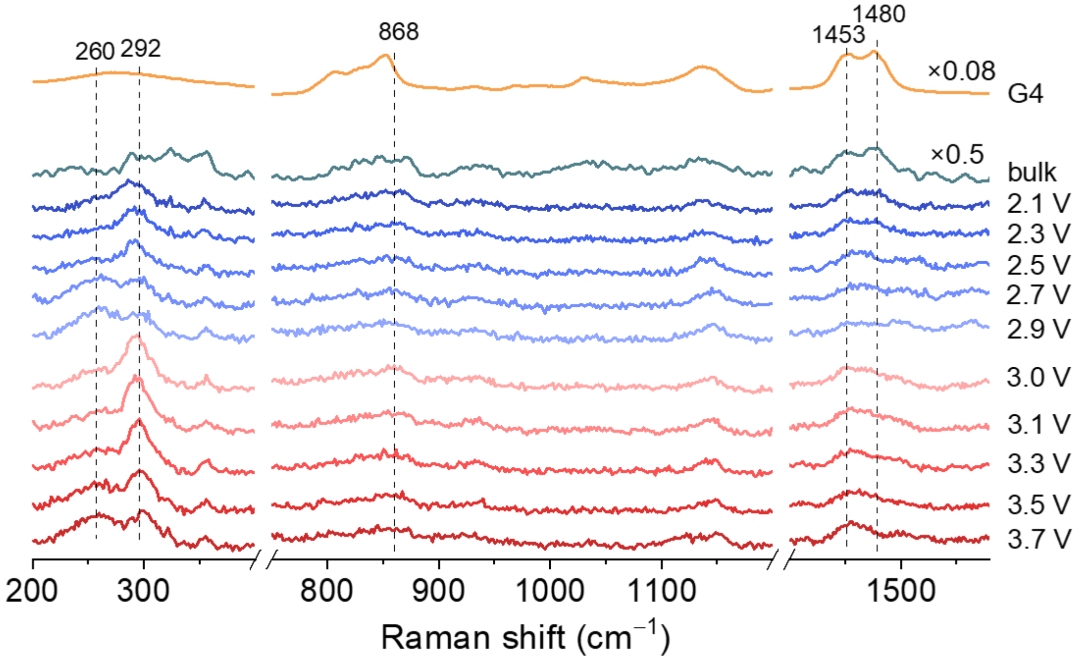

Supplementary Fig. 16. In situ Raman spectra of Li(G4)$_3$FSI. The blue Raman spectra correspond to the potentials being negative relative to PZC, while the red Raman spectra correspond to the potentials being positive relative to PZC.

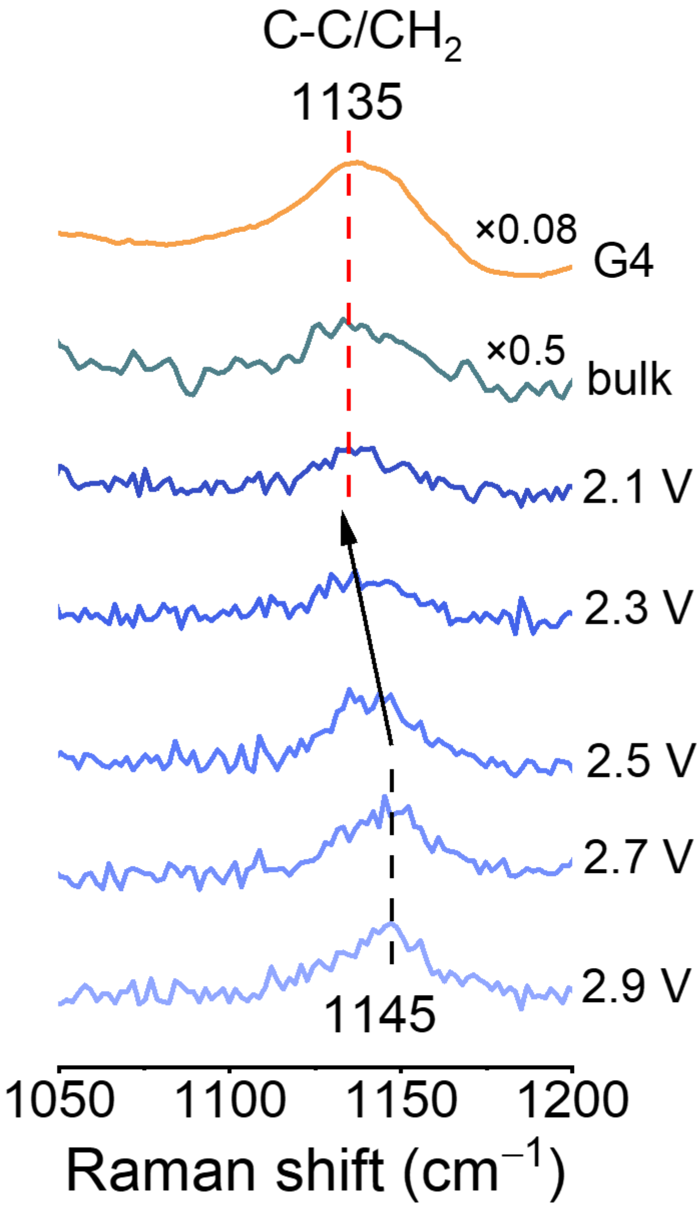

Supplementary Fig. 17. The spectra of the coupled C-C stretching and CH_2 wagging modes as the potential shifts toward more negative values.

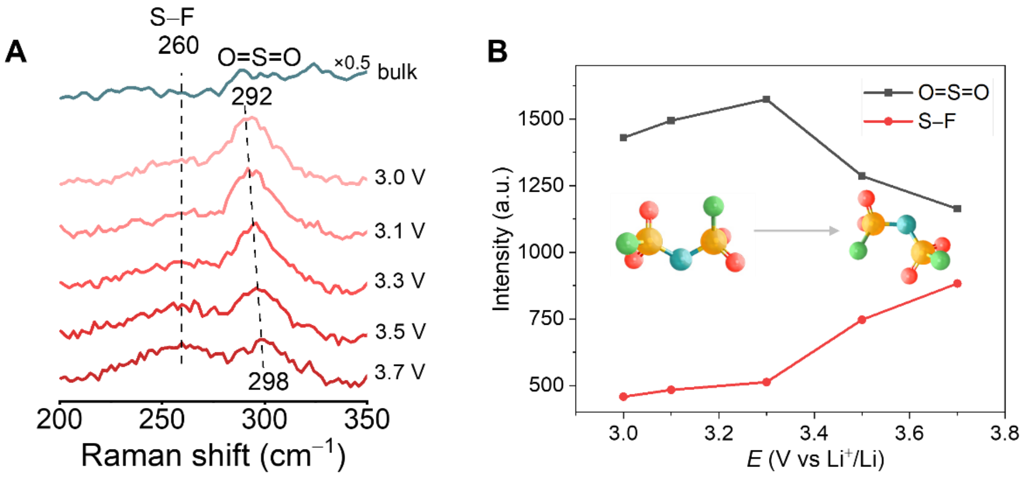

Supplementary Fig. 18. (**A**) Raman spectra of the S-F and SO_2 rocking modes under positive polarization. (**B**) Potential-dependent peak intensities of S-F and SO_2 modes. F, S, O, and N atoms are shown in green, yellow, red, and cyan, respectively.

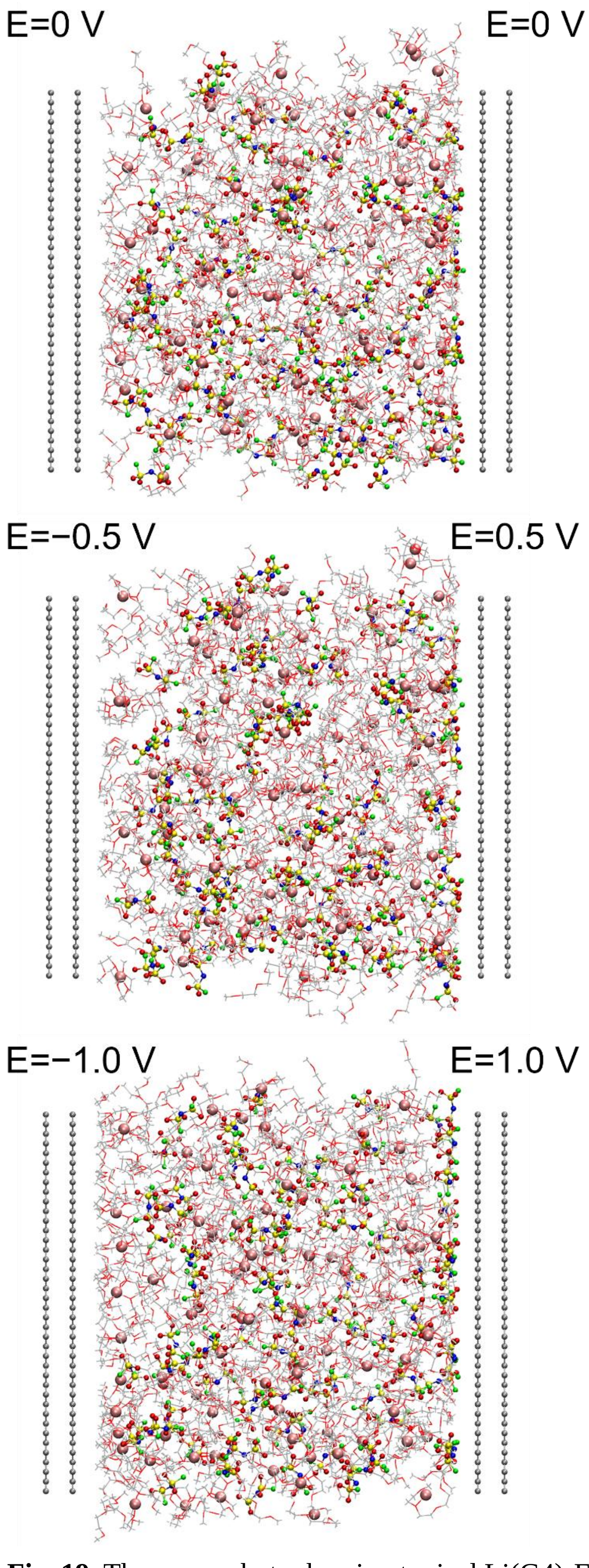

Supplementary Fig. 19. Three snapshots showing typical Li(G4)$_3$FSI in the innermost layer at +1.0 V, 0 V and −1.0 V relative to the PZC.

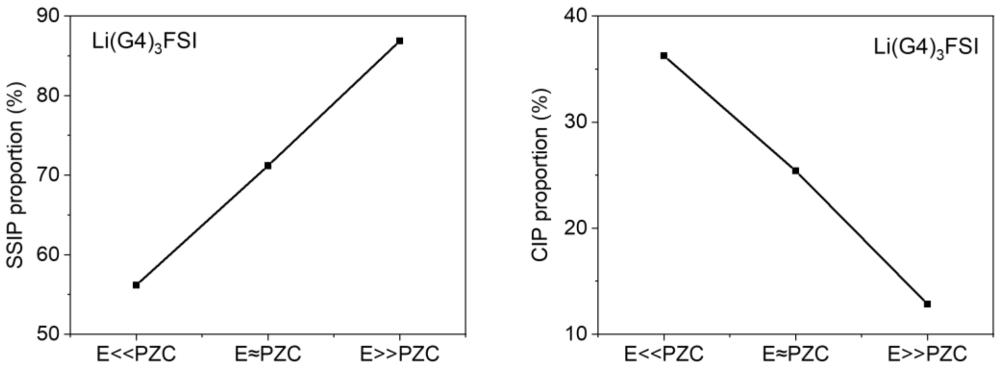

Supplementary Fig. 20. Potential-Dependent Evolution of Simulated SSIP and CIP Fractions in $Li(G4)_3FSI$.

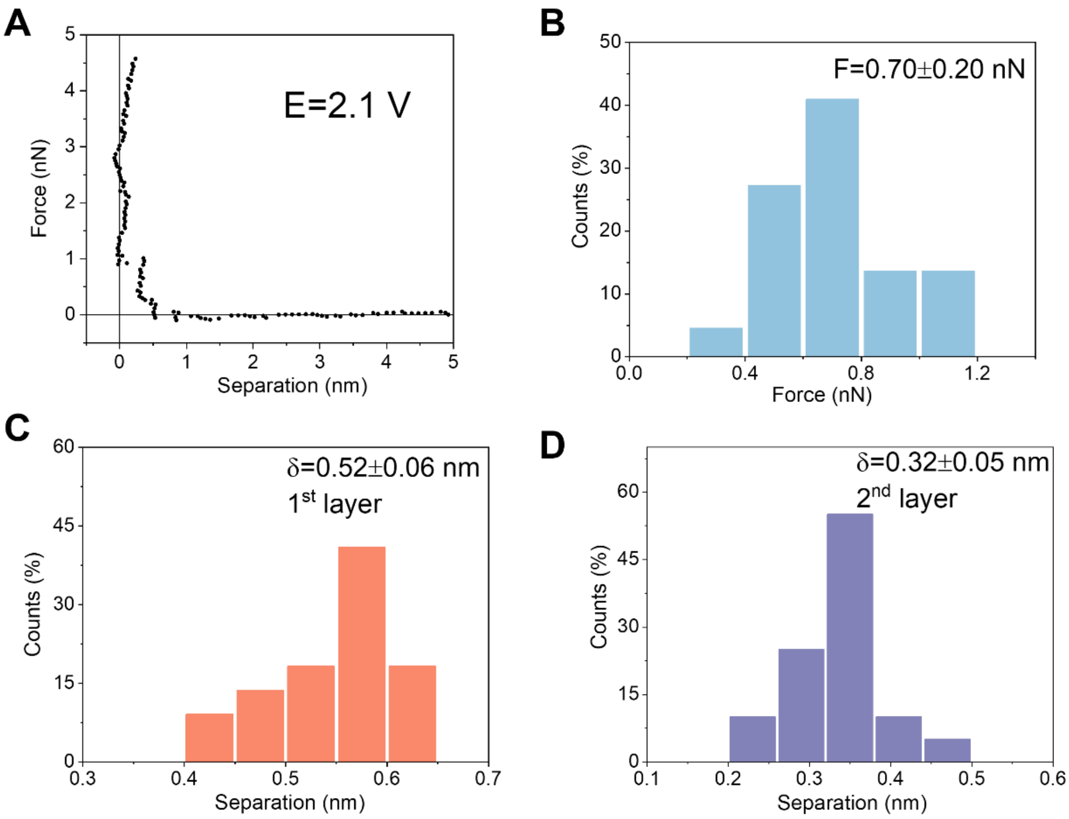

Supplementary Fig. 21. In situ AFM characterization of Li(G4)FSI when E=2.1 V_{Li}. (**A**) Individual typical AFM force curve. (**B**) Two-dimensional histogram of the rupture force. (**C**) and (**D**) Two-dimensional histograms of the first layer thickness and the second layer thickness.

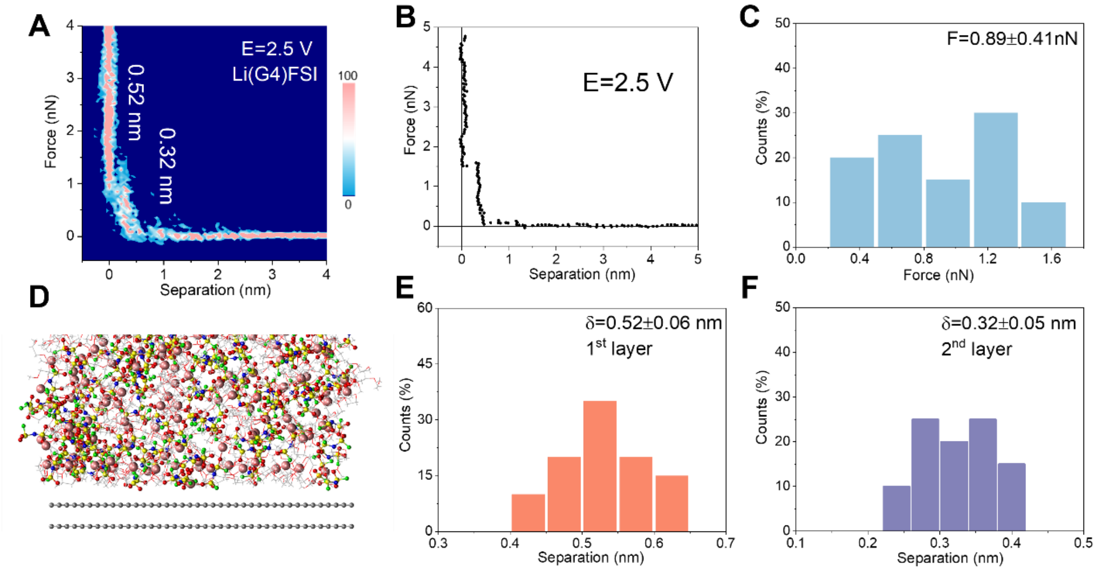

Supplementary Fig. 22. In situ AFM characterization of Li(G4)FSI when E=2.5 V_{Li}. (**A**) Two-dimensional AFM force-separation histograms from 20 independent force curves with probability colour bar on the right-hand side. (**B**) Individual typical AFM force curve. (**C**) Two-dimensional histogram of the rupture force. (**D**) Representative snapshots from the simulation. (**E**) and (**F**) Two-dimensional histograms of the first layer thickness and the second layer thickness.

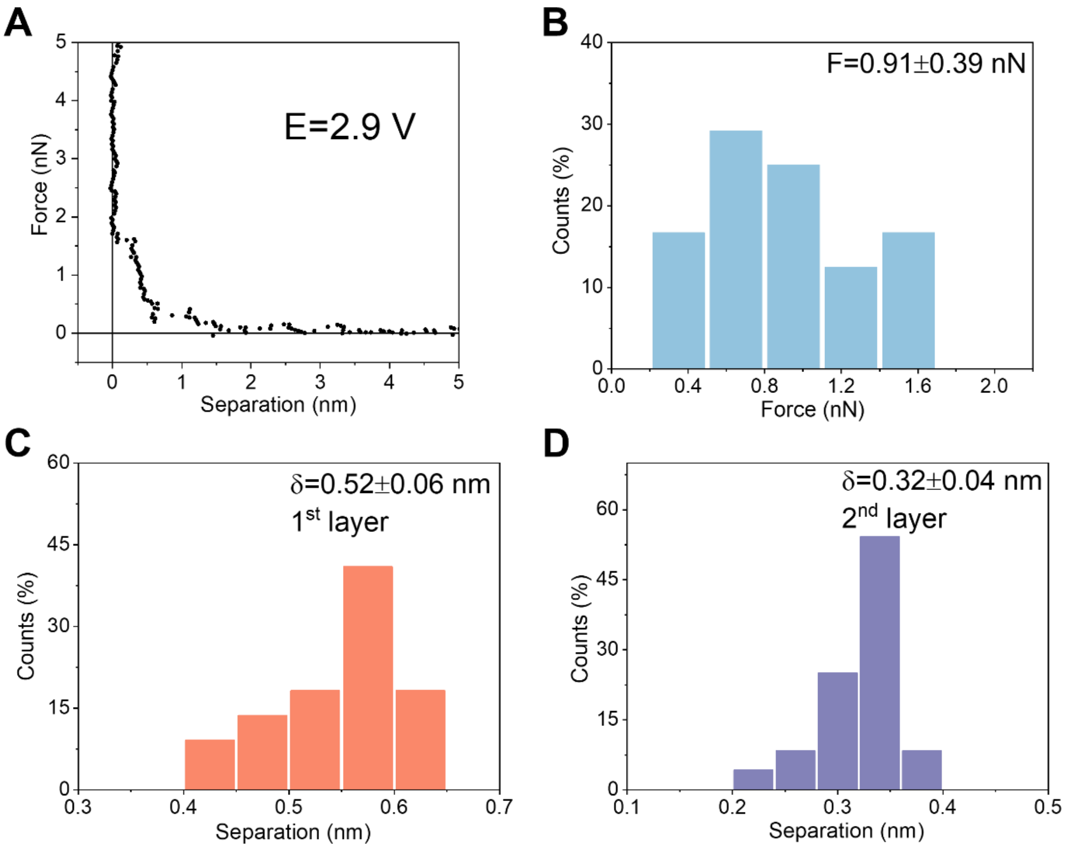

Supplementary Fig. 23. In situ AFM characterization of Li(G4)FSI when E=2.9 V_{Li}. (**A**) Individual typical AFM force curve. (**B**) Two-dimensional histogram of the rupture force. (**C**) and (**D**) Two-dimensional histograms of the first layer thickness and the second layer thickness.

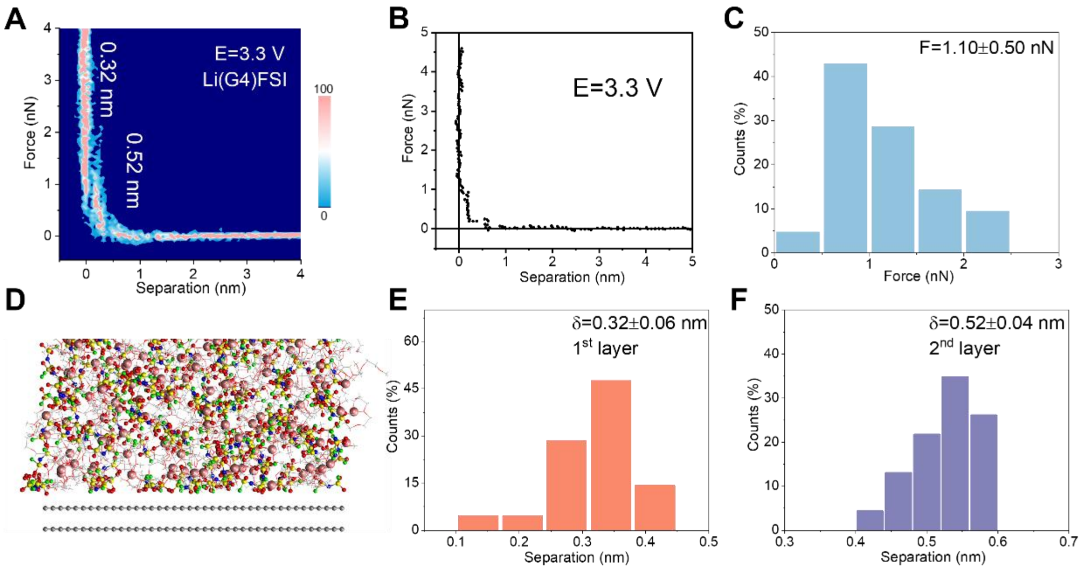

Supplementary Fig. 24. In situ AFM characterization of Li(G4)FSI when E=3.3 V_{Li}. (**A**) Two-dimensional AFM force-separation histograms from 20 independent force curves with probability colour bar on the right-hand side. (**B**) Individual typical AFM force curve. (**C**) Two-dimensional histogram of the rupture force. (**D**) Representative snapshots from the simulation. (**E**) and (**F**) Two-dimensional histograms of the first layer thickness and the second layer thickness.

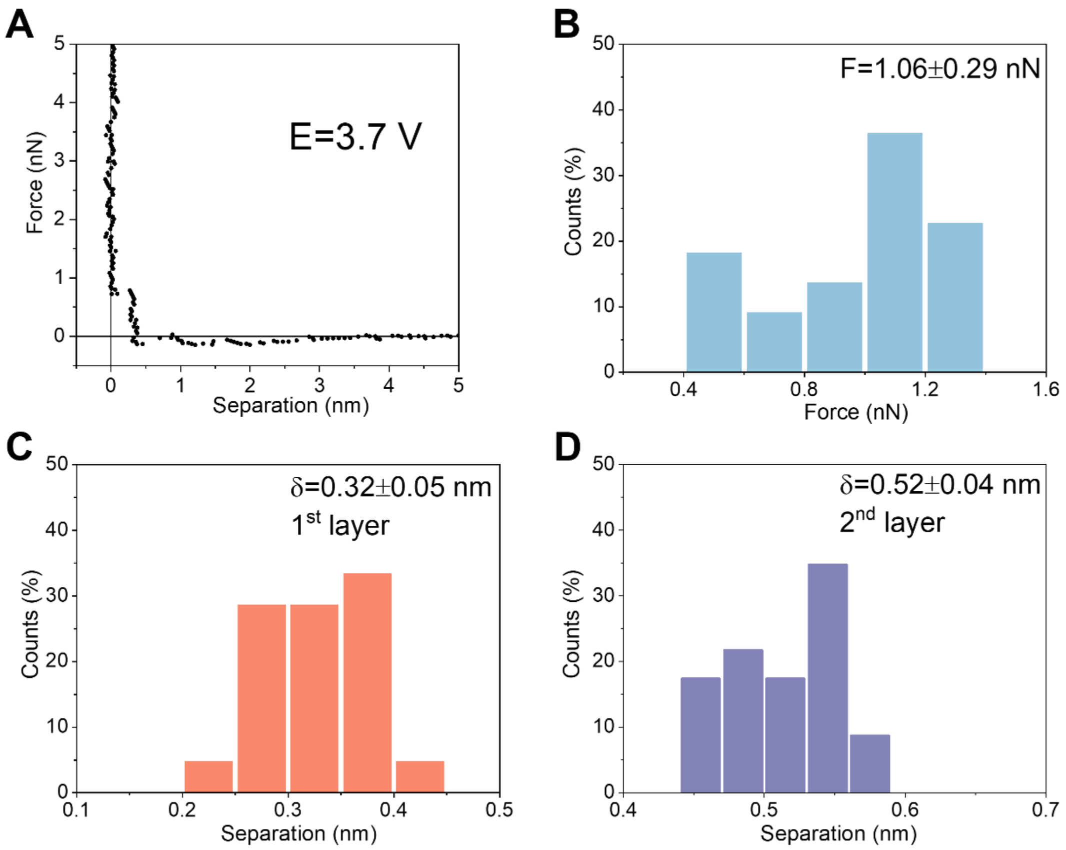

Supplementary Fig. 25. In situ AFM characterization of Li(G4)FSI when E=3.7 V_{Li}. (**A**) Individual typical AFM force curve. (**B**) Two-dimensional histogram of the rupture force. (**C**) and (**D**) Two-dimensional histograms of the first layer thickness and the second layer thickness.

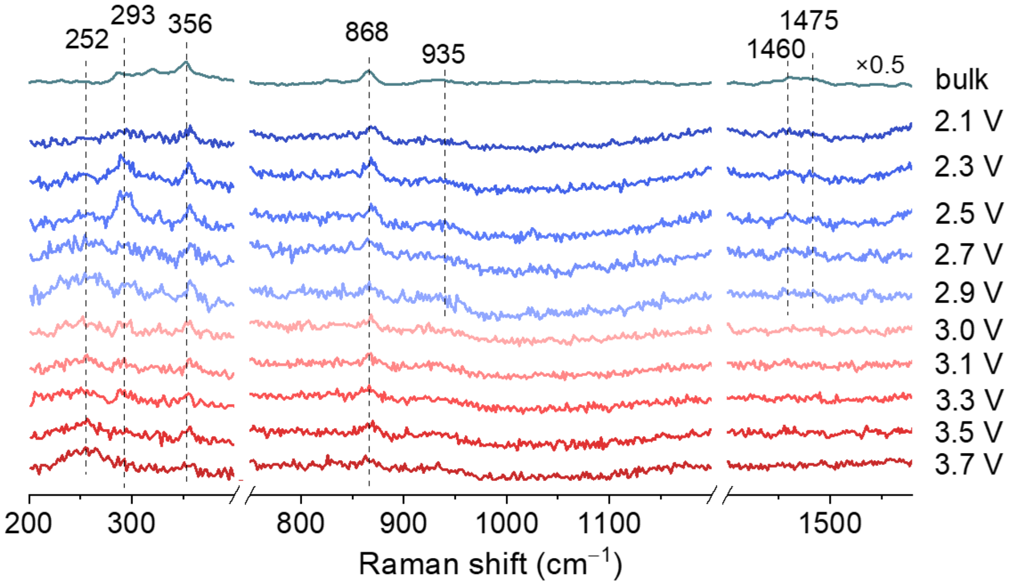

Supplementary Fig. 26. In situ Raman spectra of Li(G4)FSI. The blue Raman spectra correspond to the potentials being negative relative to PZC, while the red Raman spectra correspond to the potentials being positive relative to PZC.

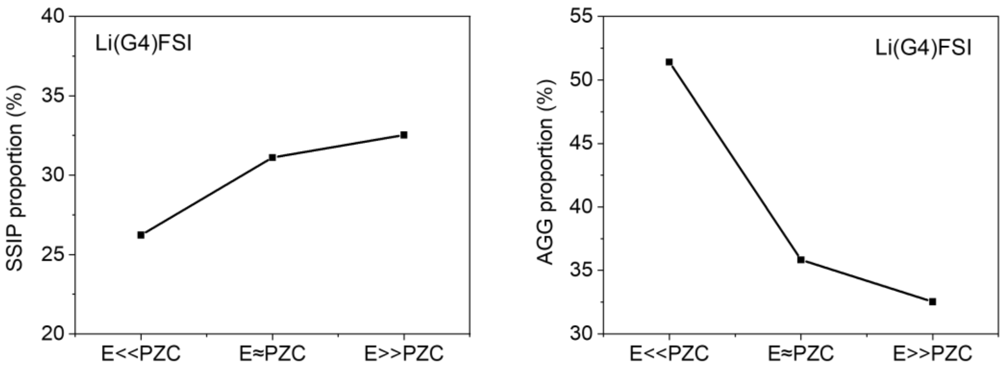

Supplementary Fig. 27. Potential-Dependent Evolution of Simulated SSIP and AGG Fractions in Li(G4)FSI.

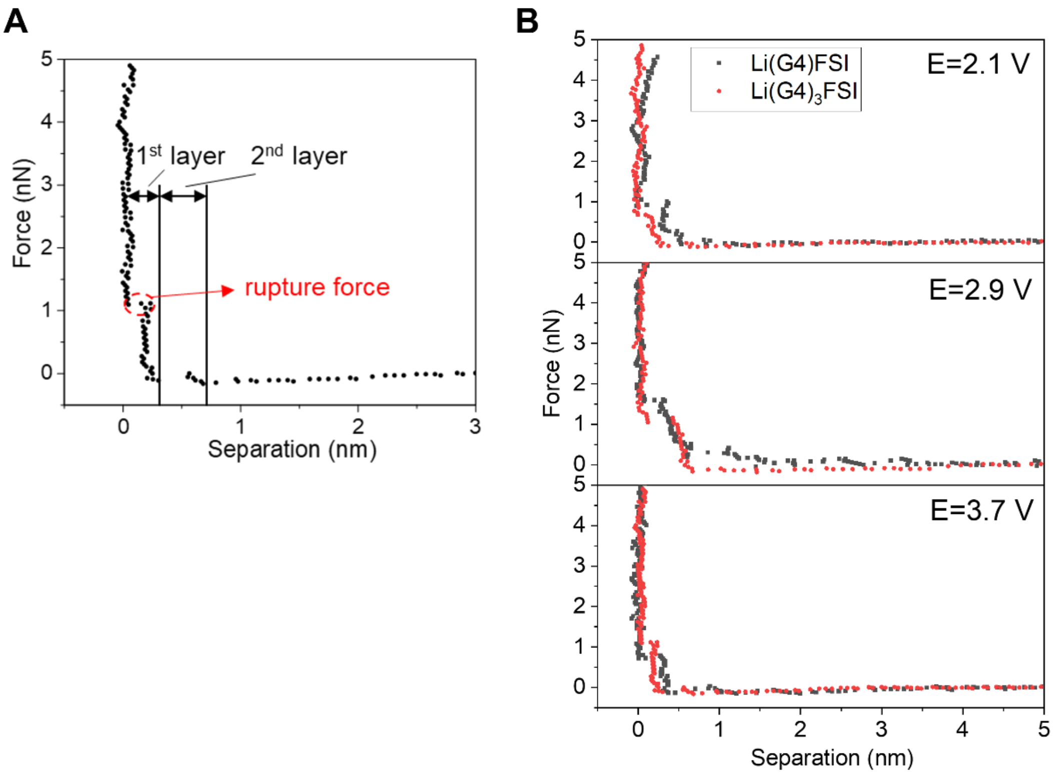

Supplementary Fig. 28. (**A**), Example of a representative force-separation curve. (**B**), Force curves at different potentials for two electrolyte concentrations (E < PZC, E ≈ PZC, and E > PZC). Black: Li(G4)FSI, Red: $Li(G4)_3FSI$.

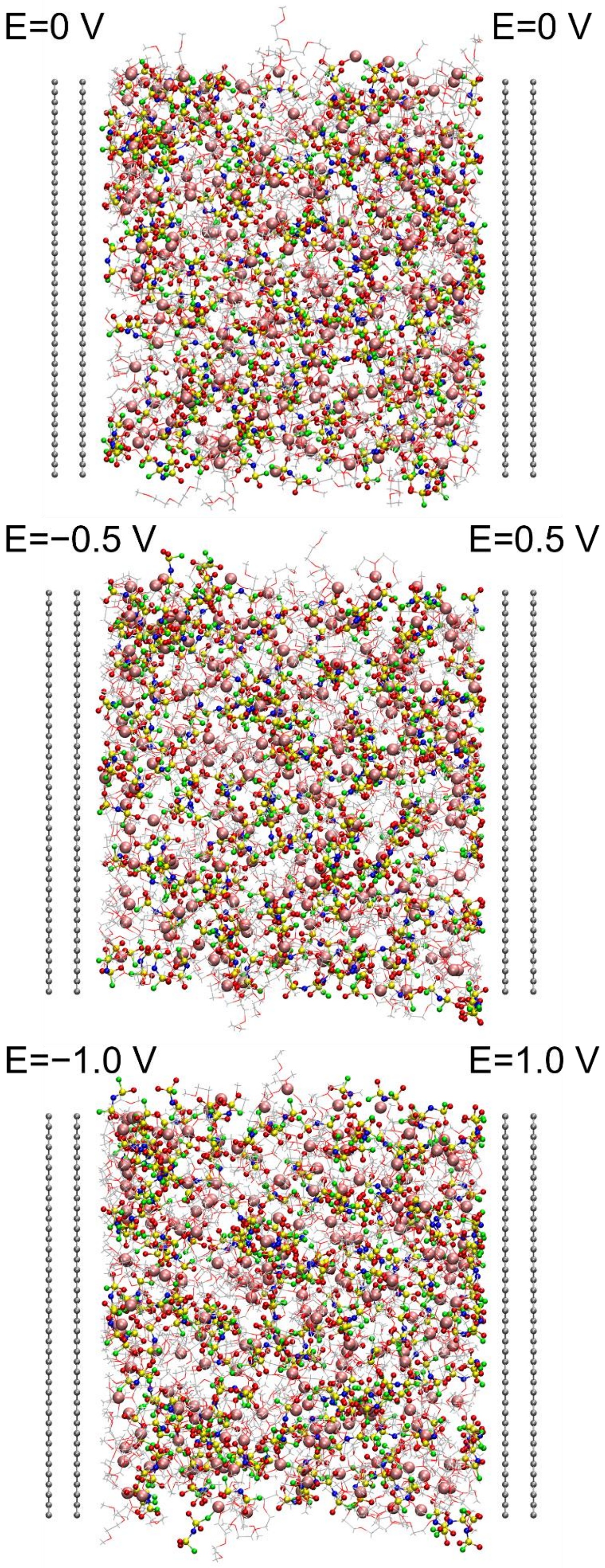

Supplementary Fig. 29. Three snapshots showing typical Li(G4)FSI in the innermost layer at +1.0 V, 0 V and −1.0 V relative to the PZC

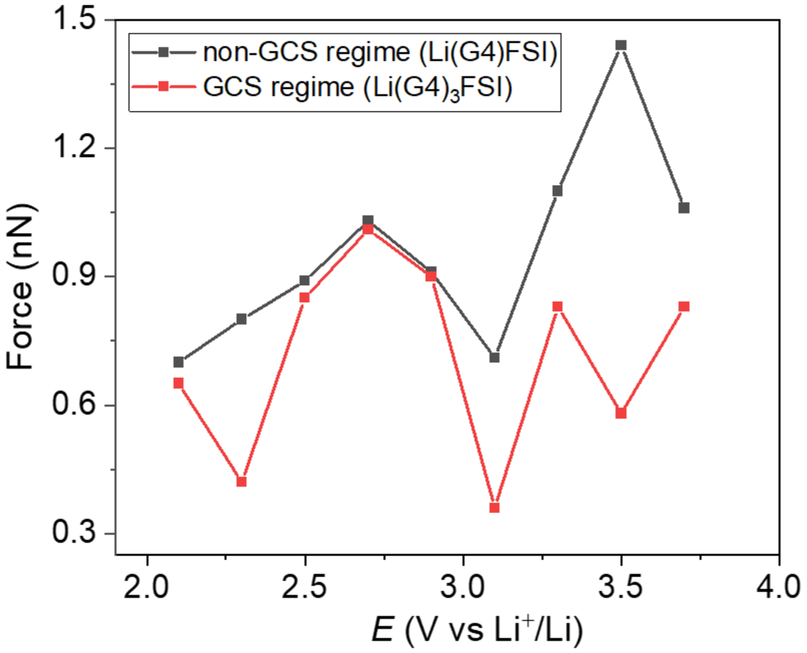

Supplementary Fig. 30. The average rupture force of Li(G4)FSI and $Li(G4)_3FSI$ under different electrode potentials.

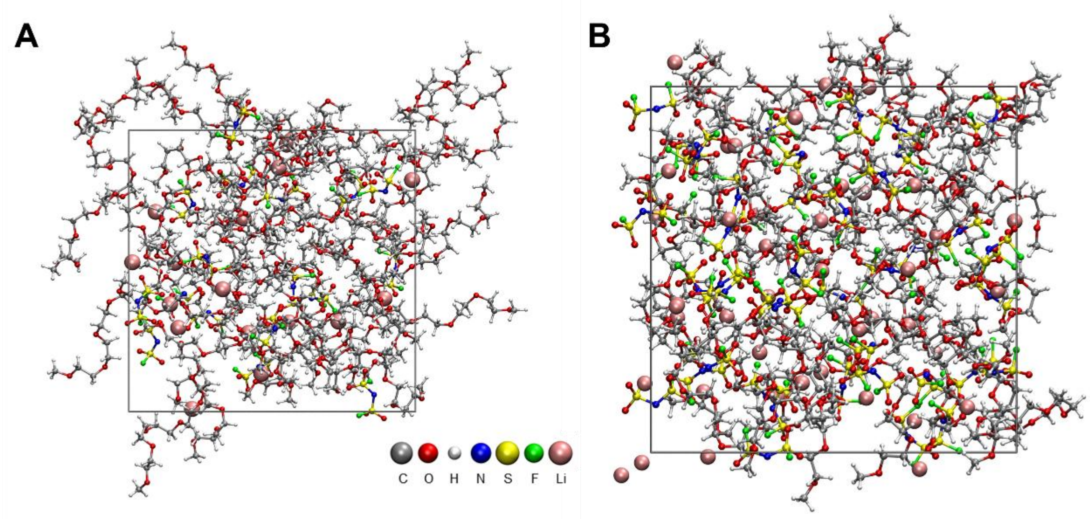

Supplementary Fig. 31. 3D snapshot of Li(G4)FSI (**A**) and Li$(G4)_3$FSI (**B**) electrolytes obtained from MLMD simulations.

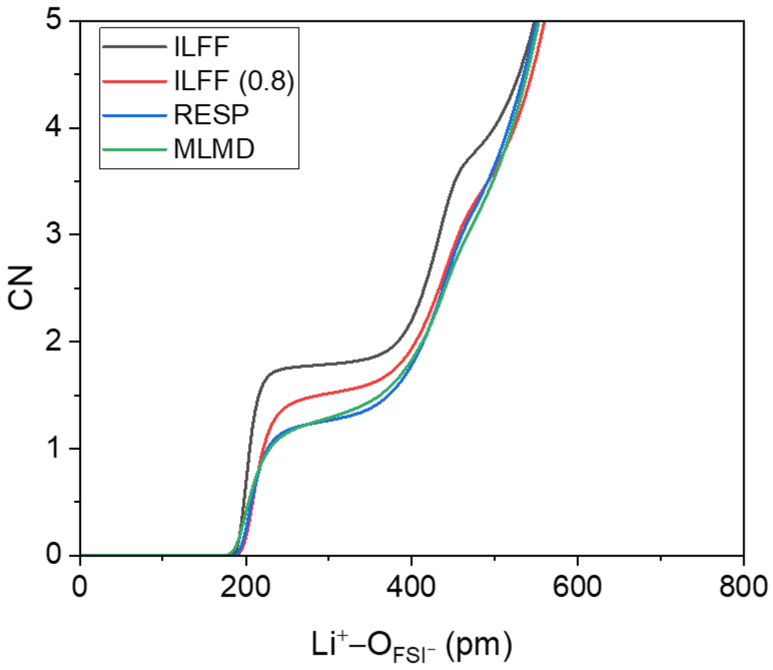

Supplementary Fig. 32. Coordination number (CN) of Li^+–O_{FSI^-} pairs in Li(G4)FSI electrolyte obtained from CMD simulations with different force fields.

Supplementary Table 1. Basic model parameters

Symbol	Definition
C_{H}	the inner layer or Helmholtz capacitance
C_{GC}	the diffuse layer capacitance
C_{ad}	the adsorption capacitance
C_{dl}	the double-layer capacitance
C_{tot}	the total capacitance
E	potential
$\epsilon_{\mathrm{s}}^{\mathrm{b}}$	the bulk dielectric permittivity of the electrolyte solution
ϕ	the electric potential
z_i	the charge number of ion i
F	the Faraday's constant
c_i	the concentration of ion i
c_i^{b}	the concentration of ion i in the solution bulk
R	the gas constant
T	the temperature
c^b	the concentration of total anions (cations) in the solution bulk
ϵ_{HP}	the dielectric permittivity
δ_{HP}	the thickness of the space between the electrode and the HP
a_t	the lattice size
μ_i^0	the chemical potential under standard conditions
e_0	the elementary charge
$\mathrm{k_B}$	the Boltzmann constant
n_i	the number density of ion i
r_i	the radius of solvated ion
R_{s}	the diameter of solvent
K	spring constant of the cantilever
J	current density
g(r)	the radial distribution function
ρ	the number density
S_2	the Two-Body Excess Entropy
σ	the ionic conductivity
D	the self-diffusion coefficients

Supplementary Table 2. Ionic Conductivity for glyme-Li salt complexes at 30 °C

Glyme-Li salt mixture	$\sigma_{sim*.}$ (mS cm^{-1})	$\sigma_{ref.}$ (mS cm^{-1})
Li(G4)FSI	2.20	1.4[5]
$Li(G4)_3FSI$	9.89	~3.5[6]

σ: ionic conductivity. σ_{sim*} are calculated from the Einstein–Helfand formalism[7], whose trend is reasonable, while the remaining deviation in absolute value is likely due to the limited 10 ns MLMD trajectory length. The Einstein–Helfand relation tracks the net charge flux of the entire simulation box as a single collective particle, which suffers from severe statistical fluctuations and typically requires much longer trajectories to fully average out the noise and achieve high quantitative precision. Therefore, the σ_{sim*} values presented here are intended for qualitative reference and trend comparison.

Supplementary references

1 Zhang, L.-L., Li, C.-K. & Huang, J. A Beginners' Guide to Modelling of Electric Double Layer under Equilibrium, Nonequilibrium and AC Conditions. *J. Electrochem.* **28**, 2108471, doi:10.13208/j.electrochem.210847 (2022).

2 Kornyshev, A. A. Double-Layer in Ionic Liquids: Paradigm Change? *J. Phys. Chem. B* **111**, 5545-5557 (2007).

3 Zhang, Z. & Huang, J. Microstructure of Electrical Double Layers at Highly Charged States. *JACS Au* **5**, 3453-3467, doi:10.1021/jacsau.5c00508 (2025).

4 Senden, T. J. Force microscopy and surface interactions. *Current Opinion in Colloid & Interface Science* **6**, 95-101 (2001).

5 Ueno, K. *et al.* Glyme-Lithium Salt Equimolar Molten Mixtures: Concentrated Solutions or Solvate Ionic Liquids? *J. Phys. Chem. B* **116**, 11323-11331, doi:10.1021/jp307378j (2012).

6 Terada, S., Ikeda, K., Ueno, K., Dokko, K. & Watanabe, M. Liquid Structures and Transport Properties of Lithium Bis(fluorosulfonyl)amide/Glyme Solvate Ionic Liquids for Lithium Batteries. *Australian Journal of Chemistry* **72**, 70-80, doi:10.1071/ch18270 (2019).

7 Blazquez, S. *et al.* Computation of Electrical Conductivities of Aqueous Electrolyte Solutions: Two Surfaces, One Property. *J Chem Theory Comput* **19**, 5380-5393, doi:10.1021/acs.jctc.3c00562 (2023).
