# Supplementary material for: Nonmonotonic Screening and Solvation Dynamics of the Electrical Double Layer in Concentrated Lithium Salt Electrolytes
Source: Nat Commun. 2026 Jul 29;17:7587. doi: 10.1038/s41467-026-75999-2 (PMC13421668; doi:10.1038/s41467-026-75999-2)
Supplement: Supplementary file 3 — Transparent Peer Review file [file 41467_2026_75999_MOESM3_ESM.pdf]

nature portfolio

Peer Review File

Nonmonotonic Screening and Solvation Dynamics of the Electrical Double Layer in Concentrated Lithium Salt Electrolytes

Corresponding Author: Professor Jiawei Yan

This file contains all reviewer reports in order by version, followed by all author rebuttals in order by version.

Version 0:

Reviewer comments:

Reviewer #1

(Remarks to the Author)
This work combines electrochemical methods, in situ gap-enhanced Raman spectroscopy, and molecular dynamics simulations to model and demonstrate that, in lithium-ion battery non-aqueous conditions, the electrochemical double layer behaves differently than in the classical GPS model (assuming diluted ions in aqueous solutions, for example). The study involves Li+ cations, bis(fluorosulfonyl)imide anions, [FSI]-, and tetraglyme as solvent.

The results are noteworthy, but I got the impression that the title and instruction should have focused more on the fact that this pertains, in this exact scenario, to lithium-ion batteries.

The study seems original and clearly builds up on a recent trend in theoretical developments on the electrochemical double layer with concentrated electrolytes. The main claim for novelty is that the anions, influenced by concentration dependent structures, can set the behaviour of the lithium cations at the interface of the intercalation process. This was shown by considering the solvation entropy using machine-learning molecular dynamics and electrochemical atomic force microscopy.

In my opinion, the work offers new insights for lithium-ion technologies. Other papers have used electrochemical atomic force microscopy (https://www.nature.com/articles/s41467-021-24297-0) but not enhanced, also machine learning (https://arxiv.org/html/2501.07321v1) but not validated by experiments nor explicitly mentioning the role of each ion. The work compares well with current activity in the field, seems to advance previous leading discussion on concentration effects (https://www.nature.com/articles/s41467-022-32794-z), which was an opinion work suggesting broad studies such as the one here submitted.

As far as I can see, the work provides sufficient evidence for the claims it makes. My main objection is the relative inadequacy of the title and introduction, which should emphasize that this work pertains lithium-ion batteries (even though this is useful for the larger electrochemistry community). Yet this is mainly clarity in writing. My second objection is the somewhat simple treatment of electrochemical details in the methodology, which is below the overall quality of the work. Details on temperature and charge-discharge regime. I could not find obvious errors and the literature was quite appropriate.

In summary, I suggest publication after moderate corrections.

Specific comments:

Abstract

The abstract mentions Li+ ions out of nowhere. The title gives the impression that this article treats the general EDL concept, universal for all electrode reactions and solvent. Yet there is no indication that the work relates in particular to lithium-ion battery (LIB) technologies. The authors omit the fact that EDL applies to the whole field of electrochemistry.

Introduction

Again, practically all of the references in page 1 are related to LIB, yet these paragraphs do not mention that the EDL in question and the particularities summarized or claimed refer only in relation to LIB and non-aqueous solvents.

P2/L37, further, there is only such SEI decomposition in LIB.

P3/L59, at this point is the first mention of intercalation and that this work pertains to these types of electrode processes.

Results and discussion

Temperature for the electrochemical experiments is not reported, not in the Methods section nor in the SI figures or the articles main figures. “Room temperature” is mentioned several times but this is not useful in an article that focuses on such temperature-dependent physicochemical phenomena. Indeed, in line 387 a temperature 298.15 K is mentioned for a calculation, but all other data is measured or calculated seemingly at an unknown accurate temperature.

In the caption for Fig 2, I find that the use of the word “synergistic” is unnecessary.

P13/L325 Comment on the uncertainty of the experimental and modelled population at the PZC (Fig 3g).

P16/L325 Was this a galvanostatic cycling regime? Or did it include potentiostat steps to obtain the total accessible capacity?

P17/L429 I would suggest giving a quantitative assessment of the cycling instead of using the word “excellent”, which is too subjective.

P17/L435-38 I would suggest giving a quantitative assessment of the cycling instead of using the word “excellent”, which is too subjective.

In the caption for Fig 5, make sure you mean “GCS-regime” and not “non-GCS-regime”. It is not clear form where in the discussion the authors introduce “reversible” and “irreversible” in the caption, please clarify to which exact process are they referring to.

Conclusions

P18/L452 Please modify the text to ascertain that this is for LIB. “… on the interfacial electrical double layer in LIB.”

Methods

In Electrochemical Measurements, the exact temperature is missing. More importantly, there is no detailed information on the charge-discharge of the coin cells (galvanostatic, galvanostatic-potentiostatic, etc.).

Reviewer #2

(Remarks to the Author)
This work combines electrochemical and Raman spectroscopy experiments with molecular dynamics simulations to reveal deviations from the classical electrical double layer theory when highly concentrated Li+ electrolytes are used; this then allowed the authors to show the effect of increased solvation entropy on the solvation environment on highly concentrated electrolytes. This work has the potential to drive electrolyte design, and as such I believe it fits quite well within the scope of the journal. Please find below my comments about the validity and importance of this work, as well as suggestions to improve the manuscript.

Importance
The manuscript shows how increased solvation entropy affects the solvation on highly concentrated electrolytes. This was simulated with molecular dynamics and tested with electrochemical, gap-enhanced Raman spectroscopy and atomic force microscopy experiments.

The authors have perform a sound and deep analysis, including the mathematical derivation of the Gouy-Chapman capacitance. They explicitely show the differences between the double layer capacitance in low and high concentrated electrolytes, where in the latter, the classical Gouy-Chapman model does not apply.

The effect of highly concentrated electrolytes on double layer capacitance is rarely talked about in the literature, specially in lithium-ion batteries, and as such, I applaud the authors for tackling this problem. This work can impact the way future electrolytes are designed.

To test their hypothesis, the authors used a range of experimental techniques and simulations:
electrochemical (cyclic voltammetry and impedance), force-distance curves from atomic force microscopy, in-situ gap-enhanced Raman spectroscopy and even molecular dynamics simulations. The collection of techinques generated a huge ammount of data for the different electrolyte concentrations used plus the repetition of the experiments, which according to the manuscript were 20. In combination, the data obtained from different techniques, helped them confirm the solvation structure as a function of electrolyte concentration.

# Comments and suggested improvements.
* Lines 216 to 217: What do you mean by heatmaps here? To me this imply 2D mapping. Please add a reference to the figure where this is shown.
* Lines 218 to 220: it would be good to have a typical AFM approach curve in the SI indicating where the rupture force is being taken from.
* Lines 221 to 223: when talking about steps, does this mean cantilever step due to the interaction with the material (1D) or material step (2D)? My feeling is that this is obtained from approach curves, not 2D scanning.
* Line 257: due to the redshift, it is difficult to follow the explanation with Raman shift numbers. I suggest adding labels in Fig S16 (and others) to explicitely show what region is being referred to.
* Lines 263 to 264: it says "... while the SO2 peak becomes significantly more pronounced ...", this is not obvious to me. On the contrary, it seems as the peak is decreasing in magnitude when the potential is increased. Please clarify.
* Line 416: where are these results shown?
* Line 512 to 513: does this mean the 20 force curves where obtained in the same position? Also, it says that the 20 curves were obtained for each applied potential, however, in the electrochemical measurements section it says that CV and EIS were applied, which means that the potential was constantly changing. Please clarify.
* Pleas add a table of symbols and their definitions, both in the SI and main text since both have mathematical terms. The
mathematical derivation is hard to follow when there are so many undefined terms.
* Please increase the font size in the figures, some number in axis, labels, etc are too small.
* Line 256: it says 1145 cm-1, do you mean 1150? This is what is indicated in Fig S16.
* Line 261: do you mean 292 cm-1?
* Figure 2: this figure (and all of them, actually) is too crowded. Consider putting some of these into the SI and only leaving here the most relevant information.
The term "heatmap" in lines 216 to 217 had me confused. Is Figure 2a a real heatmap or a superposition of all 20 force-distance curves? What do the green-red colours mean? Please clarify.
* Figure 2a: the combination of blue with bright green and red is very bright, please consider changing these colors and improve the font, it looks too pixelated and too small. This is true for the rest of the figure and the other ones as well.
* SI Tables 1-2: I believe a plot comparing both would be better for visualization
* Line 384: this equation looks to ackward inline, consider having it on its own.
* Supplementary Figures 2 to 6: please improve them, the text is barely visible.

Reviewer #3

(Remarks to the Author)
In this work, the authors have conducted in situ gap-enhanced Raman spectroscopy to probe the electrical double layer (EDL) of the the lithium battery electrolytes composed of LiFSI and G4 with various concentrations, namely Li(G4)3FSI and Li(G4)FSI. Machine-learning molecular dynamics (MLMD) have also been performed to demonstrate the solvation structures and dynamics of lithium ions in the Li(G4)3FSI and Li(G4)FSI. Despite the efforts made in this work, I suggest rejection of this manuscript for publication in the prestigious journal of Nature communications due to the following concerns:

(1)The authors claimed they have revealed the anomalous screening that is beyond the GCS theory, which has been reported by many previous works (PNAS, 2023, 110, 9674-9679; PNAS, 2015, 7432-7437) in some electrolyte systems, not to mention that the concept of anomalous screening still remains under strong debate (Electrochemistry Communications, 2017, 82, 129-133; https://arxiv.org/abs/2408.15685). I do not see new finding in this work in terms of the so-called anomalous screening of the concentrated electrolytes.

(2)The authors also claimed they have revealed dynamic exchange phenomena in the EDL across varying salt concentrations. However, the related conclusion was made based on the solvation shell exchange of the bulk solutions with different salt concentrations by MLMD simulations, which has nothing to do with the EDL. There is no doubt there will be more anion that enters into the Li ion solvation shell with increasing salt concentration.

(3)The schematic for the EDL structure as presented in Figure 2a does not make sense. For example, in the case of E=2.1 V and E=2.5 V (below pzc), some adsorbed Li+ ions on top of the negatively charged electrode surface would be expected, however, I do not see any of them in the picture. Is there any physical explanation for this? How did the authors conduct the measurement of the EDL, and how did the authors know the exact EDL structures only from the experimental measurement? MD simulations of EDL will be necessary to give deep insights of the EDL structures like it has been done in previous works (Acc. Chemem. Res., 2017, 50, 2886–2894; Energy Environ. Sci., 2025, 18, 3036).

Reviewer #4

(Remarks to the Author)
In this manuscript, Yin et al. report a study on the electrochemical double-layer that forms when Li salt concentrated electrolytes are put in contact with graphite electrode. It combines electrochemistry measurements (impedance and cyclic voltammetry) with atomic force microscopy, surface-enhanced Raman spectroscopy and molecular dynamics simulations. Although the approach and the systems are interesting, I think that there are many inconsistencies that undermine the conclusions as well as the originality of the work. Consequently, I do not recommend publication in Nature communications and I recommend the authors to address the following points before resubmitting the manuscript to any journal.

1) The main result from which the authors conclude on an “anomalous screening” is based on the Parsons-Zobel plots shown on Figure 1a and 1b. However, contrarily to the authors claim (page 5, line 129, they state that it remains an open question), it is well established that the Gouy-Chapman-Stern theory does not work for concentrated electrolytes, from a vast literature of theory, simulation and experimental results. It is therefore not relevant to calculate the corresponding capacitance for the present systems. The results are compared to previous studies on diluted electrolytes (for example the green dots on Figure 1a were obtained for concentrations up to 5mM only), for which it was much more meaningful. In addition, the double-layer capacitance arises from a fit of the impedance spectroscopy using a six-parameter equivalent circuit model. The impedance spectra shown in the SI do not seem very different from one concentration to another, and it is possible that there are some error compensations arising from the fit. Normally, some of the parameters should remain constant from one system to another but the authors do not provide the results of the fit so it is difficult to check this.

2) The authors mention that “However, most studies treat solvation behavior and intercalation processes independently, overlooking their mutual influence”, suggesting that it is not the case in the present study. However, most of the analysis are made in the absence of intercalation so the approach is not very different from previous works.

3) The analysis of the AFM results is not very clear. The authors conclude on the composition of the first layer based on the obtained layer thickness, but they do not provide the geometric properties of the ions / diglyme in the system so it is not easy to follow. It would be good to provide a plot in which all the concentrations are shown, as in the current form all the plots are separated in the SI. It would also be good to explain how precise are the measurements and the analysis. For example, when I look at Figure 2.a, the middle panel looks to me very similar to the other ones so why are the two thicknesses inverted?

4) I do not understand from which simulations do the results on Figure 3g come. From the methods section it looks like the authors only performed MD simulations of bulk liquids, so where does the PZC come from here?

5) Following the previous questions, did the authors consider simulating the electrolytes at the interface with carbon? This could provide a very quantitative information for analysis the AFM (and maybe the Raman as well)

6) The claim of entropy being the “design lever for optimizing solvation dynamics and guiding next generation electrolyte development” is mostly based on an analysis of the radial distribution functions using a modified Kirkwood functional. However, when looking at ref. 56, the latter was only used for bcc iron, and there is no proof that it remains valid for ionic systems as we know the latter are remarkably difficult to represent using conventional liquid state theory (in particular at high concentrations, where they start to have ionic liquids-like properties).

7) In the methods section the authors mention calculating the conductivity and viscosity of the liquids, but the data is not provided. How does it compare with experiments?

8) The authors point towards ref. 62 for the force field, but the latter does not give the ML model (maybe as it is not published yet). Please provide the model for the current system to ensure reproducibility of the results.

Version 1:

Reviewer comments:

Reviewer #1

(Remarks to the Author)

I have read the rebuttal letter with change log, updated supplementary information, and the new version of the manuscript. The authors have made all necessary modifications to address my comments. I have no further observations.

Reviewer #2

(Remarks to the Author)
I am happy with the response and changes made by the authors. I am specially pleased with their response to Comment 5. I recommend publication.

Reviewer #3

(Remarks to the Author)
I have carefully reviewed the response letter and the revised manuscript. Regrettably, several critical concerns remain inadequately resolved, and I hold the opinion that this manuscript is not suitable for publication in Nature Communications. I suggest rejection of this work.

Since the authors wanted to leverage the ab initio accuracy of MLMD, why don't they use MLMD to conduct simulations of the EDL? In terms of the constant-potential classical MD simulations of the EDL, I have big concerns about the methods reported in the revised manuscript. For example, the authors did not provide the details for the OPLS-AA force field, and it looks like the author did not apply any charge scale to the Li+ anions and anion species, which tend to overestimate the ion pairs (Phys. Chem. Chem. Phys., 2011,13, 2613-2626).

How were SSIP, CIP, and AGG accounted for in the MD simulations of the EDL? The authors reported MD-simulated SSIP, CIP, and AGG in comparison with experimental data, what about the data under other voltage conditions?

In the reply, the authors claimed "The new interfacial simulation results clearly demonstrate that the trend observed in the bulk is preserved and even amplified within the EDL. Under polarization, the exchange frequency between Li+ and anions in the high-concentration system is significantly higher than that in the dilute system. The CPM results confirm that the "looser" solvation sheath identified by MLMD facilitates easier desolvation and reorganization at the interface, directly supporting the mechanism of enhanced reversibility." I do not see data that supports those statements.

The authors used the Nernst-Einstein equation to calculate the ionic conductivity. Despite they claim the calculated ionic conductivity is close to the experimental value, it is widely known that the Nernst-Einstein equation does not consider ion correlation and thus does not apply to the highly-concentrated electrolytes at all (ACS Cent. Sci. 2019, 5, 7, 1250–1260).

Reviewer #4

(Remarks to the Author)
I think that the additional experiments/simulations and corrections made by the authors have clearly improved the manuscript, that can now be published in Nature Communications. I just have a few comments that remain to be addressed:

Regarding the response to my comment #1, EIS fit: The authors did not answer my point. They show that their fit is good by comparing with another equivalent circuit, which I never asked. My question was how sensitive was the fit with respect to the parameters variations, keeping the initial equivalent circuit. This can be done by looking at the covariance matrix, or even better by performing a global sensitivity analysis. In particular I would be interested in seeing how sensitive is the fit to the various contributions to the capacitance.

Regarding the response to my comment #4: The authors mention: "To address this limitation and provide a rigorous calculation of the PZC, we have incorporated Classical Molecular Dynamics simulations using the Constant Potential Method (CPM) in the revised manuscript. The results presented in Figure 3g (in the revised version) are now explicitly derived from
these new CPM simulations, which allow us to simulate the open-circuit conditions and determine the electrode potential at zero surface charge density". However, the constant potential method corresponds to a fixed potential and not to open-circuit conditions. Can the authors update the method description accordingly?

Additional comments:
-In the methods section, reference 70 (doi:10.1103/PhysRevLett.110.196801) is quoted for the CPM calculations, but the corresponding article is not using/implementing this method. Please correct.

Version 2:

Reviewer comments:

Reviewer #3

(Remarks to the Author)

I appreciate the authors' efforts to reply to the questions, but I still have big concerns about the simulation part. The reliability of the simulation data is highly doubted. I won't suggest publication of this work before these concerns are thoroughly addressed.

The authors tested a charge-scaling treatment by applying a uniform scaling factor of 0.8 to the original ionic charges, but found larger Li–O coordination number between Li+ and the oxygen atoms in FSI– compared to the case without charge scaling. This is awkward. A charge scaling of 0.8 must weaken the intertactions between Li+ and FSI–.

Why is the ionic conductivity calculated from the Einstein–Helfand formalism (2.2 mS/cm) even higher than that from Nernst-Eistein equation? This does not make sense. The ionic conductivity must drop, sometimes significantly, when ion correlation is considered, particularly for high concentration electrolytes (ChemElectroChem, 2020, 7 , 367 —385).

Reviewer #4

(Remarks to the Author)
The authors have well addressed my comments. I recommend publication in Nature Communications.

Version 3:

Reviewer comments:

Reviewer #3

(Remarks to the Author)
I appreciate the authors' efforts to address these concerns. These simulation details should be added to the Supplementary Information, which should have been done at the very beginning, so that the simulation results can be reproduced. After that, I would suggest publication of this work. The next round review is necessary.

Open Access This Peer Review File is licensed under a Creative Commons Attribution 4.0 International License, which permits use, sharing, adaptation, distribution and reproduction in any medium or format, as long as you give appropriate credit to the original author(s) and the source, provide a link to the Creative Commons license, and indicate if changes were made.
In cases where reviewers are anonymous, credit should be given to 'Anonymous Referee' and the source.
The images or other third party material in this Peer Review File are included in the article's Creative Commons license, unless indicated otherwise in a credit line to the material. If material is not included in the article's Creative Commons license and your intended use is not permitted by statutory regulation or exceeds the permitted use, you will need to obtain permission directly from the copyright holder.
To view a copy of this license, visit https://creativecommons.org/licenses/by/4.0/

Response to Reviewers

We would like to sincerely thank the reviewers for their positive evaluations and for providing insightful and valuable comments on our manuscript. We are grateful for the opportunity to clarify the points raised and to further improve our work. In response to the reviewers' suggestions, we have conducted additional experiments, refined our analyses, and carefully revised the manuscript. We believe that these revisions have significantly strengthened the quality and clarity of the paper. A complete set of point-by-point responses to the reviewers' comments is provided below. For clarity, all revisions in the manuscript are highlighted in yellow.

Reviewers' comments and our Response

Reviewer #1 (Remarks to the Author):

This work combines electrochemical methods, in situ gap-enhanced Raman spectroscopy, and molecular dynamics simulations to model and demonstrate that, in lithium-ion battery non-aqueous conditions, the electrochemical double layer behaves differently than in the classical GPS model (assuming diluted ions in aqueous solutions, for example). The study involves Li^+ cations, bis(fluorosulfonyl)imide anions, $[FSI]^-$, and tetraglyme as solvent.

The results are noteworthy, but I got the impression that the title and instruction should have focused more on the fact that this pertains, in this exact scenario, to lithium-ion batteries.

The study seems original and clearly builds up on a recent trend in theoretical developments on the electrochemical double layer with concentrated electrolytes. The main claim for novelty is that the anions, influenced by concentration dependent structures, can set the behaviour of the lithium cations at the interface of the intercalation process. This was shown by considering the solvation entropy using machine-learning molecular dynamics and electrochemical atomic force microscopy.

In my opinion, the work offers new insights for lithium-ion technologies. Other papers have used electrochemical atomic force microscopy (https://www.nature.com/articles/s41467-021-24297-0) but not enhanced, also machine learning (https://arxiv.org/html/2501.07321v1) but not validated by experiments nor explicitly mentioning the role of each ion. The work compares well with current activity in the field, seems to advance previous leading discussion on concentration effects (https://www.nature.com/articles/s41467-022-32794-z), which was an opinion work suggesting broad studies such as the one here submitted.

As far as I can see, the work provides sufficient evidence for the claims it makes. My main objection is the relative inadequacy of the title and introduction, which should emphasize that this work pertains lithium-ion batteries (even though this is useful for the larger electrochemistry community). Yet this is mainly clarity in writing. My second objection is the somewhat simple treatment of electrochemical details in the methodology, which is below the overall quality of the work. Details on temperature and charge-discharge regime. I could not find obvious errors and the literature was quite appropriate.

In summary, I suggest publication after moderate corrections.

Response: We greatly appreciate the reviewer's positive assessment of the novelty of our work and the recommendation for publication after moderate revisions. We sincerely thank the reviewer for the constructive and supportive comments.

We fully agree that the title and introduction should more clearly communicate that this study specifically focuses on lithium-ion batteries, despite its broader significance to the electrochemistry community. In the revised manuscript, we have therefore updated the title and rewritten portions of the introduction to explicitly emphasize the lithium-ion battery context and to improve overall clarity.

We also appreciate the reviewer's insightful suggestion regarding the electrochemical methodology. In response, we have substantially expanded this section to include more detailed descriptions of the experimental conditions, such as temperature control, the charge-discharge protocol, and other relevant electrochemical parameters. These additions strengthen the rigor and completeness of the Methods section and better align it with the overall quality of the work.

Comment 1: Abstract—The abstract mentions Li^+ ions out of nowhere. The title gives the impression that this article treats the general EDL concept, universal for all electrode reactions and solvent. Yet there is no indication that the work relates in particular to lithium-ion battery (LIB) technologies. The authors omit the fact that EDL applies to the whole field of electrochemistry.

Response: Thank you very much for this comment. In the revised manuscript, the title is changed to "Electrical Double Layer in Concentrated Lithium Salt Electrolytes: Nonmonotonic Screening and Solvation Dynamics". We also have rewritten the beginning of the abstract to clearly state that the study focuses on lithium-ion battery electrolytes, so that the subsequent discussion of Li^+ becomes natural and properly framed within its electrochemical context. The

revised abstract now begins as follows:

"Understanding the electrical double layer (EDL) in lithium-ion battery electrolytes is fundamental to improving interfacial kinetics and stability which determine battery performance and lifetime. However, the microstructure and dynamics of the EDL in highly concentrated lithium salt solutions remain elusive. Herein, combining electrochemical analyses, in situ gap-enhanced Raman spectroscopy and molecular dynamics simulations, we reveal nonmonotonic variation of electrostatic screening with concentration and different exchange kinetics of anions and solvent molecules in the solvation sheath of lithium ions. These results are interpreted with a mechanism that increased solvation entropy favors the formation of an anion-rich solvation environment in highly concentrated electrolytes. The structural reorganization enhances the exchange frequency between Li^+ and anions relative to Li^+ and solvent. This accelerated coordination exchange allows Li^+ to reorganize its solvation sheath more flexible when approaching or leaving the electrode surface, thereby improving the kinetics and reversibility of Li^+ intercalation/deintercalation. Overall, this work provides novel thermodynamic and kinetic insights for the electrolyte design of advanced lithium-ion battery systems." (Page 1, Line 16)

Comment 2: Introduction—Again, practically all of the references in page 1 are related to LIB, yet these paragraphs do not mention that the EDL in question and the particularities summarized or claimed refer only in relation to LIB and non-aqueous solvents.

P2/L37, further, there is only such SEI decomposition in LIB.

P3/L59, at this point is the first mention of intercalation and that this work pertains to these types of electrode processes.

Response: Thank you very much for this comment. We agree that, although the introduction cited literature primarily from the lithium-ion battery field, it did not explicitly state that the EDL phenomena discussed were specific to LIB systems and non-aqueous electrolytes. This may have given the impression that the described interfacial behavior was intended to apply broadly across all electrochemical systems, which was not our intention.

In the revised manuscript, we have clarified this point by explicitly stating in the introduction that the focus of this work is the electrical double layer formed in lithium-ion battery electrolytes, particularly in non-aqueous and highly concentrated electrolyte systems. We have added text to emphasize that the interfacial features, ion correlations, and solvation behaviors summarized in the

introduction pertain specifically to LIB-relevant environments rather than to general EDL theory applicable to aqueous or dilute systems.

"At the electrode/electrolyte interface, the electrical double layer (EDL) regulates the local electrostatic and chemical environment in which interfacial reactions occur, including the electrolyte reduction reactions that ultimately govern the formation and composition of the solid electrolyte interphase (SEI) in lithium-ion batteries (LIBs)." (Page 2, Line 36)

"The relationship between the EDL, ion solvation structures, and electrochemical performance in concentrated LIBs electrolytes remains unclear." (Page 2, Line 41)

"Viewing the EDL through this combined framework of interfacial structure and entropic effects yields a more continuous and physically grounded description of screening, thereby overcoming key limitations of the classical GCS model in practical lithium-ion battery electrolytes." (Page 2, Line 58)

"Within the EDL of lithium-ion batteries, the interfacial solvation environment governs Li^+ desolvation, a key step prior to its intercalation into the host material." (Page 2, Line 62)

Comment 3: Results and discussion—Temperature for the electrochemical experiments is not reported, not in the Methods section nor in the SI figures or the articles main figures. "Room temperature" is mentioned several times but this is not useful in an article that focuses on such temperature-dependent physicochemical phenomena. Indeed, in line 387 a temperature 298.15 K is mentioned for a calculation, but all other data is measured or calculated seemingly at an unknown accurate temperature.

Response: We thank the reviewer for highlighting the importance of clearly specifying the experimental temperature. In the revised manuscript, we have now provided the exact temperature conditions for all electrochemical measurements. Specifically, all CV, EIS, EC-AFM, and Raman experiments were performed at 298 ± 1 K, maintained by the thermostated environment of the argon-filled glovebox. This temperature information has been added to the Methods section. These clarifications ensure full consistency between the temperature used in experimental measurements and the temperature applied in theoretical calculations.

Comment 4: In the caption for Fig 2, I find that the use of the word "synergistic" is unnecessary.

Response: Thank you very much for this comment. In the revised manuscript, we have removed the term "synergistic" from the captions of both Fig. 2 and Fig. 3.

Comment 5: P13/L325 Comment on the uncertainty of the experimental and modelled population at the PZC (Fig 3g).

Response: Thank you very much for this comment. To address this limitation and enable a rigorous treatment of interfacial electrostatics, we have now incorporated additional classical molecular dynamics simulations using the constant potential method (CPM) in the revised manuscript. We acknowledge that minor quantitative differences are observed in the relative populations of CIP, SSIP, and AGG at the PZC between the simulations and the Raman results (Fig. 3g). These discrepancies arise from intrinsic limitations of the theoretical methods and sampling constraints. In particular, although the CPM provides a physically realistic description of electrode polarization, it does not fully resolve the subtle free-energy differences between closely related coordination environments. In addition, the simulations were performed on systems of approximately 15,000 atoms over 10 ns; given the highly dynamic nature of ionic association and exchange processes, such finite spatiotemporal sampling inevitably introduces statistical uncertainty in the extracted populations. Importantly, despite these quantitative differences, the overall trends and the underlying physical picture of the solvation structure at the PZC remain fully consistent between simulations and experiments. Furthermore, the ionic conductivity derived from the simulated diffusion coefficients aligns well with the experimental measurements, confirming that the simulation successfully captures the essential macroscopic transport behavior and is reliable for qualitative analysis.

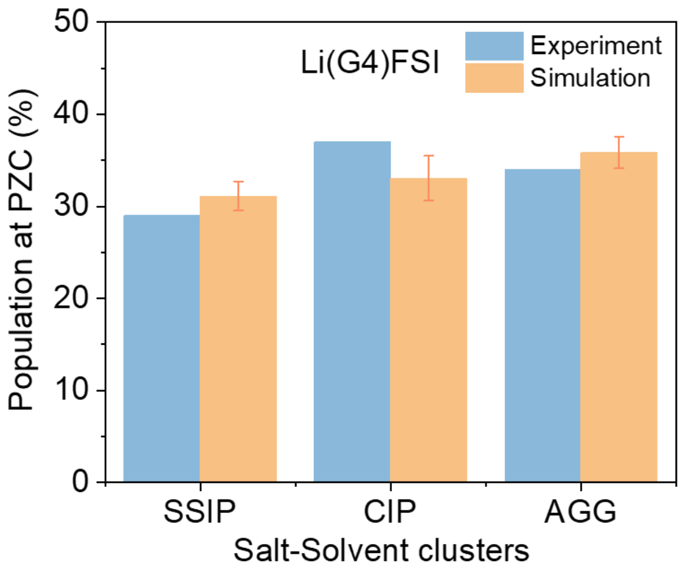

Figure R1–1, Comparison of interfacial structural characteristics at E=0.0 V obtained from experiment (blue) and simulations (orange). Simulation data represent averages over 200 independent samples, with error bars indicating statistical uncertainty. Minor discrepancies can be attributed to limitations of the OPLS/AA force field and residual uncertainties in both experiment and simulation. (Page 16, Line 402)

Comment 6: P16/L325 Was this a galvanostatic cycling regime? Or did it include potentiostat steps to obtain the total accessible capacity?

Response: Thank you very much for this comment. The cycling protocol used in this work was purely galvanostatic, without any additional potentiostatic holding steps at the upper or lower cutoff voltages. Therefore, the reported capacities correspond to the reversible capacity accessed under constant current conditions within the specified voltage window. We have now clarified this explicitly in the revised Methods section.

Comment 7: P17/L429 I would suggest giving a quantitative assessment of the cycling instead of using the word "excellent", which is too subjective.

Response: Thank you very much for this comment. To provide a quantitative assessment, we have replaced it with explicit cycling metrics. Specifically, the electrode delivers a reversible capacity of 320 mAh g^{-1} under the tested conditions.

"After 100 cycles, the delithiation capacity remained at 320 mAh g^{-1}, indicating stable Li^+ intercalation and deintercalation in the graphite electrode. This suggests the formation of a favorable interface between the graphite electrode

and the Li(G4)FSI electrolyte." (Page 19, Line 490)

Comment 8: P17/L435-38 I would suggest giving a quantitative assessment of the cycling instead of using the word "excellent", which is too subjective.

Response: Thank you very much for this comment. We have carefully re-checked the manuscript at the indicated location (P17, L435–438), and we fully appreciate the reviewer's suggestion regarding the use of quantitative rather than subjective descriptors. Accordingly, in the revised manuscript, we have further refined this paragraph by removing any potentially subjective wording and replacing it with explicit quantitative performance metrics.

Comment 9: In the caption for Fig 5, make sure you mean "GCS-regime" and not "non-GCS-regime". It is not clear form where in the discussion the authors introduce "reversible" and "irreversible" in the caption, please clarify to which exact process are they referring to.

Response: Thank you very much for this comment. We would like to clarify that in Fig. 5, the left schematic indeed corresponds to the highly concentrated electrolyte, where the electrical double layer behavior deviates from the classical Gouy-Chapman-Stern (GCS) model (non-GCS regime), whereas the right schematic corresponds to the dilute electrolyte that follows the classical GCS behavior. There is no mislabeling in the physical interpretation, but the original caption did not sufficiently emphasize this correspondence. We have therefore revised the figure caption to explicitly distinguish the non-GCS regime at high concentration from the GCS regime at low concentration.

Regarding the terms "reversible" and "irreversible," we clarify that these explicitly refer to the Li^+ intercalation/deintercalation behavior. In the highly concentrated electrolyte (left panel), Li^+ undergoes efficient desolvation prior to intercalation, enabling a reversible intercalation/deintercalation process. In contrast, in the dilute electrolyte following the GCS regime (right panel), Li^+ tends to enter the graphite together with solvent molecules due to insufficient desolvation, which induces co-intercalation, graphite exfoliation, and thus an irreversible process. Similar solvent co-intercalation and desolvation effects in graphite have been previously reported (J. Phys. Chem. C 2014, 118, 20246–20256). This clarification has now been added to the caption of Fig. 5.

"Fig. 5. Schematic illustration of the Li^+ transport mechanism and charge transfer process in electrolytes with various concentrations. Left: In the highly concentrated electrolyte (non-GCS regime), strong ion-ion correlations and

anion-rich solvation structures promote efficient Li^+ desolvation at the electrode interface, enabling a reversible Li^+ intercalation/deintercalation process in graphite. Right: In the dilute electrolyte following the classical Gouy−Chapman−Stern (GCS) regime, insufficient Li^+ desolvation leads to solvent co-intercalation into graphite, causing graphite exfoliation and irreversible interfacial processes." (Page 20, Line 510)

Comment 10: Conclusions—P18/L452 Please modify the text to ascertain that this is for LIB. "… on the interfacial electrical double layer in LIB."

Response: Thank you very much for this comment. In the revised manuscript, we have modified the corresponding sentence in the Conclusions section to explicitly specify that our findings pertain to the interfacial electrical double layer in lithium-ion batteries (LIBs). This revision ensures a clearer and more precise scope of applicability of our conclusions.

"In summary, a combined experimental and computational approach was taken to understand the influence of electrolyte concentration on the interfacial electrical double layer in non-aqueous lithium-ion battery systems." (Page 21, Line 517)

Comment 11: Methods—In Electrochemical Measurements, the exact temperature is missing. More importantly, there is no detailed information on the charge-discharge of the coin cells (galvanostatic, galvanostatic-potentiostatic, etc.).

Response: Thank you very much for this comment. In the revised manuscript, we have now explicitly specified the exact temperature. In addition, we have clarified the charge-discharge protocol of the coin cells in the Methods section.

"**Measurements of electrochemical performances**

All electrochemical tests were conducted using CR2016-type coin cells assembled in an Ar-filled glovebox. Each cell employed a single layer of Celgard 2325 as the separator and contained 100 μL of electrolyte. Galvanostatic charge-discharge measurements were carried out using a LANHE CT2001A battery testing system, with charging performed at 1 C and discharging at 0.1 C. For graphite||Li half-cells, the voltage window was set to 0.005~2.0 V (vs. Li^+/Li). All electrochemical measurements were carried out at 298 ± 1 K." (Page 24, Line 601)

Reviewer #2 (Remarks to the Author):

This work combines electrochemical and Raman spectroscopy experiments with molecular dynamics simulations to reveal deviations from the classical electrical double layer theory when highly concentrated Li+ electrolytes are used; this then allowed the authors to show the effect of increased solvation entropy on the solvation environment on highly concentrated electrolytes. This work has the potential to drive electrolyte design, and as such I believe it fits quite well within the scope of the journal. Please find below my comments about the validity and importance of this work, as well as suggestions to improve the manuscript.

Importance

The manuscript shows how increased solvation entropy affects the solvation on highly concentrated electrolytes. This was simulated with molecular dynamics and tested with electrochemical, gap-enhanced Raman spectroscopy and atomic force microscopy experiments.

The authors have performed a sound and deep analysis, including the mathematical derivation of the Gouy-Chapman capacitance. They explicitely show the differences between the double layer capacitance in low and high concentrated electrolytes, where in the latter, the classical Gouy-Chapman model does not apply.

The effect of highly concentrated electrolytes on double layer capacitance is rarely talked about in the literature, specially in lithium-ion batteries, and as such, I applaud the authors for tackling this problem. This work can impact the way future electrolytes are designed.

To test their hypothesis, the authors used a range of experimental techniques and simulations: electrochemical (cyclic voltammetry and impedance), force-distance curves from atomic force microscopy, in-situ gap-enhanced Raman spectroscopy and even molecular dynamics simulations. The collection of techinques generated a huge amount of data for the different electrolyte concentrations used plus the repetition of the experiments, which according to the manuscript were 20. In combination, the data obtained from different techniques, helped them confirm the solvation structure as a function of electrolyte concentration.

Response: We sincerely thank the reviewer for the positive and encouraging evaluation of our work and for recognizing its scientific significance and potential impact on electrolyte design. We appreciate the acknowledgement of the comprehensive experimental and computational approach, as well as the theoretical analysis of the double-layer capacitance in low- and high-concentration regimes.

Comments and suggested improvements.

Comment 1: Lines 216 to 217: What do you mean by heatmaps here? To me this imply 2D mapping. Please add a reference to the figure where this is shown.

Response: Thank you very much for this suggestion. In this work, the term "heatmap" refers to a two-dimensional histogram constructed from 20 force-separation curves, where force and separation are treated as the two axes and the color scale represents the statistical density of the data points. In the revised manuscript, we have replaced the heatmaps with profiles and explicitly indicated the corresponding figures (Fig. 2a and Fig. 3a).

"Representative force-separation profiles at each potential are presented in Fig. 2a and Fig. 3a, with each profile comprising 20 individual curves." (Page 9, Line 219)

Comment 2: Lines 218 to 220: it would be good to have a typical AFM approach curve in the SI indicating where the rupture force is being taken from.

Response: Thank you very much for this suggestion. As suggested, we have now added a representative AFM approach curve in the revised Supplementary Information to explicitly indicate how the separation is defined and extracted, as shown in Figure R2–1.

"**Force curve data analysis**

In AFM measurements, the interaction force is determined from the cantilever deflection recorded by the position-sensitive photodetector (PSD). The deflection signal (in volts) is converted into the cantilever deformation using the calibrated deflection sensitivity, according to

$$deformation\ (nm) = deflection\ (V) \times deflection\ sensitivity\ (nm \cdot V^{-1})$$

The interaction force is then obtained from Hooke's law $(f = k\Delta z)$, where k is the spring constant of the cantilever.

$$Force\ (nN) = k\ (nN \cdot nm^{-1}) \times deformation\ (nm)$$

The rupture force is defined as the maximum force observed immediately before the sudden drop in the force-separation curve." (SI, Page 5)

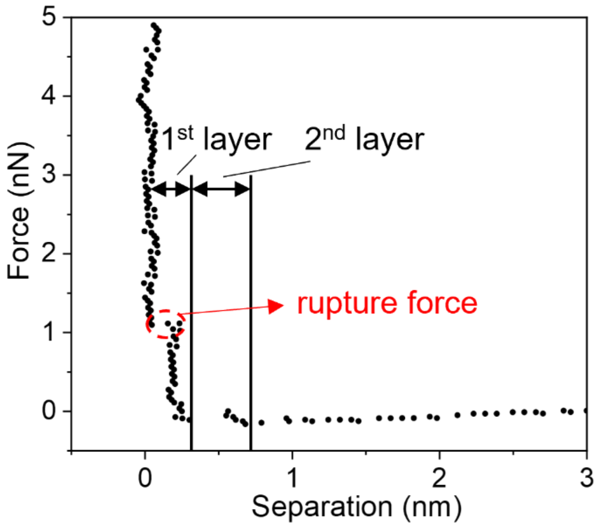

Figure R2–1. Example of a representative force-separation curve.

Comment 3: Lines 221 to 223: when talking about steps, does this mean cantilever step due to the interaction with the material (1D) or material step (2D)? My feeling is that this is obtained from approach curves, not 2D scanning.

Response: Thank you very much for this suggestion. Here, the "steps" refer strictly to the step-like features observed in the one-dimensional force-separation (approach) curves as your feeling, which originate from the sequential rupture or restructuring of interfacial solvation layers during tip approach. They do not correspond to two-dimensional material steps obtained from AFM topographical scanning, and we have clarified this point in the revised manuscript.

"The width of each step observed in the force-separation (approach) curves reflect the characteristic dimension of the interfacial species (anions, cations, or solvent molecules) involved in the layer rupture or rearrangement process, and thus serves as an indicator of the composition of the interfacial layer[42,43]." (Page 9, Line 225)

Comment 4: Line 257: due to the redshift, it is difficult to follow the explanation with Raman shift numbers. I suggest adding labels in Fig S16 (and others) to explicitly show what region is being referred to.

Response: Thank you very much for this suggestion. In the revised Supplementary Information, we have added a magnified view of the spectral region corresponding to the C-C stretching and CH_2 wagging mode. At E = 2.9 V, the peak is located at 1145 cm^{-1}, and it gradually redshifts to 1135 cm^{-1} as the potential is shifted negatively to E=2.1 V, as shown in Figure R2-2.

“Under stronger negative polarization (E shifting from 2.9 to 2.1 V_{Li}), the CH_2 peak profile gradually becomes similar to that of the pure solvent, and the peak assigned to the coupled C-C stretching and CH_2 wagging modes[50,51] redshift from 1145 cm^{-1} at E = 2.9 V_{Li} to 1135 cm^{-1} at E=2.1 V_{Li} (Supplementary Fig. 17).” (Page 10, Line 259)

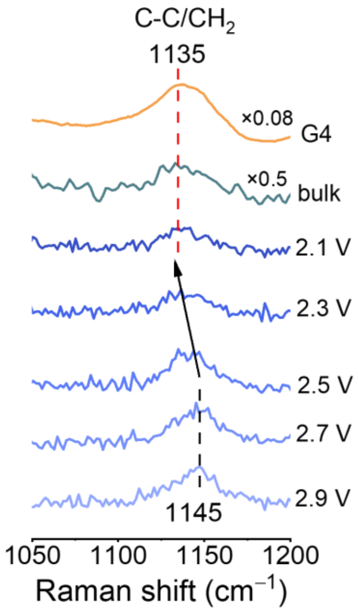

Figure R2-2, The spectra of the coupled C-C stretching and CH_2 wagging modes as the potential shifts toward more negative values. (SI, Page 22)

Comment 5: Lines 263 to 264: it says "... while the SO2 peak becomes significantly more pronounced ...", this is not obvious to me. On the contrary, it seems as the peak is decreasing in magnitude when the potential is increased. Please clarify.

Response: Thank you very much for this suggestion. We agree that the SO_2 peak does not monotonically increase in intensity over the entire positive polarization range, and our original wording was therefore not sufficiently precise. After re-examining the Raman spectra, we performed a detailed analysis of the S-F (~260 cm^{-1}) and SO_2 (~292 cm^{-1}) vibrations under positive polarization. When the potential is shifted from 3.0 to 3.3 V_{Li}, both the S-F and SO_2 peak intensities increase, indicating enhanced accumulation of FSI^- anions at the electrode surface. However, upon further increasing the potential from 3.3 to 3.7 V, the S-F peak continues to increase, whereas the SO2 peak intensity decreases. Meanwhile, the SO_2 vibration exhibits a clear blueshift from 292 cm^{-1} at 3.0 V to 298 cm^{-1} at 3.7 V. This behavior indicates a potential-induced reorientation of the FSI^- anion, in which the S-F moiety approaches the electrode surface while the SO_2 group tilts toward the solution side. As a result, the decrease in the SO_2 peak intensity at higher potentials arises from this configurational rearrangement, which enables a higher surface adsorption density of anions and simultaneously alleviates interfacial steric hindrance, as shown in Figure R2–3. We have revised the corresponding description in the

manuscript accordingly.

"Additionally, spectral peaks observed at 260 cm^{-1} and 292 cm^{-1} are attributed to the rocking vibrations of S-F and SO_2, respectively. A closer inspection of these bands under positive polarization reveals a two-stage potential dependence (Supplementary Fig. 18). As the potential is increased from 3.0 to 3.3 V_{Li}, the intensities of both the S-F and SO_2 peaks rise, consistent with enhanced accumulation of FSI^- at the electrode interface. Upon further increasing the potential from 3.3 to 3.7 V_{Li}, the S-F peak continues to grow in intensity while the SO_2 peak decreases; concurrently the SO_2 blueshifts from 292 cm^{-1} (E=3.0 V_{Li}) to 298 cm^{-1} (E=3.7 V_{Li}). These observations indicate a potential-driven reorientation of adsorbed FSI^- in which the S-F moiety approaches the electrode surface while the SO_2 group tilts toward the solution. Such reconfiguration promotes a higher surface adsorption density of anions and alleviates interfacial steric hindrance, confirming that the first interfacial layer is dominated by specifically adsorbed FSI^-." (Page 10, Line 265)

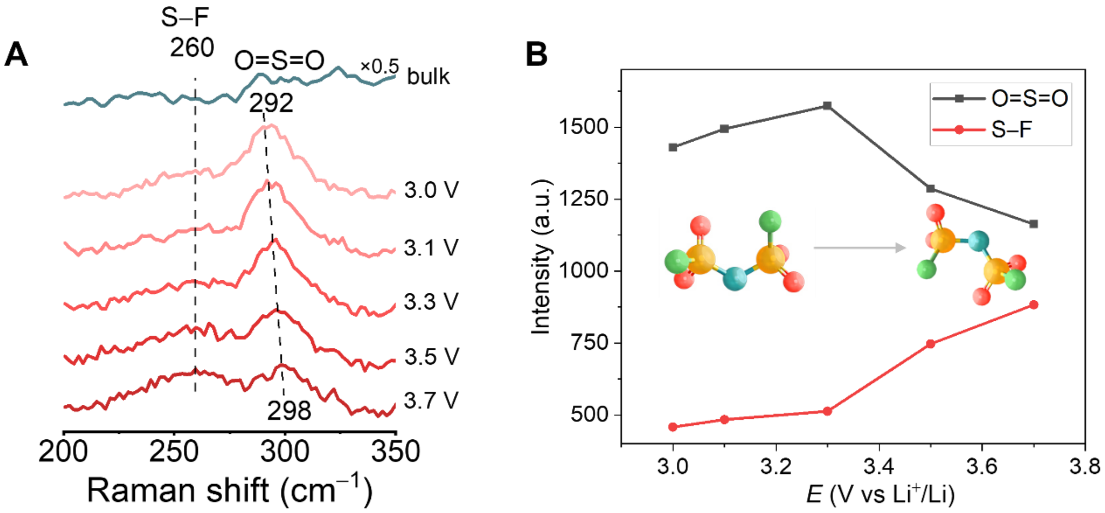

Figure R2-3, (**A**) Raman spectra of the S-F and SO_2 rocking modes under positive polarization. (**B**) Potential-dependent peak intensities of S-F and SO_2 modes. F, S, O, and N atoms are shown in green, yellow, red, and cyan, respectively. (SI, Page 23)

Comment 6: Line 416: where are these results shown?

Response: We thank the reviewer for pointing out this lack of clarity. We agree that the original wording in Line 416 was insufficiently specific regarding where the supporting results are presented.

In the revised manuscript, we have clarified this point as follows. The dynamic exchange frequency within the Li^+ solvation sheath, derived from MLMD simulations, is quantitatively shown in Figs. 4a–d, where the concentration-dependent evolution of solvation-sheath exchange events is analyzed. The corresponding experimental evidence for solvation structure evolution is provided by the *in situ* gap-enhanced Raman spectroscopy results in Figs. 2b–d and 3b–e, which reveal systematic changes in coordination environments and

anion participation.

The entropy-mediated mechanism discussed in Lines 416–419 is therefore not inferred from a single dataset, but emerges from the consistent trends observed across these complementary experimental and computational results. We have revised the text to explicitly reference the relevant figures and panels to avoid any ambiguity.

"Based on *in situ* spectroscopic experiments (Figs. 2b–d and 3b–e) and MLMD calculations (Figs. 4a–d), we elucidate an entropy-mediated mechanism governing solvation structure evolution and battery performance, by quantifying the dynamic exchange frequency within the Li^+ solvation sheath (Fig. 5)". (Page 19, Line 478)

Comment 7: Line 512 to 513: does this mean the 20 force curves where obtained in the same position? Also, it says that the 20 curves were obtained for each applied potential, however, in the electrochemical measurements section it says that CV and EIS were applied, which means that the potential was constantly changing. Please clarify.

Response: Thank you very much for this suggestion. The 20 force curves were not acquired at the same position. Instead, they were collected at multiple positions distributed over the entire scanned area (5×5 μm^2) to ensure that the results represent the average interfacial behavior of the electrode surface rather than a specific local site. In addition, the EC-AFM measurements were not performed simultaneously with CV or EIS. The force curves were acquired under constant potential control using an external electrochemical workstation connected to the EC-AFM cell. We have revised the corresponding description in the Methods section (Electrochemical AFM measurements) in the manuscript to clearly reflect these experimental procedures.

"**Electrochemical AFM measurement**

All force-distance measurements were performed using a Bruker Icon atomic force microscope operated inside an argon-filled glovebox (MIKROUNA, H_2O < 0.1 ppm, O_2 < 0.1 ppm) to eliminate moisture and oxygen interference. The electrochemical AFM (EC-AFM) configuration consisted of a three-electrode cell integrated with the AFM system. The working electrode, reference electrode, and counter electrode were immersed in the electrolyte and connected to a potentiostat (CHI660E, Chenhua Instruments) for constant-potential control. A CSG30 cantilever (TipsNano) with a spring constant of 0.6 N m^{-1} was used to acquire force curves. Before each experiment, the cantilever sensitivity and spring constant were calibrated against a standard reference surface (e.g., sapphire) under inert atmosphere to ensure accurate force quantification. A freshly cleaved 20 mm × 20 mm highly oriented pyrolytic graphite (HOPG, ZYB grade) served as the working electrode, while two lithium wires were used as the counter and reference electrodes, respectively. The electrochemical cell contained ~600 μL of electrolyte, and at least 20 force-distance curves were collected at each applied potential. The processing of the force curve data is

described in the Supplementary information. All measurements were conducted at 298 ± 1 K." (Page 23, Line 574)

Comment 8: Please add a table of symbols and their definitions, both in the SI and main text since both have mathematical terms. The mathematical derivation is hard to follow when there are so many undefined terms.

Response: Thank you very much for this suggestion. To improve the clarity and readability of the mathematical derivations, we have provided a table summarizing all symbols and their corresponding definitions in the Supplementary Information for convenient unified reference.

"**Supplementary Table 1. Basic model parameters**" (SI, Page 34)

Symbol	Definition
C_{H}	the inner layer or Helmholtz capacitance
C_{GC}	the diffuse layer capacitance
C_{ad}	the adsorption capacitance
C_{dl}	the double-layer capacitance
C_{tot}	the total capacitance
E	potential
$\epsilon_{\mathrm{s}}^{\mathrm{b}}$	the bulk dielectric permittivity of the electrolyte solution
ϕ	the electric potential
z_i	the charge number of ion i
F	the Faraday's constant
c_i	the concentration of ion i
c_i^{b}	the concentration of ion i in the solution bulk
R	the gas constant
T	the temperature
c^{b}	the concentration of total anions (cations) in the solution bulk
ϵ_{HP}	the dielectric permittivity
δ_{HP}	the thickness of the space between the electrode and the HP
a_{t}	the lattice size
μ_i^0	the chemical potential under standard conditions
e_0	the elementary charge
k_{B}	the Boltzmann constant
n_i	the number density of ion i
r_i	the radius of solvated ion
R_{s}	the diameter of solvent
f	force
k	spring constant of the cantilever
R	resistance
j	current density
$g(r)$	the radial distribution function
ρ	the number density
S_2	the Two-Body Excess Entropy

σ	the ionic conductivity
D	the self-diffusion coefficients

Comment 9: Please increase the font size in the figures, some number in axis, labels, etc are too small.

Response: Thank you very much for this suggestion. In the revised manuscript, we have increased the font size of all figures, including the axis numbers, labels, etc, to improve readability.

Comment 10: Line 256: it says 1145 cm^{-1}, do you mean 1150? This is what is indicated in Fig S16.

Response: Thank you very much for this suggestion. This issue has been addressed in our response to Comment 4, where we clarified the correct peak position and corrected the corresponding value in the revised manuscript to ensure full consistency between the main text and the Supplementary Information.

"Under stronger negative polarization (E shifting from 2.9 to 2.1 V_{Li}), the CH_2 peak profile gradually becomes similar to that of the pure solvent, and the peak assigned to the coupled C-C stretching and CH_2 wagging modes[50,51] redshift from 1145 cm^{-1} at E = 2.9 V_{Li} to 1135 cm^{-1} at E=2.1 V_{Li} (Supplementary Fig. 17)." (Page 10, Line 259)

Comment 11: Line 261: do you mean 292 cm^{-1}?

Response: Thank you very much for this suggestion. This comment has been addressed in our response to Comment 5. As clarified there, the correct peak position is 292 cm^{-1}, and this value has now been consistently revised in both the main text and the Supplementary Information, together with the addition of the enlarged spectral view.

"Additionally, spectral peaks observed at 260 cm^{-1} and 292 cm^{-1} are attributed to the rocking vibrations of S-F and SO_2, respectively. A closer inspection of these bands under positive polarization reveals a two-stage potential dependence (Supplementary Fig. 18). As the potential is increased from 3.0 to 3.3 V_{Li}, the intensities of both the S-F and SO_2 peaks rise, consistent with enhanced accumulation of FSI^- at the electrode interface. Upon further increasing the potential from 3.3 to 3.7 V_{Li}, the S-F peak continues to grow in intensity while the SO_2 peak decreases; concurrently the SO_2 blueshifts from 292 cm^{-1} (E=3.0 V_{Li}) to 298 cm^{-1} (E=3.7 V_{Li}). These observations indicate a potential-driven reorientation of adsorbed FSI^- in which the S-F moiety approaches the electrode surface while the SO_2 group tilts toward the solution. Such reconfiguration promotes a higher surface adsorption density of anions and alleviates interfacial steric hindrance, confirming that the first interfacial layer is dominated by specifically adsorbed FSI^-." (Page 10, Line 265)

Comment 12: Figure 2: this figure (and all of them, actually) is too crowded. Consider

putting some of these into the SI and only leaving here the most relevant information. The term "heatmap" in lines 216 to 217 had me confused. Is Figure 2a a real heatmap or a superposition of all 20 force-distance curves? What do the green-red colours mean? Please clarify.

Response: Thank you very much for this helpful suggestion. Thank you very much for this helpful suggestion. We agree that the original presentation of Fig. 2 (and several other figures) was visually dense, which may have reduced clarity. Following the reviewer's recommendation, we have streamlined the main figures by moving less essential panels to the Supporting Information and retaining only the most relevant information in the main text.

Regarding the use of the term "heatmap", we apologize for the confusion caused by our original wording. In this work, the "heatmap" does not represent a conventional spatial heatmap. Instead, it refers to a two-dimensional force-separation histogram constructed from 20 independent force curves. In these plots, force and separation are treated as the two axes, and the green-to-red color scale represents the statistical density of data points, from low to high occurrence. This representation allows us to visualize the most probable force-separation features and extract characteristic layering distances in a statistically robust manner.

To avoid further ambiguity, in the revised manuscript we have replaced the term "heatmap" with force-separation profiles and have explicitly clarified their construction in the text. The corresponding figures are now clearly indicated as Fig. 2a and Fig. 3a, with an expanded explanation provided in the Methods and Supporting Information.

"To provide an atomistic resolution of the interfacial structure and corroborate the experimental inferences, we performed molecular dynamics simulations using the Constant Potential Method (CPM), as shown in Fig. 2a. The simulation explicitly captures the potential-dependent restructuring of the electrical double layer (EDL) at the graphite-electrolyte interface. At potentials negative relative to the PZC, the simulated density profiles and snapshots reveal that the innermost Helmholtz plane is predominantly occupied by adsorbed free G4 solvent molecules rather than desolvated Li^+ ions. These interfacial G4 molecules preferentially adopt a flattened orientation parallel to the basal plane, forming a dense solvent layer that is consistent with the ~0.32 nm step thickness observed in AFM. The solvated $Li(G4)^+$ cations are statistically located in the second layer, separated from the electrode by this solvent cushion. Conversely, under positive polarization, the simulation shows the accumulation of FSI^- anions co-adsorbed with G4 molecules to screen the positively charged surface (Supplementary Fig. 19). These trends are in well agreement with the potential-dependent interfacial layering resolved by AFM." (Page 11, Line 282)

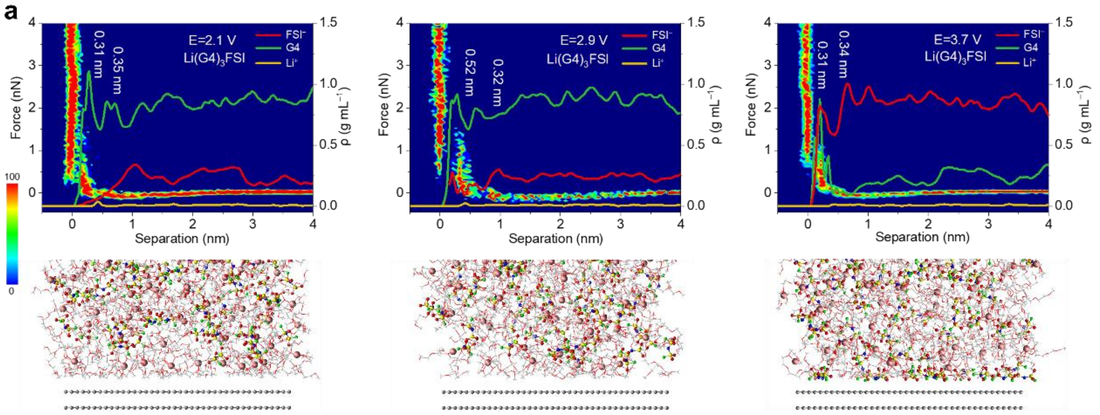

Figure R2–4, **a,** Two-dimensional AFM force-separation histograms from 20 independent force curves for [Li(G4)$_3$][FSI] obtained at potentials of E=2.1 V_{Li}, E=2.9 V_{Li}, and E=3.7 V_{Li} with probability colour bar on the left-hand side and ion number density profiles of FSI^- (red), G4 (green) and Li^+ (yellow) from MD simulations at PZC−1.0 V, PZC, PZC+1.0 V (top row) . Representative snapshots from the simulation at −1.0 V, 0 V and +1.0 V relative to the PZC (bottom row), shown from left to right.

"In contrast to the dilute regime, where free solvent molecules dominate the interface (Fig. 3a), the simulation reveals that in highly-concentrated electrolytes the scarcity of free G4 molecules precludes the formation of a solvent-passivated layer. Instead, the interface exhibits a distinct alternating ion-layering mechanism. At potentials negative of the PZC, the innermost Helmholtz plane is populated primarily by bulky $Li(G4)^+$ cations, corresponding to the larger step thickness (~0.52 nm) detected by AFM. Conversely, under positive polarization, the interface becomes saturated by a compact layer of FSI^- anions, aligning perfectly with the reduced step thickness (~0.32 nm) (Supplementary Fig. 26). These results demonstrate that electrolyte concentration exerts a decisive control over the interfacial nanostructure by shifting the positions of discrete layering steps, reflecting changes in the physical dimensions and composition of ion accumulation near the electrode." (Page 14, Line 363)

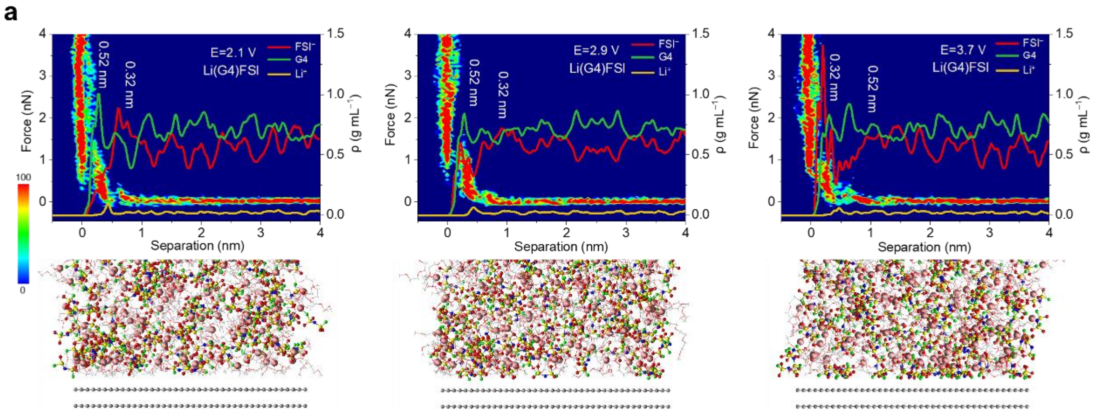

Figure R2–5, **a,** Two-dimensional AFM force-separation histograms from 20 independent force curves for [Li(G4)][FSI] obtained at potentials of E=2.1 V_{Li}, E=2.9 V_{Li}, and E=3.7 V_{Li} with probability colour bar on the left-hand side and ion number density profiles of FSI^- (red), G4 (green) and Li^+ (yellow) from MD simulations at PZC−1.0 V, PZC, PZC+1.0 V (top row) . Representative snapshots from the simulation at −1.0 V, 0 V and +1.0 V relative to the PZC (bottom row), shown from left to right.

Comment 13: Figure 2a: the combination of blue with bright green and red is very bright, please consider changing these colors and improve the font, it looks too pixelated and too small. This is true for the rest of the figure and the other ones as well.
Response: Thank you for this helpful suggestion regarding the figure presentation. We agree that the original color scheme and font size could hinder readability.
In the revised manuscript, we have adjusted the color palette throughout Fig. 2a and the other figures by darkening the background and softening the contrast between the blue, red, and green elements, so that the different components can be clearly distinguished without excessive brightness. In addition, the font size has been increased and the font rendering has been improved to enhance legibility and reduce pixelation.
These modifications have been applied consistently across all figures in the manuscript to ensure a clearer and more visually accessible presentation. We believe that these changes substantially improve the overall readability and visual quality of the figures.

Comment 14: SI Tables 1-2: I believe a plot comparing both would be better for visualization.
Response: Thank you very much for this suggestion. As recommended, we have converted SI Tables 1 and 2 into a comparative plot in the revised Supplementary Information to improve data visualization and facilitate direct comparison.

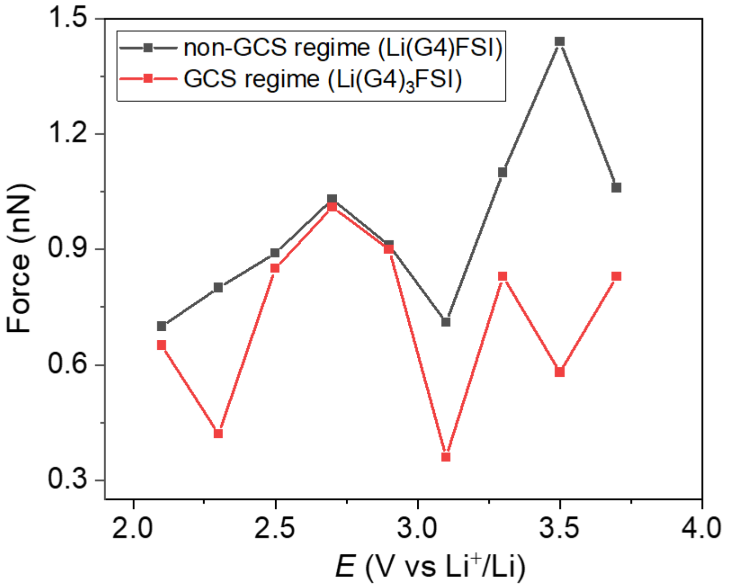

Figure R2–6. The average rupture force of Li(G4)FSI and Li(G4)$_3$FSI under different electrode potentials. (SI, Page 33)

Comment 15: Line 384: this equation looks to ackward inline, consider having it on its own.

Response: Thank you very much for this suggestion. We have reformatted the equation in Line 449 and placed it as a standalone displayed equation in the revised manuscript to improve readability.

"To quantify the concentration-dependent evolution of solvation entropy, we employed the two-body excess entropy (S_2) approximation derived from liquid-state statistical mechanics. This framework captures the entropy contribution arising from pairwise spatial correlations and has been widely used to characterize structural ordering in complex fluids and ionic systems[57,58]. Specifically, the solvation entropy associated with Li^+ was evaluated from the radial distribution functions between Li^+ and surrounding species, according to

$$S_2 = -2\pi\rho\, k_B \int_0^\infty [g(r)\ln g(r) - g(r) + 1]\, r^2\, dr,$$

where $g(r)$ is the radial distribution function, ρ is the number density, and k_B is the Boltzmann constant." (Page 17, Line 435)

Comment 16: Supplementary Figures 2 to 6: please improve them, the text is barely visible.

Response: Thank you very much for this suggestion. We have improved the quality of Supplementary Figures 2–6 in the revised Supporting Information by enlarging all text to ensure they are clearly readable.

Figure R2–7. (SI, Page 7–11)

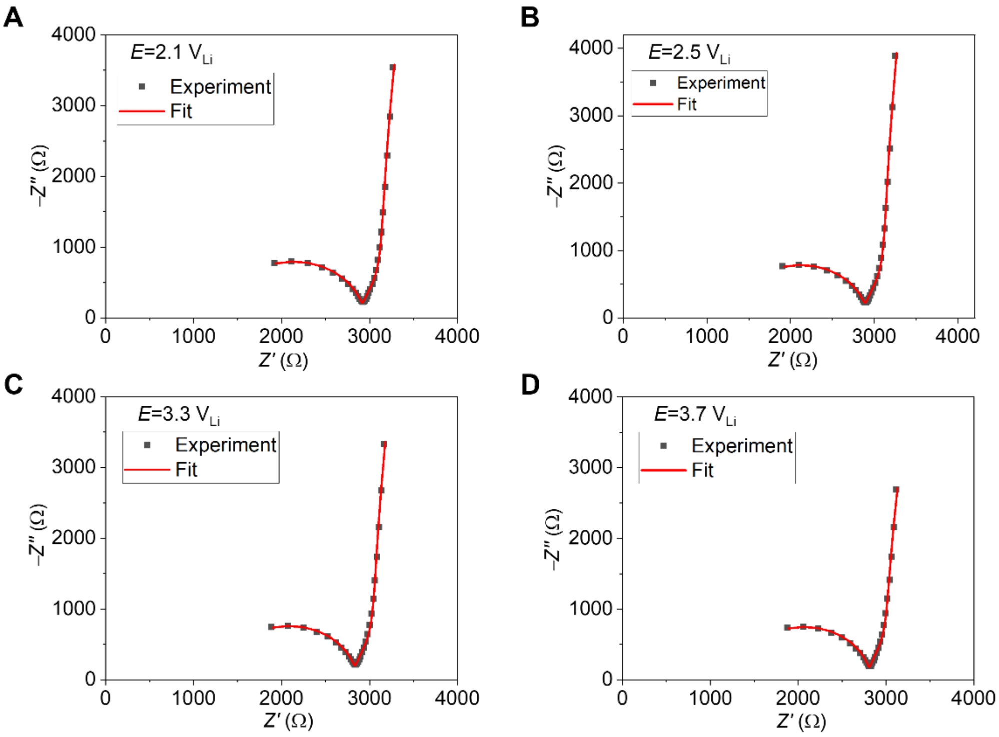

Supplementary Fig. 2. Comparison of fitted curves and experimental data for Li(G4)FSI on a HOPG electrode at different potentials.

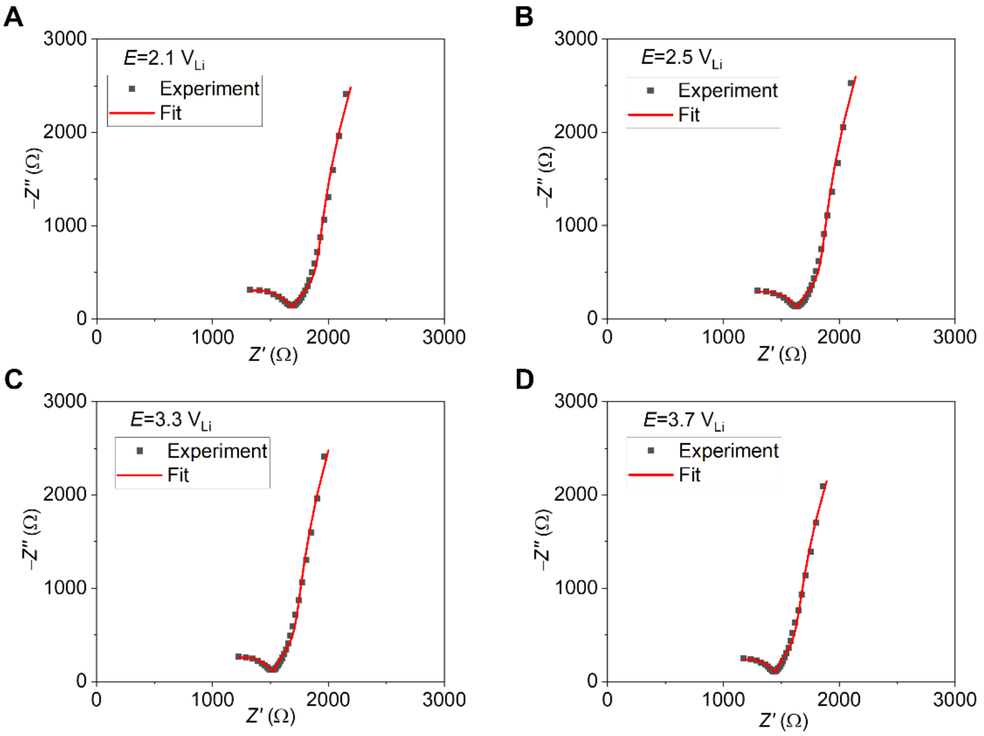

Supplementary Fig. 3. Comparison of fitted curves and experimental data for $Li(G4)_3FSI$ on a HOPG electrode at different potentials.

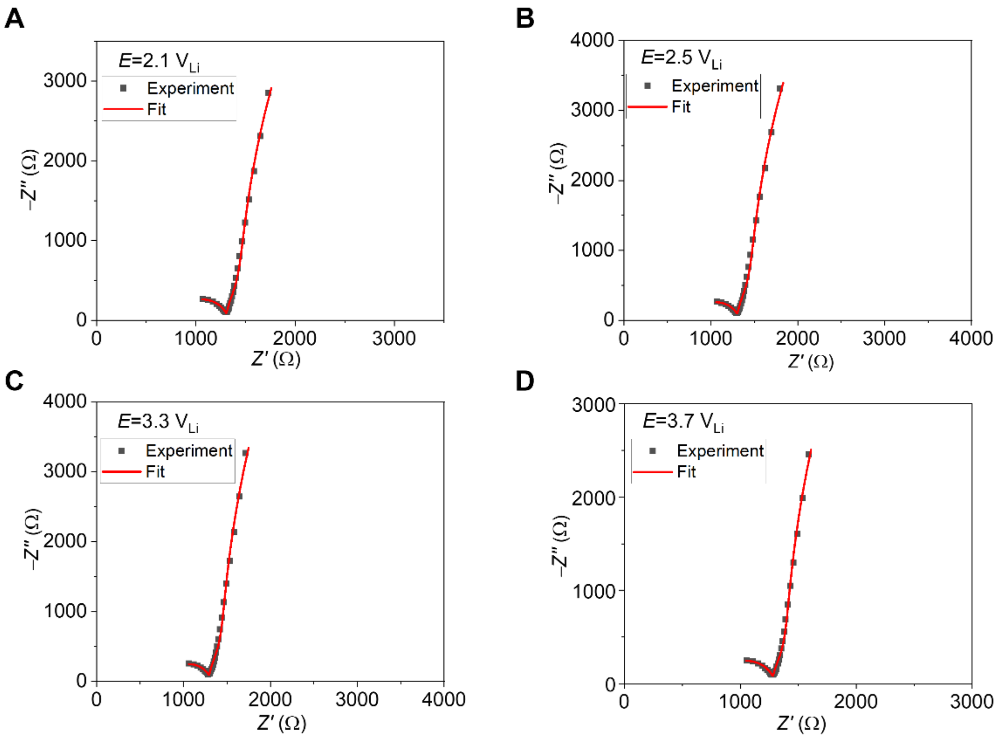

Supplementary Fig. 4. Comparison of fitted curves and experimental data for Li(G4)$_5$FSI on a HOPG electrode at different potentials.

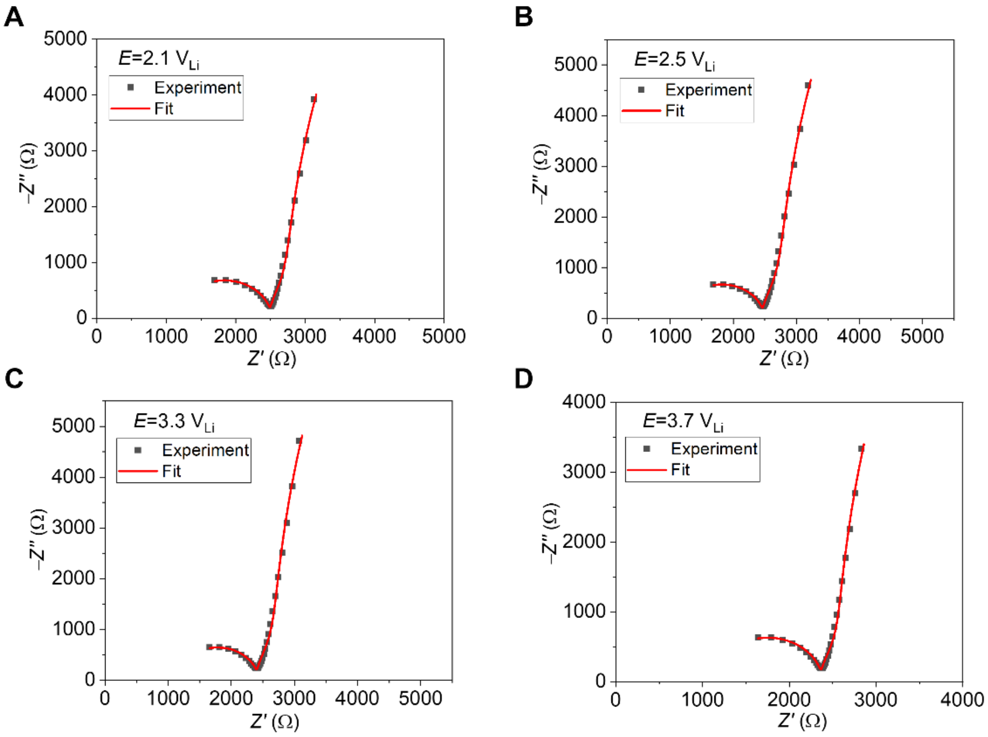

Supplementary Fig. 5. Comparison of fitted curves and experimental data for Li(G4)$_{10}$FSI on a HOPG electrode at different potentials.

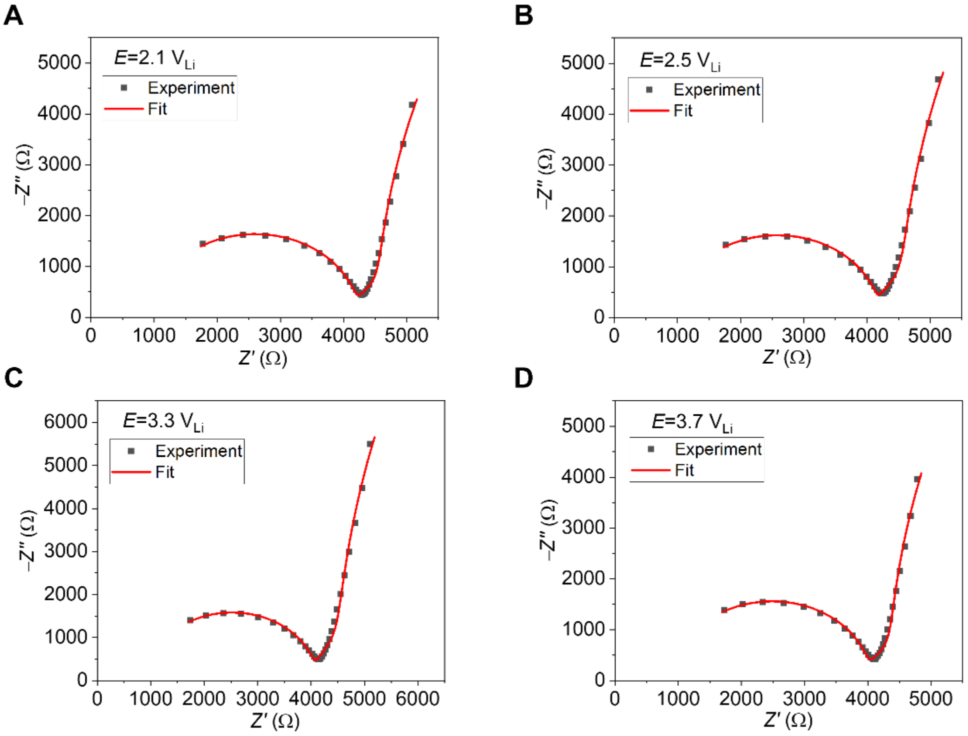

Supplementary Fig. 6. Comparison of fitted curves and experimental data for $Li(G4)_{20}FSI$ on a HOPG electrode at different potentials.

Reviewer #3 (Remarks to the Author):
In this work, the authors have conducted in situ gap-enhanced Raman spectroscopy to probe the electrical double layer (EDL) of the the lithium battery electrolytes composed of LiFSI and G4 with various concentrations, namely Li(G4)3FSI and Li(G4)FSI. Machine-learning molecular dynamics (MLMD) have also been performed to demonstrate the solvation structures and dynamics of lithium ions in the Li(G4)3FSI and Li(G4)FSI. Despite the efforts made in this work, I suggest rejection of this manuscript for publication in the prestigious journal of Nature communications due to the following concerns:
Response: We thank the reviewer for the constructive feedback on our work. Below, we provide a point-by-point response to each specific comment and clarify the scientific basis of our conclusions.
Regarding the concern about anomalous screening, we would like to emphasize that the phenomenon discussed in our work is fundamentally different from the long-range underscreening reported by Gebbie et al. In those studies, underscreening lengths (typically exceeding 10 nm) were attributed to ion clustering and an extremely low population of free ions in room-temperature ionic liquids. In contrast, the anomalous screening observed here is strictly confined to the electrode-electrolyte interface of lithium-ion battery electrolytes and occurs on a nanometer length scale (< 2 nm). Our combined AFM, Raman, and molecular dynamics results consistently indicate that this behavior originates from the reorganization of Li^+ solvation structures and the enhanced participation of anions within the solvation sheath under high salt concentrations. This distinction is now made explicit in the revised manuscript to avoid conceptual ambiguity.
Regarding the concern that dynamic exchange phenomena were inferred solely from bulk simulations, we have now performed additional classical molecular dynamics simulations using the constant potential method (CPM) to explicitly model the electrode-electrolyte interface. These interfacial simulations demonstrate that the concentration-dependent trends in solvation-sheath exchange observed in the bulk are preserved and, in fact, become more pronounced within the electrical double layer. This directly supports our interpretation that the reported dynamic exchange phenomena are intrinsic to the EDL.
Regarding the physical basis of the EDL schematic in Fig. 2a, we have carried out dedicated classical molecular dynamics simulations of the electrolyte in contact with a graphite surface. The resulting number density profiles clearly reveal the formation of well-defined interfacial solvation layers near the carbon surface. Importantly, the thickness of these layers quantitatively agrees with the “steps” observed in the AFM force curves. The simulations further identify the specific species occupying the inner Helmholtz plane, providing direct structural evidence that supports the spectral assignments observed in the in situ gap-enhanced Raman measurements.
Taken together, these additional interfacial simulations, combined with the

experimental AFM and Raman results, establish a consistent and physically grounded picture of the electrical double layer structure in lithium battery electrolytes. We hope that these detailed responses and the corresponding revisions address the reviewer's concerns.

Comment 1: The authors claimed they have revealed the anomalous screening that is beyond the GCS theory, which has been reported by many previous works (PNAS, 2023, 110, 9674-9679; PNAS, 2015, 7432-7437) in some electrolyte systems, not to mention that the concept of anomalous screening still remains under strong debate (Electrochemistry Communications, 2017, 82, 129-133; https://arxiv.org/abs/2408.15685). I do not see new finding in this work in terms of the so-called anomalous screening of the concentrated electrolytes.

Response: Thank you for this comment. Previous studies have reported the underscreening phenomenon observed in surface force apparatus (SFA) experiments, including those reported in PNAS (PNAS, 2013, 110, 9674-9679; PNAS, 2015, 112, 7432-7437). However, it is important to clarify that the anomalous screening discussed in our work is fundamentally different from the underscreening regime reported by Gebbie et al. In those studies, the exceptionally long screening lengths (often exceeding 10 nm) were attributed to ionic clustering and an extremely low population of free ions in room-temperature ionic liquids. In contrast, the anomalous screening reported in our work occurs at the lithium-ion battery electrolyte interface, on a strictly nanometer scale (< 2 nm), and is directly associated with the reconstruction of Li^+ solvation structures and enhanced anion participation within the solvation sheath. Our AFM, Raman spectroscopy, and molecular dynamics results consistently show that, with increasing salt concentration, the effective decay length of interfacial charge interactions deviates from Debye–Hückel predictions in a non-monotonic manner, rather than exhibiting dramatic long-range underscreening. This distinction is critical, and we have clarified it explicitly in the revised manuscript to avoid possible confusion.

Regarding the ongoing debate on underscreening, recent studies indeed suggest that the phenomenon may arise from multiple decay modes or even experimental artifacts specific to certain SFA measurements (https://arxiv.org/abs/2408.15685; Chemical Physics Letters, 2024, 843, 141190). In this context, Kornyshev et al have explicitly emphasized that the interpretation of long-range screening lengths in ionic liquid systems is highly sensitive to the underlying theoretical assumptions and experimental analysis, and have cautioned against treating underscreening as a universal or intrinsic property of electrical double layers (Electrochemistry Communications, 2017, 82, 129–133). Importantly, our work does not rely on the underscreening interpretation at all. Instead, we establish a direct, experimentally validated link between solvation entropy, anion-enriched solvation structures, dynamic ion exchange at the interface, and the resulting deviation from classical GCS behavior under practical battery electrolyte conditions. To the best of our

knowledge, such a thermodynamic-kinetic unified picture for anomalous interfacial screening in highly concentrated lithium battery electrolytes has not been previously reported. Therefore, while underscreening as a general concept has indeed been discussed before, the physical origin, electrolyte system, spatial scale, and interfacial implications revealed in our work are fundamentally distinct and constitute the key novelty of this study.

"As a summary of EIS results, we observe a pronounced deviation from classical Gouy−Chapman−Stern screening behavior in highly-concentrated solutions. Instead of decreasing monotonically with increasing salt concentration, the effective decay length of interfacial charge screening exhibits a nonmonotonic dependence on concentration. This anomalous screening likely originates from concentration-induced reorganization of the local solvation structure and ion correlations, rather than from conventional long-range electrostatic underscreening." (Page 7, Line 189)

Comment 2: The authors also claimed they have revealed dynamic exchange phenomena in the EDL across varying salt concentrations. However, the related conclusion was made based on the solvation shell exchange of the bulk solutions with different salt concentrations by MLMD simulations, which has nothing to do with the EDL. There is no doubt there will be more anion that enters into the Li ion solvation shell with increasing salt concentration.

Response: We thank the reviewer for this critical and insightful comment. We fully agree that the behavior in the bulk solution does not automatically translate to the electrical double layer (EDL), and that explicit interfacial simulations are necessary to substantiate our claims regarding the dynamic exchange phenomena in the EDL. In the original manuscript, our primary motivation for using MLMD on bulk systems was to leverage their ab initio accuracy to rigorously quantify the intrinsic rigidity of the solvation sheath. The MLMD results provided a thermodynamic basis for our hypothesis, revealing that the solvation structure in low-concentration electrolytes exhibits higher rigidity, whereas the anion-rich solvation environment in highly concentrated electrolytes is structurally "looser" and more dynamic.

To directly address the reviewer's concern and bridge the gap between bulk properties and EDL behavior, we have now performed additional Classical Molecular Dynamics simulations using the Constant Potential Method (CPM). These new simulations allow us to explicitly model the interface under realistic electrode polarization conditions (ranging from −1 V to 1 V vs. PZC). The new interfacial simulation results (now added as Figure 2a and 3a in the revised manuscript) clearly demonstrate that the trend observed in the bulk is preserved and even amplified within the EDL. Under polarization, the exchange frequency between Li^+ and anions in the high-concentration system is significantly higher than that in the dilute system. The CPM results confirm that the "looser" solvation sheath identified by MLMD facilitates easier desolvation and reorganization at the interface, directly supporting the mechanism of enhanced

reversibility. Therefore, by combining the high-accuracy intrinsic insights from MLMD with the explicit interfacial description from constant potential CMD, we can now confidently conclude that the dynamic solvation effects are operative within the EDL. We have revised the text in the "Results and Discussion" section (Page 11, Line 282 and Page 14, Line 362) to clearly distinguish between bulk and interfacial simulations and to discuss these new findings.

"To provide an atomistic resolution of the interfacial structure and corroborate the experimental inferences, we performed molecular dynamics simulations using the Constant Potential Method (CPM), as shown in Fig. 2a. The simulation explicitly captures the potential-dependent restructuring of the electrical double layer (EDL) at the graphite-electrolyte interface. At potentials negative relative to the PZC, the simulated density profiles and snapshots reveal that the innermost Helmholtz plane is predominantly occupied by adsorbed free G4 solvent molecules rather than desolvated Li^+ ions. These interfacial G4 molecules preferentially adopt a flattened orientation parallel to the basal plane, forming a dense solvent layer that is consistent with the ~0.32 nm step thickness observed in AFM. The solvated $Li(G4)^+$ cations are statistically located in the second layer, separated from the electrode by this solvent cushion. Conversely, under positive polarization, the simulation shows the accumulation of FSI^- anions co-adsorbed with G4 molecules to screen the positively charged surface (Supplementary Fig. 19). These trends are in well agreement with the potential-dependent interfacial layering resolved by AFM. Beyond structural layering, the simulations further provide molecular-level insight into the solvation structures probed by Raman spectroscopy. The coordination of Li^+ with solvent molecules gives rise to distinct solvation structures including SSIP, CIP and AGG, which can be identified through Raman spectral peaks[53-55]. Consistent with the Raman analysis in Fig. 2b-d, the EDL is dominated by SSIP and free G4 molecules, with only a minor contribution from CIP and a negligible population of AGG. In particular, in the $[Li(G4)_3][FSI]$ electrolyte, AGG are virtually absent at the interface (Fig. 2e), reflecting a relatively loose solvation sheath. At the PZC, the simulated fractions of SSIP, CIP, and AGG quantitatively agree with the experimentally extracted values within ~10% (Fig. 2f), offering a clear physical explanation for the distinct structural ordering and layer compositions detected by *in situ* spectroscopic observations." (Page 11, Line 282)

"In contrast to the dilute regime, where free solvent molecules dominate the interface (Fig. 3a and Supplementary Fig. 26), the simulation reveals that in highly-concentrated electrolytes the scarcity of free G4 molecules precludes the formation of a solvent-passivated layer. Instead, the interface exhibits a distinct alternating ion-layering mechanism. At potentials negative of the PZC, the innermost Helmholtz plane is populated primarily by bulky $Li(G4)^+$ cations, corresponding to the larger step thickness (~0.52 nm) detected by AFM. Conversely, under positive polarization, the interface becomes saturated by a compact layer of FSI^- anions, aligning perfectly with the reduced step thickness

(~0.32 nm) (Supplementary Fig. 27). These results demonstrate that electrolyte concentration exerts a decisive control over the interfacial nanostructure by shifting the positions of discrete layering steps, reflecting changes in the physical dimensions and composition of ion accumulation near the electrode. This concentration-dependent structural reorganization is further manifested in the mechanical response of the EDL. Specifically, comparison of rupture forces measured in [Li(G4)][FSI] and $[Li(G4)_3][FSI]$ (Supplementary Fig. 28) shows that the non-GCS regime sustains larger rupture forces than the classical GCS regime. This observation indicates the formation of a more ordered layered structure within the EDL, exhibiting an anomalous screening length. Such an “ion-crowding” effect, accurately reproduced by the CPM simulation, explains the enhanced rupture forces and the pronounced, potential-driven interfacial restructuring. By contrast, in electrolytes containing excess free solvent, anions are largely excluded from the Li^+ solvation sheath and do not directly participate in the innermost interfacial layer. Together, these findings clarify how the solvation state of lithium ions and the availability of free solvent dictate the nanoscale organization of the EDL, with direct consequences for interfacial electrochemical reactions. In particular, during solid electrolyte interphase (SEI) formation on the anode, the EDL structure plays a decisive role in steering competing reduction pathways, as the spatial proximity of electrolyte components within the EDL governs their likelihood of incorporation into the SEI. Bent and co-workers demonstrated that species positioned closer to the electrode are preferentially incorporated into the SEI during electrolyte decomposition[58]” (Page 14, Line 363)

Comment 3: The schematic for the EDL structure as presented in Figure 2a does not make sense. For example, in the case of E=2.1 V and E=2.5 V (below pzc), some adsorbed Li^+ ions on top of the negatively charged electrode surface would be expected, however, I do not see any of them in the picture. Is there any physical explanation for this? How did the authors conduct the measurement of the EDL, and how did the authors know the exact EDL structures only from the experimental measurement? MD simulations of EDL will be necessary to give deep insights of the EDL structures like it has been done in previous works (Acc. Chemem. Res., 2017, 50, 2886–2894; Energy Environ. Sci., 2025, 18, 3036).

Response: Thank you for this comment. We clarify below the physical basis of the interfacial structure and the purpose of the schematic representation shown in Fig. 2a.

From a physical chemistry perspective, the absence of bare Li^+ ions directly adsorbed on the negatively charged electrode at E = 2.1 V and 2.5 V is consistent with the known solvation characteristics of the LiFSI-glyme system. Tetraglyme (G4) is a multidentate ether solvent that coordinates strongly with Li^+ ions, and a single G4 molecule can saturate multiple coordination sites of Li^+. In contrast, the Li^+–FSI^- interaction is substantially weaker than the Li^+–G4 interaction, as established in previous studies (J. Phys. Chem. C 2011, 115,

18384–18394; Phys. Chem. Chem. Phys. 2020, 22, 525–535). In $Li(G4)_3FSI$, a substantial population of free solvent molecules exists, and the diffusion coefficient of free G4 is approximately 2.5 times higher than that of solvated Li^+ complexes. Consequently, upon changes in the interfacial electric field (e.g., at E = 2.1 V), free solvent molecules are expected to respond more rapidly and preferentially populate the electrode surface, followed by the more sluggish solvated cation species. At these potentials, which are well below the onset of electrolyte decomposition, Li^+ desolvation is energetically unfavorable, and direct adsorption of bare Li^+ onto the negatively charged electrode is therefore not expected.

This behavior is fully consistent with the classical picture proposed by Bockris, Devanathan, and Müller (BDM), who emphasized that an electrode may be regarded as a "giant ion" whose primary solvation shell is formed by solvent molecules, provided sufficient solvent is available. In this framework, the inner Helmholtz plane (IHP) corresponds to the first solvent layer rather than direct counter-ion adsorption (Proc. R. Soc. Lond. A, 1963, 274, 55–79).

In response to the reviewer's suggestion for atomistic insight into the EDL structure, we have now performed additional molecular dynamics simulations of the electrolyte-graphite interface using the Constant Potential Method (CPM) in combination with the OPLS-AA force field. By maintaining the electrode at fixed potentials corresponding to the experimental conditions (0 V, ±0.5 V, and ±1.0 V versus PZC), these simulations explicitly capture electrode polarization and interfacial charge redistribution. The resulting ion and solvent number density profiles confirm that the inner Helmholtz plane is predominantly occupied by free G4 solvent molecules rather than desolvated Li^+ ions, in excellent agreement with the AFM force measurements and the schematic representation in Fig. 2a.

"To provide an atomistic resolution of the interfacial structure and corroborate the experimental inferences, we performed molecular dynamics simulations using the Constant Potential Method (CPM), as shown in Fig. 2a. The simulation explicitly captures the potential-dependent restructuring of the electrical double layer (EDL) at the graphite-electrolyte interface. At potentials negative relative to the PZC, the simulated density profiles and snapshots reveal that the innermost Helmholtz plane is predominantly occupied by adsorbed free G4 solvent molecules rather than desolvated Li^+ ions. These interfacial G4 molecules preferentially adopt a flattened orientation parallel to the basal plane, forming a dense solvent layer that is consistent with the ~0.32 nm step thickness observed in AFM. The solvated $Li(G4)^+$ cations are statistically located in the second layer, separated from the electrode by this solvent cushion. Conversely, under positive polarization, the simulation shows the accumulation of FSI^- anions co-adsorbed with G4 molecules to screen the positively charged surface (Supplementary Fig. 19). These trends are in well agreement with the potential-dependent interfacial layering resolved by AFM." (Page 11, Line 282)

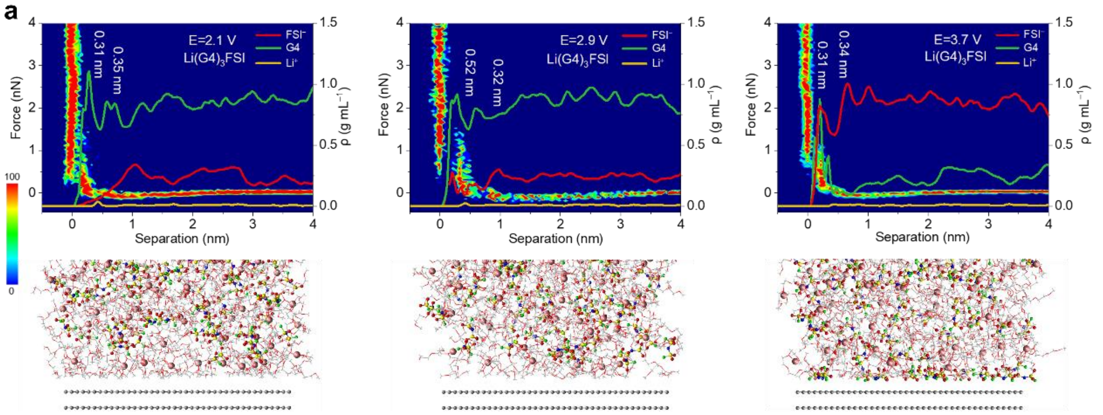

Figure R3–1, **a,** Two-dimensional AFM force-separation histograms from 20 independent force curves for $[Li(G4)_3][FSI]$ obtained at potentials of E=2.1 V_{Li}, E=2.9 V_{Li}, and E=3.7 V_{Li} with probability colour bar on the left-hand side and ion number density profiles of FSI^- (red), G4 (green) and Li^+ (yellow) from MD simulations at PZC−1.0 V, PZC, PZC+1.0 V (top row). Representative snapshots from the simulation at −1.0 V, 0 V and +1.0 V relative to the PZC (bottom row), shown from left to right.

Reviewer #4 (Remarks to the Author):

In this manuscript, Yin et al. report a study on the electrochemical double-layer that forms when Li salt concentrated electrolytes are put in contact with graphite electrode. It combines electrochemistry measurements (impedance and cyclic voltammetry) with atomic force microscopy, surface-enhanced Raman spectroscopy and molecular dynamics simulations. Although the approach and the systems are interesting, I think that there are many inconsistencies that undermine the conclusions as well as the originality of the work. Consequently, I do not recommend publication in Nature communications and I recommend the authors to address the following points before resubmitting the manuscript to any journal.

Response: We thank the reviewer for the careful evaluation of our manuscript and for acknowledging the interest of both the approach and the systems studied. We have carefully considered all concerns raised and addressed them point by point in the revised manuscript.

In particular, the issues of consistency and originality highlighted by the reviewer have prompted us to substantially clarify the physical interpretation of our results and to better contextualize them within the existing literature. We believe that several of the perceived inconsistencies arose from insufficient explanation rather than from fundamental limitations of the experimental data or their analysis.

To further strengthen the robustness of our conclusions, we have now incorporated additional interfacial simulations, which are fully consistent with and complementary to the experimental observations. These new results provide independent support for the proposed interfacial mechanisms and help bridge the gap between experimental observables and microscopic interpretation.

We believe that these revisions and the detailed responses provided below directly address the reviewer's concerns, significantly improve the clarity and coherence of the manuscript. We respectfully hope that the revised manuscript may now be considered suitable for publication.

Comment 1: The main result from which the authors conclude on an "anomalous screening" is based on the Parsons-Zobel plots shown on Figure 1a and 1b. However, contrarily to the authors claim (page 5, line 129, they state that it remains an open question), it is well established that the Gouy-Chapman-Stern theory does not work for concentrated electrolytes, from a vast literature of theory, simulation and experimental results. It is therefore not relevant to calculate the corresponding capacitance for the present systems. The results are compared to previous studies on diluted electrolytes (for example the green dots on Figure 1a were obtained for concentrations up to 5mM only), for which it was much more meaningful. In addition, the double-layer capacitance arises from a fit of the impedance spectroscopy using a six-parameter equivalent circuit model. The impedance spectra shown in the SI do not seem very different from one concentration to another, and it is possible that there are some error compensations arising from the fit. Normally, some of the parameters should remain

constant from one system to another but the authors do not provide the results of the fit so it is difficult to check this.

Response: Thank you very much for this comment. We agree that the classical Gouy-Chapman-Stern (GCS) model is not applicable to highly-concentrated electrolytes, and we regret that our original wording may have caused ambiguity. Our intention was not to use the GCS framework to interpret the EDL structure of highly-concentrated systems. Instead, the Parsons−Zobel (PZ) representation was used purely as a reference framework to visualize how the experimentally measured double-layer capacitance departs from classical behavior. This approach is consistent with the methodology used in prior foundational studies on non-classical EDL behavior, including those by Koper and co-workers (Angew. Chem. Int. Ed. 2020, 59, 711–715; Proc. Natl. Acad. Sci. U.S.A. 2022, 119, e2116016119), in which PZ representations are explicitly used to highlight deviations of real interfaces from the classical dilute-solution. Our comparison with low-concentration literature data (e.g., the 5 mM systems) therefore serves not as a mechanistic application of GCS, but as a contrast benchmark that reveals the onset of non-classical screening as the salt concentration increases. This deviation relative to the dilute baseline is the key observation, rather than the absolute applicability of the GCS model itself.

Regarding the impedance analysis, we performed a direct comparison between a generic equivalent circuit with ambiguous physical meaning (Figure R4–1, A) and the equivalent-circuit model in which each element has a clear physical assignment used in our manuscript (Figure R4–1, B). Both models were used to the same EIS dataset at a fixed potential for each electrolyte. The physically meaningful model proposed in our work yields consistently small fitting errors, well-defined parameter values, and no sign of cross-parameter compensation across all electrolyte concentrations. Importantly, the same circuit topology provides stable fits for every concentration measured (Supplementary Figures 2–6), demonstrating that the extracted double-layer capacitance is not an artifact of over-parameterization. These results confirm that the capacitance trends reported in the main text originate from systematic electrochemical behavior rather than from fitting degeneracy.

"The EIS data were interpreted using the equivalent circuit model shown in Supplementary Fig. 1, in which the high-frequency semicircle corresponds to bulk solution, the intermediate-frequency semicircle represents processes associated with chemisorption and double layer charging, and the low-frequency semicircle is attributed to side reactions at the interface[32-34]." (Page 5, Line 140)

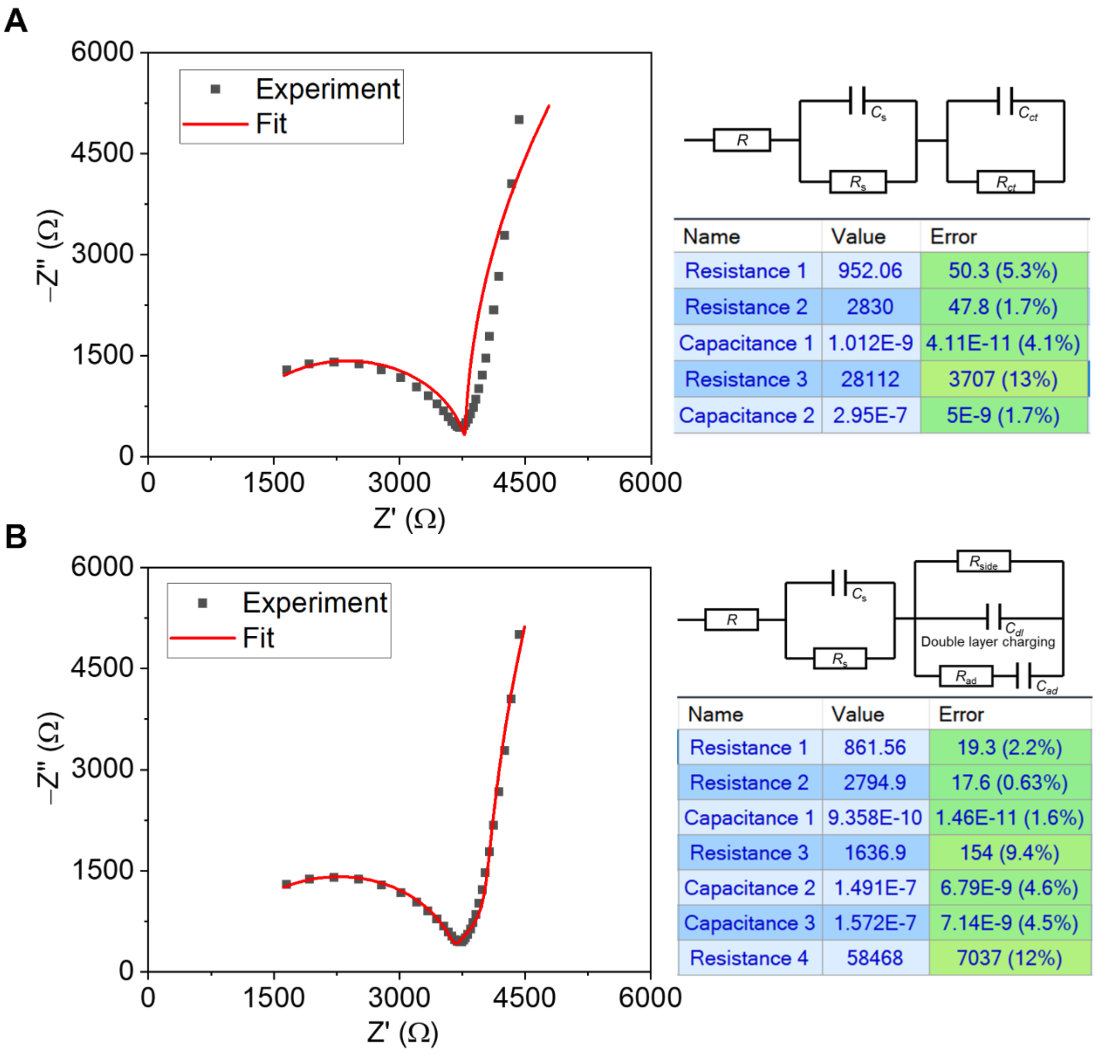

Name	Value	Error
Resistance 1	952.06	50.3 (5.3%)
Resistance 2	2830	47.8 (1.7%)
Capacitance 1	1.012E-9	4.11E-11 (4.1%)
Resistance 3	28112	3707 (13%)
Capacitance 2	2.95E-7	5E-9 (1.7%)

Name	Value	Error
Resistance 1	861.56	19.3 (2.2%)
Resistance 2	2794.9	17.6 (0.63%)
Capacitance 1	9.358E-10	1.46E-11 (1.6%)
Resistance 3	1636.9	154 (9.4%)
Capacitance 2	1.491E-7	6.79E-9 (4.6%)
Capacitance 3	1.572E-7	7.14E-9 (4.5%)
Resistance 4	58468	7037 (12%)

Figure R4−1, Comparison of two equivalent-circuit models used for fitting the impedance spectra. (**A**) A generic phenomenological circuit with ambiguous physical interpretation, in which the circuit elements do not correspond directly to well-defined interfacial processes. (**B**) The physically motivated equivalent-circuit model employed in this work, where each element has a clear physical assignment associated with chemisorption and double layer charging. The EIS data were analyzed using RelaxIS (version 3.0.15, rhd Instruments GmbH & Co. KG, Darmstadt, Germany).

Comment 2: The authors mention that "However, most studies treat solvation behavior and intercalation processes independently, overlooking their mutual influence", suggesting that it is not the case in the present study. However, most of the analysis are made in the absence of intercalation so the approach is not very different from previous works.

Response: Thank you very much for this comment. We agree that our original wording may have created ambiguity regarding how solvation behavior and intercalation are connected in this study, and we would like to clarify this point more explicitly.

First, the majority of our interfacial characterizations—including in-situ gap-enhanced Raman spectroscopy, EC-AFM, and EIS—were intentionally performed under non-intercalation, potentiostatic conditions. We designed the experiments this way because once significant lithium intercalation occurs, the electrode surface rapidly develops or evolves a solid electrolyte interphase (SEI), which is ion-permeable but electronically insulating, thereby obscuring direct measurement of the potential-dependent EDL structure. By avoiding concurrent intercalation during interface probing, we are able to reveal the intrinsic potential-dependent solvation structures and layering features at the electrode/electrolyte interface.

Second, our key conclusion is not that we directly measured solvation sheath during the intercalation process. Rather, our conclusion arises from two independent but mutually consistent sets of evidence: (1) Interfacial measurements: Our in-situ Raman data resolve clear potential-dependent changes among the three Li^+ solvation configurations (SSIP, CIP, AGG) at the interface; EC-AFM provides corresponding structural signatures; and EIS/CV reveal the electrochemical response of the interface. (2) Electrochemical cycling: Independent galvanostatic cycling tests show a strong concentration-dependent reversibility of graphite intercalation. Importantly, these trends correlate directly with the interface solvation environment obtained from the above interfacial analyses.

Together, these complementary observations support the mechanistic picture that highly concentrated electrolytes promote anion-rich solvation environments near the interface. This shifts the electrolyte reduction pathway and yields an SEI dominated by anion-derived species, which is known to strongly influence Li^+ desolvation and subsequent intercalation kinetics. Thus, although our interface measurements were not conducted during active intercalation, they provide the structural and chemical basis that explains the concentration-dependent intercalation reversibility observed in the independent cycling tests.

Comment 3: The analysis of the AFM results is not very clear. The authors conclude on the composition of the first layer based on the obtained layer thickness, but they do not provide the geometric properties of the ions / diglyme in the system so it is not easy to follow. It would be good to provide a plot in which all the concentrations are shown, as in the current form all the plots are separated in the SI. It would also be good to explain how precise are the measurements and the analysis. For example, when I look at Figure 2.a, the middle panel looks to me very similar to the other ones so why are the two thicknesses inverted?

Response: We thank the reviewer for this detailed and constructive comment, which helped us substantially improve the clarity and rigor of the AFM analysis. To clarify the interpretation of the AFM-derived layer thicknesses, we now provide ion and solvent number density profiles obtained from molecular dynamics simulations employing the constant potential method (CPM). These profiles describe the statistically averaged interfacial layering along the surface-

normal direction and are therefore directly comparable to the ensemble-averaged structures sensed by AFM force-separation measurements. In multicomponent electrolytes, the effective interfacial layer thickness is determined by collective solvation and ion-ion correlation effects rather than by the geometric size of a single molecular species. The simulated density profiles show quantitative agreement with the step positions and widths observed in the AFM two-dimensional force-separation histograms, providing a clear structural basis for the AFM interpretation.

To facilitate a direct comparison across electrolyte concentrations, we have added a consolidated plot in the revised Supporting Information (Fig. S27), where the extracted layering thicknesses for both concentrations are presented together rather than in separate figures. Representative AFM force-separation curves are also shown to explicitly illustrate how the separation distance is defined and extracted, enabling a unified visualization of the potential- and concentration-dependent evolution of the interfacial layering. (Figure R4-2)

Concerning the reviewer’s observation that the middle panel of Fig. 2a appears visually similar to the neighboring panels, we note that the interpretation is based on the two-dimensional force-separation histograms. The inversion of the characteristic step thickness reflects a reorganization of the dominant interfacial species as the electrode potential is varied. This conclusion is independently supported by CPM-based molecular dynamics simulations.

We believe that these additions and clarifications significantly improve the transparency and robustness of the AFM analysis.

“Additionally, molecular dynamics simulations with Constant Potential Method (CPM) were conducted to complement and corroborate our experimental observations.” (Page 8, Line 212)

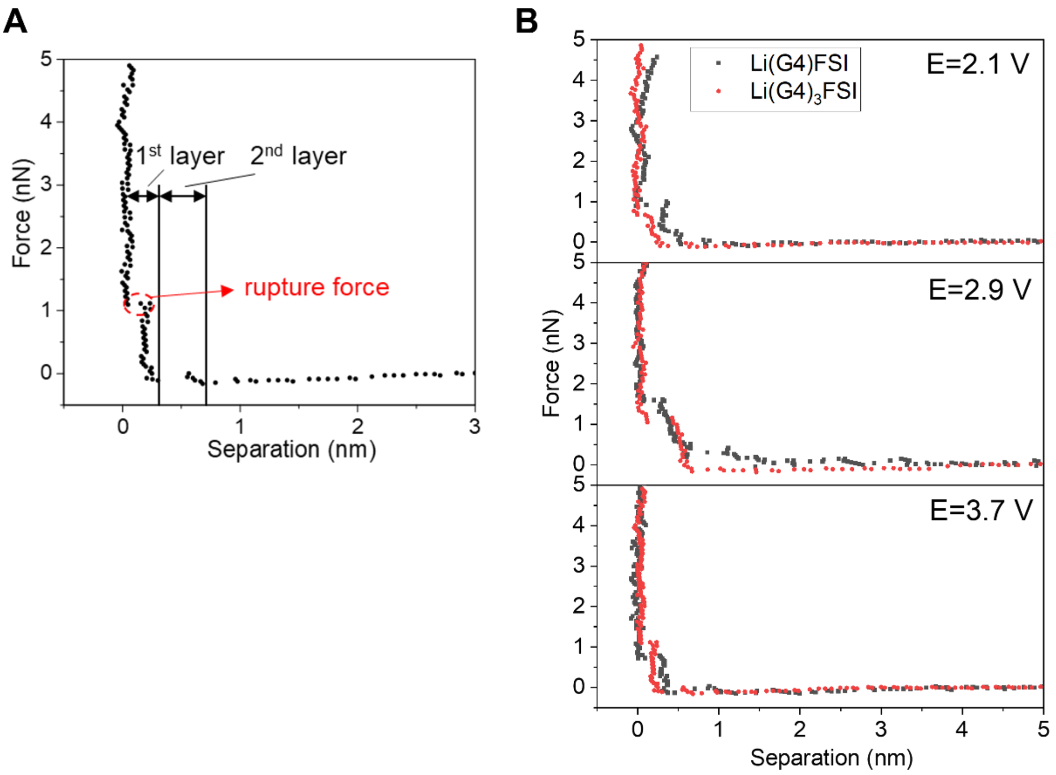

Figure R4−2. (**A**), Example of a representative force-separation curve. (**B**), Force curves at different potentials for two electrolyte concentrations (E < PZC, E ≈ PZC, and E > PZC). Black: Li(G4)FSI, Red: Li(G4)$_3$FSI. (SI, Page 32)

Comment 4: I do not understand from which simulations do the results on Figure 3g come. From the methods section it looks like the authors only performed MD simulations of bulk liquids, so where does the PZC come from here?

Response: Thank you very much for this comment. We apologize for the confusion and thank the reviewer for this observation. The reviewer is correct that the MLMD simulations described in the original manuscript focused on the bulk solvation structure and could not directly yield interfacial properties like the PZC. To address this limitation and provide a rigorous calculation of the PZC, we have incorporated Classical Molecular Dynamics simulations using the Constant Potential Method (CPM) in the revised manuscript. The results presented in Figure 3g (in the revised version) are now explicitly derived from these new CPM simulations, which allow us to simulate the open-circuit conditions and determine the electrode potential at zero surface charge density. We have also updated the Methods section to include a detailed description of the CPM simulation setup and parameters.

"Furthermore, the CPM simulation captures a high degree of ionic correlation within the double layer; unlike the diffuse distribution in dilute systems, the interfacial species in the high-concentration electrolyte regime exist predominantly as CIP and AGG." (Page 14, Line 359)

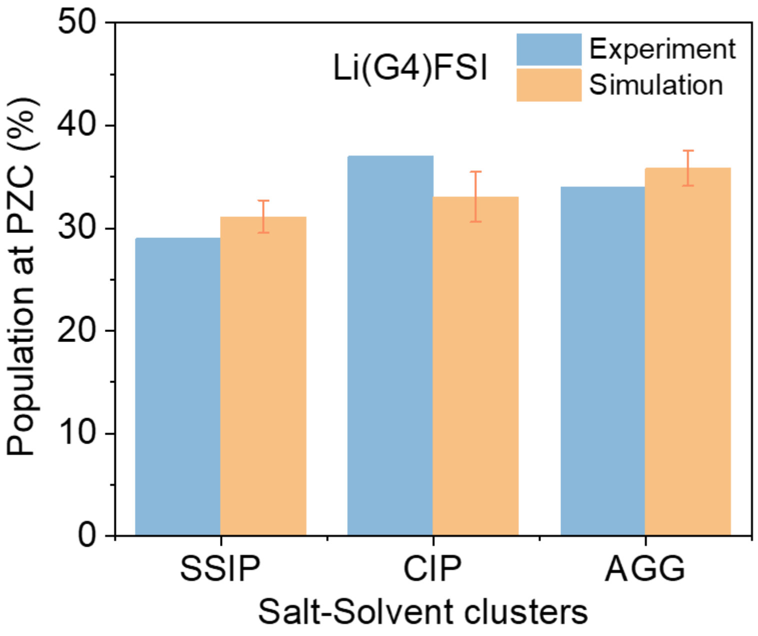

Figure R4−3. **g**, Comparison of interfacial structural characteristics at E=0.0 V obtained from experiment (blue) and simulations (orange). Simulation data represent averages over 200 independent samples, with error bars indicating statistical uncertainty. Minor discrepancies can be attributed to limitations of the OPLS/AA force field and residual uncertainties in both experiment and simulation.

Comment 5: Following the previous questions, did the authors consider simulating the electrolytes at the interface with carbon? This could provide a very quantitative information for analysis the AFM (and maybe the Raman as well)

Response: We appreciate this valuable suggestion. We agree that simulating the electrolyte-graphite interface with applied potentials is complementary to the AFM and in situ Raman results and support their quantitative interpretation. In the revised manuscript, we have performed classical molecular dynamics simulations specifically modeling the interface between the electrolyte and a graphite model. These simulations provide detailed atomic-density profiles and solvation structures at the interface, offering the quantitative insight requested by the reviewer. Specifically, the simulation results reveal that the simulated density profiles clearly show the formation of distinct solvation layers near the carbon surface, which quantitatively matches the layer thickness and "steps" observed in the AFM force curves. The simulations identify the specific species populating the inner Helmholtz plane, providing direct structural evidence to support the spectral features observed in the Raman measurements. These new computational results bridge the gap between our experimental observations and the theoretical mechanism.

“To provide an atomistic resolution of the interfacial structure and corroborate the experimental inferences, we performed molecular dynamics simulations using the Constant Potential Method (CPM), as shown in Fig. 2a. The

simulation explicitly captures the potential-dependent restructuring of the electrical double layer (EDL) at the graphite-electrolyte interface. At potentials negative relative to the PZC, the simulated density profiles and snapshots reveal that the innermost Helmholtz plane is predominantly occupied by adsorbed free G4 solvent molecules rather than desolvated Li^+ ions. These interfacial G4 molecules preferentially adopt a flattened orientation parallel to the basal plane, forming a dense solvent layer that is consistent with the ~0.32 nm step thickness observed in AFM. The solvated $Li(G4)^+$ cations are statistically located in the second layer, separated from the electrode by this solvent cushion. Conversely, under positive polarization, the simulation shows the accumulation of FSI^- anions co-adsorbed with G4 molecules to screen the positively charged surface (Supplementary Fig. 19). These trends are in well agreement with the charge-dependent interfacial layering resolved by AFM." (Page 11, Line 282)

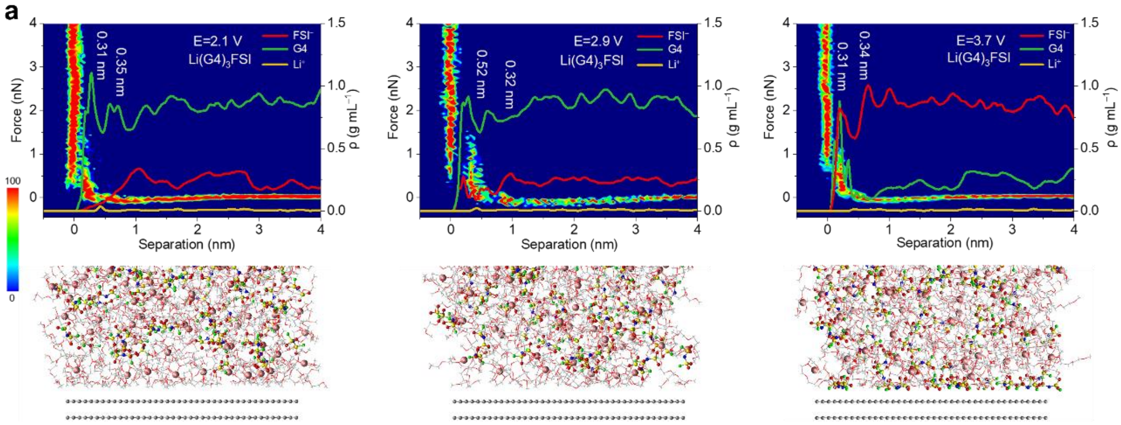

Figure R4–4, **a,** Two-dimensional AFM force-separation histograms from 20 independent force curves for $[Li(G4)_3][FSI]$ obtained at potentials of E=2.1 V_{Li}, E=2.9 V_{Li}, and E=3.7 V_{Li} with probability colour bar on the left-hand side and ion number density profiles of FSI^- (red), G4 (green) and Li^+ (yellow) from MD simulations at PZC−1.0 V, PZC, PZC+1.0 V (top row) . Representative snapshots from the simulation at −1.0 V, 0 V and +1.0 V relative to the PZC (bottom row), shown from left to right.

"In contrast to the dilute regime, where free solvent molecules dominate the interface (Fig. 3a and Supplementary Fig. 26), the simulation reveals that in highly-concentrated electrolytes the scarcity of free G4 molecules precludes the formation of a solvent-passivated layer. Instead, the interface exhibits a distinct alternating ion-layering mechanism. At potentials negative of the PZC, the innermost Helmholtz plane is populated primarily by bulky $Li(G4)^+$ cations, corresponding to the larger step thickness (~0.52 nm) detected by AFM. Conversely, under positive polarization, the interface becomes saturated by a compact layer of FSI^- anions, aligning perfectly with the reduced step thickness (~0.32 nm)." (Page 14, Line 363)

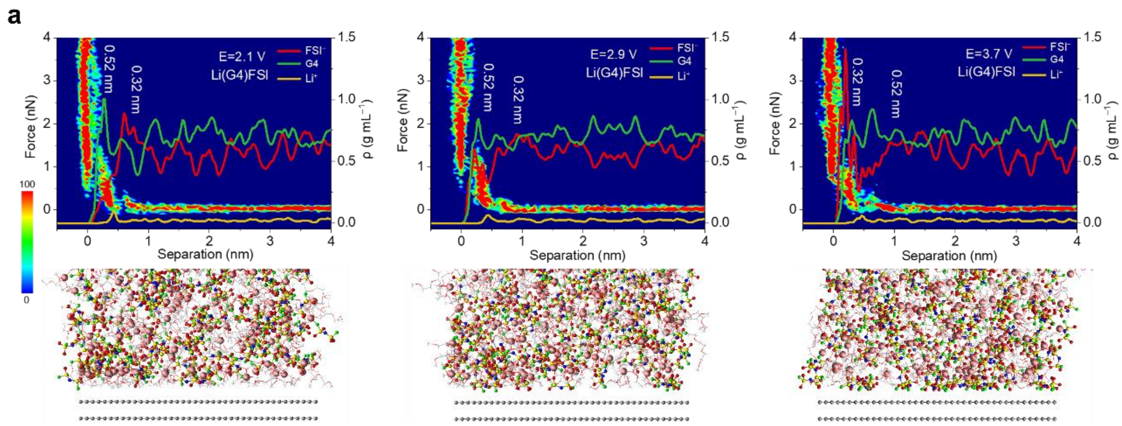

Figure R4–5**, a,** Two-dimensional AFM force-separation histograms from 20 independent force curves for [Li(G4)][FSI] obtained at potentials of E=2.1 V_{Li}, E=2.9 V_{Li}, and E=3.7 V_{Li} with probability colour bar on the left-hand side and ion number density profiles of FSI^- (red), G4 (green) and Li^+ (yellow) from MD simulations at PZC−1.0 V, PZC, PZC+1.0 V (top row) . Representative snapshots from the simulation at −1.0 V, 0 V and +1.0 V relative to the PZC (bottom row), shown from left to right.

Comment 6: The claim of entropy being the "design lever for optimizing solvation dynamics and guiding next generation electrolyte development" is mostly based on an analysis of the radial distribution functions using a modified Kirkwood functional. However, when looking at ref. 56, the latter was only used for bcc iron, and there is no proof that it remains valid for ionic systems as we know the latter are remarkably difficult to represent using conventional liquid state theory (in particular at high concentrations, where they start to have ionic liquids-like properties)

Response: We sincerely apologize for the confusion caused by an erroneous citation in the original manuscript. We agree with the reviewer that the reference to the bcc iron study (Ref. 56) was contextually incorrect for our liquid system. The method we actually employed is the Two-Body Excess Entropy (S_2) approximation, a standard statistical mechanical approach derived from liquid state theory to quantify the entropy loss due to spatial correlations (i.e., solvation structure). The specific formula used is:

$$S_2 = -2\pi\rho\, k_B \int_0^{\infty} [g(r)\ln g(r) - g(r) + 1]\, r^2\, dr,$$

where g(r) is the radial distribution function, ρ is the number density, and k_B is the Boltzmann constant. Contrary to the limited scope of the erroneous reference, this S_2 approximation is widely established and rigorously applied in complex fluid systems, including highly charged ionic environments and binary mixtures. For instance, Rai et al. (Ind. Eng. Chem. Res. 2025, 64, 8565) successfully utilized this exact two-body excess entropy formulation to analyze the counterion-specific collapse of highly charged polyelectrolytes, demonstrating its validity in systems dominated by strong electrostatic interactions. Soni et al. (Journal of Molecular Liquids 2024, 394, 123785)

employed this method to evaluate the "rigidity" of solvation structures in mixed solvents, correlating the excess entropy with diffusivity and critical solution temperatures. In our work, this metric serves as a robust proxy to capture the structural "stiffness" of the Li^+ solvation shell. A more negative S_2 corresponds to a more structured (rigid) environment, while a less negative value indicates a more disordered (labile) shell, which aligns perfectly with our hypothesis of entropy-driven exchange.

"To quantify the concentration-dependent evolution of solvation entropy, we employed the two-body excess entropy (S_2) approximation derived from liquid-state statistical mechanics. This framework captures the entropy contribution arising from pairwise spatial correlations and has been widely used to characterize structural ordering in complex fluids and ionic systems[57,58]. Specifically, the solvation entropy associated with Li^+ was evaluated from the radial distribution functions between Li^+ and surrounding species, according to

$$S_2 = -2\pi\rho\, k_B \int_0^{\infty} [g(r)\ln g(r) - g(r) + 1]\, r^2\, dr,$$

where g(r) is the radial distribution function, ρ is the number density, and k_B is the Boltzmann constant.

Within this formalism, a more negative S_2 indicates a more structured (rigid) environment, while a less negative value indicates a more disordered (labile) sheath." (Page 17, Line 435)

Comment 7: In the methods section the authors mention calculating the conductivity and viscosity of the liquids, but the data is not provided. How does it compare with experiments?

Response: We sincerely thank the reviewer for raising this point. We apologize for the oversight in not presenting the calculated data in the original manuscript. Viscosity was not used in any analysis, interpretation, or conclusion of this study. Its mention in the Methods section was unintentional and merely reflected the general capabilities of our simulation framework. We have removed this statement in the revised manuscript to avoid any misunderstanding. We have now included ionic conductivity (σ) in Table S2 of the Supporting Information. Specifically, we calculated the σ from the self-diffusion coefficients (D) obtained in our simulations (Figure R4–6) using the Nernst-Einstein equation:

$$\sigma = \frac{N e^2}{V k_B T} (D_+ + D_-).$$

Our calculated ionic conductivity for the concentrated system (LiFSI: G4 = 1:1) is 1.53 mS cm^{-1}. This value shows excellent agreement with experimental reports for similar solvate ionic liquid systems. For instance, Ueno et al. (J. Phys. Chem. B 2012, 116, 11323−11331) reported an experimental conductivity of 1.4 mS cm^{-1} for the equimolar [Li(G4)][FSI] complex. The slight deviation is within the expected range for molecular dynamics simulations, confirming that our model accurately captures not only the static solvation structure but also the dynamic transport properties of the electrolyte.

“Supplementary Table 2. Ionic Conductivity for glyme-Li salt complexes at 30 °C” (SI, Page 34)

Glyme-Li salt mixture	$\sigma_{sim.}$ (mS cm^{-1})	$\sigma_{ref.}$ (mS cm^{-1})
Li(G4)FSI	1.53	1.4[5]
Li(G4)$_3$FSI	7.16	~3.5[6]

σ: ionic conductivity

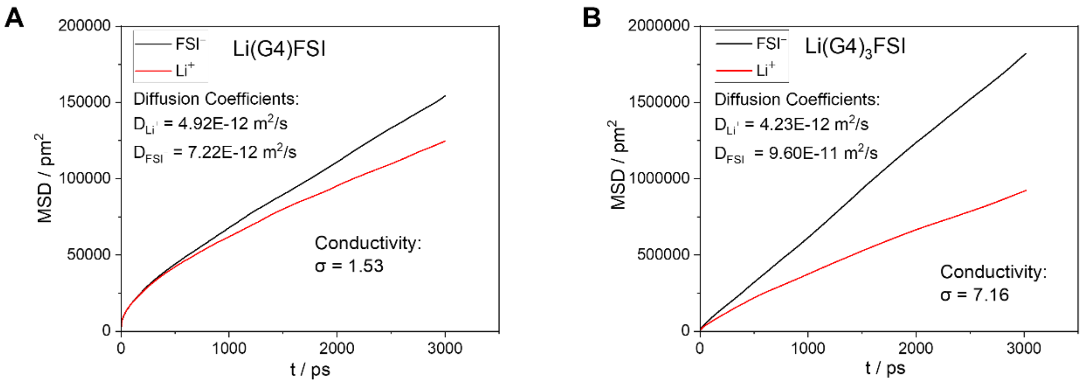

Figure R4–6, Ionic Conductivity for Li(G4)FSI (**A**) and Li(G4)$_3$FSI (**B**).

Comment 8: The authors point towards ref. 62 for the force field, but the latter does not give the ML model (maybe as it is not published yet). Please provide the model for the current system to ensure reproducibility of the results.

Response: Thank you very much for this comment. We have updated the reference to the recently published Nature Communications paper (https://www.nature.com/articles/s41467-025-67982-0) and provided the direct open-access repository link (https://doi.org/10.12463/AI4EC/QZCYP1) in the revised manuscript to ensure full reproducibility.

Point-by-point Response to Review Comments

Reviewer #1

I have read the rebuttal letter with change log, updated supplementary information, and the new version of the manuscript. The authors have made all necessary modifications to address my comments. I have no further observations.

Response: We thank the reviewer for the careful evaluation of our revised manuscript. We are pleased that the revisions have satisfactorily addressed all the concerns raised.

Reviewer #2

I am happy with the response and changes made by the authors. I am specially pleased with their response to Comment 5. I recommend publication.

Response: We thank the reviewer for the positive assessment and for recommending publication of our work. We are pleased that the revisions have satisfactorily addressed the concerns raised.

Reviewer #3

I have carefully reviewed the response letter and the revised manuscript. Regrettably, several critical concerns remain inadequately resolved, and I hold the opinion that this manuscript is not suitable for publication in Nature Communications. I suggest rejection of this work.

Response: We thank the reviewer for the careful evaluation of our manuscript. Below, we provide point-by-point responses to the reviewer's comments. Our simulation results show good agreement with experiments, and we have added more details and validation of the applied force field and simulation methods, which we hope could help address the reviewer's concerns.

Comment 1: Since the authors wanted to leverage the ab initio accuracy of MLMD, why don't they use MLMD to conduct simulations of the EDL? In terms of the constant-potential classical MD simulations of the EDL, I have big concerns about the methods reported in the revised manuscript. For example, the authors did not provide the details for the OPLS-AA force field, and it looks like the author did not apply any charge scale to the Li+ anions and anion species, which tend to overestimate the ion pairs (Phys. Chem. Chem. Phys., 2011,13, 2613-2626).

Response: We thank the reviewer for this important comment. In the revised manuscript, we have substantially rewritten the Methods section of molecular dynamics to provide a more rigorous and transparent description of the simulation strategy, force field construction, and validation procedure, thereby making the rationale and accuracy of our computational approach much clearer. With respect to the reviewer's suggestion that MLMD should be used to simulate the EDL, we agree that MLMD can, in principle, approach *ab initio* accuracy, but is not mature yet for the present system. A reliable machine learning potential (MLP) for this problem would need to explicitly account for electrode polarization, long-range electrostatic interactions, and the response to an external electric field, which is considerably more demanding than the training of conventional MLMD potentials for neutral bulk systems. More importantly, the interfacial processes studied here do not involve bond breaking or bond formation, but are dominated by ion solvation, ion pairing, and field-induced structural rearrangement. <u>Under these conditions, constant-potential molecular dynamics with classical force field is already well established as an appropriate method for capturing the essential electrostatic response and structural organization of the EDL.</u> Therefore, in the context of the present study, CMD provides a physically sound and computationally efficient description of

the interfacial behavior of the electrolyte.

We also appreciate the reviewer's concern regarding the force-field details and agree that these should be described more explicitly. In the revised manuscript, we now clarify that the parameters for G4 were generated using the LigParGen web server within the Optimized Potentials for Liquid Simulations All-Atom (OPLS-AA) framework. For LiFSI, the bonded and nonbonded parameters were taken from the ionic-liquid force field developed by Canongia Lopes and Pádua. However, the atomic partial charges of FSI anion were not directly adopted from the original parameter set. Instead, they were recalculated using the restrained electrostatic potential (RESP) scheme on the basis of Gaussian 16 calculations at the B3LYP/6-31+G(d) level with electrostatic potential fitting, and the charges of symmetry-equivalent atoms were averaged before being assigned in the simulation. This treatment was adopted because it reproduces the local Li^+-O_{FSI^-} coordination number obtained from MLMD much more faithfully for both low concentration and high concentration. **More importantly, it also reproduces the relative populations of aggregates (AGG), contact ion pairs (CIP), and solvent-separated ion pairs (SSIP) inferred from the Raman measurements.** We believe that this agreement with both MLMD results and experimental Raman speciation provides strong support for the reliability of the present parametrization scheme.

"The intermolecular interactions for the electrolyte species were described as follows: the parameters for G4 were generated using the LigParGen web server[68] within the OPLS-AA (Optimized Potentials for Liquid Simulations-All Atom) framework[69], whereas the bonded and nonbonded parameters for LiFSI were taken from the ionic-liquid force field developed by Canongia Lopes and Pádua[70]. The atomic partial charges of LiFSI were recalculated using the restrained electrostatic potential (RESP)[71] scheme based on Gaussian 16 calculations at the B3LYP/6-31+G(d) level[72-75] with electrostatic potential fitting, and the charges of symmetry-equivalent atoms were averaged before being assigned in the simulations." (Page 24, Line 618).

Following the reviewer's suggestion, we also tested a charge-scaling treatment by applying a uniform scaling factor of 0.8 to the original ionic charges. However, this procedure did not improve the description of the electrolyte structure in our system. On the contrary, the calculated Li–O coordination number between Li^+ and the oxygen atoms in FSI^- remained overestimated, and the resulting ion-speciation trend showed poorer agreement with the Raman-derived

AGG/CIP/SSIP distribution. Therefore, at least for the present electrolyte system, the unscaled RESP-derived charges provide a more accurate description of ion association and interfacial solvation than the uniformly scaled charges. These points have now been clarified in the revised manuscript.

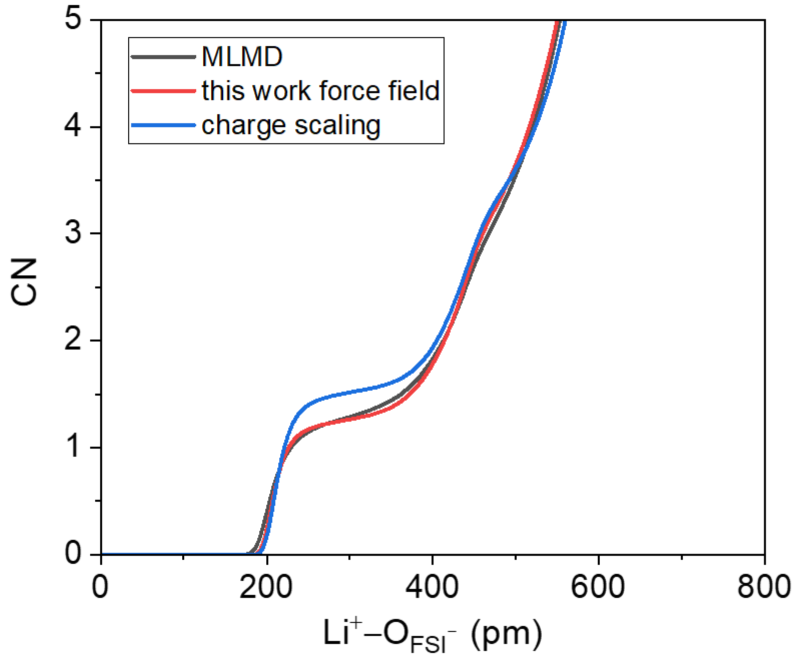

Figure R1-1. Radial distribution functions of Li–O pairs obtained from different force fields

Comment 2: How were SSIP, CIP, and AGG accounted for in the MD simulations of the EDL? The authors reported MD-simulated SSIP, CIP, and AGG in comparison with experimental data, what about the data under other voltage conditions?

Response: Thank you for this comment. In our molecular dynamics analysis of the electrical double layer, the fractions of SSIP, CIP, and AGG were determined by analyzing the local solvation structures of Li^+ ions located within 10 Å of the carbon electrode surface under each applied potential (Chemical Science, 2024, 15, 10908-10917). Specifically, for each voltage condition, we identified Li^+ ions in this interfacial region and classified their coordination environments according to the number of coordinated FSI^- anions. The definitions of SSIP, CIP, and AGG follow the criterion commonly adopted in the literature (J. Am. Chem. Soc. 2014, 136, 5039−5046): Li^+ species coordinated by no FSI^- anion was assigned as SSIP, those coordinated by one FSI^- anion were assigned as CIP, and those coordinated by two or more FSI^- anions were assigned as AGG. In this way, the reported SSIP/CIP/AGG fractions were extracted directly from the interfacial solvation structures sampled in the simulations.

In response to the reviewer's concern, we have further analyzed the SSIP, CIP, and AGG fractions under different applied voltages. The results show that the relative populations evolve systematically with potential, reflecting the voltage-dependent reorganization of the interfacial solvation environment in the electrical double layer. Importantly, the simulated trend under different voltage conditions remains in good agreement with the experimentally observed speciation evolution. We have now clarified the classification procedure in the revised manuscript and added the corresponding analysis at different voltages to make the comparison with experiment completer and more transparent.

"Across other applied potentials, despite quantitative deviations, the simulated evolution of SSIP and CIP follows the Raman trends, exhibiting clear potential dependence (Supplementary Fig. 20)" (Page 12, Line 307).

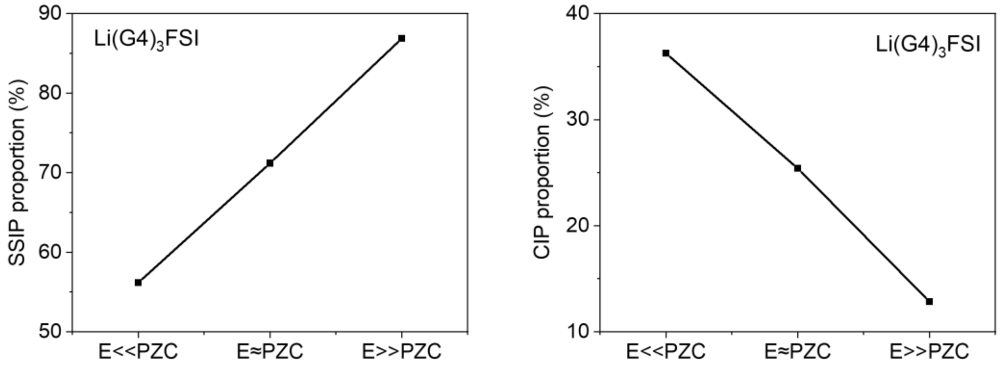

Figure R1-2. Potential-Dependent Evolution of Simulated SSIP and CIP Fractions in $Li(G4)_3FSI$.

"Their potential-dependent evolution is also consistent with the experimental trends (Supplementary Fig. 27)." (Page 14, Line 365).

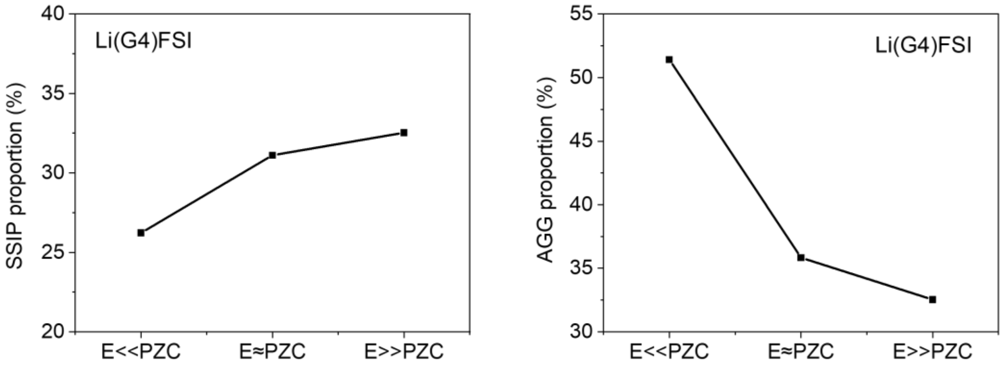

Figure R1-3. Potential-Dependent Evolution of Simulated SSIP and AGG Fractions in Li(G4)FSI.

We further employed a charge-scaling treatment to investigate the evolution of SSIP population as a function of applied potential in Li(G4)FSI and $Li(G4)_3FSI$. In the high-concentration system, the SSIP fraction remains essentially unchanged under negative potentials, whereas a sudden increase is observed upon positive polarization. In contrast, in the dilute system, the SSIP population is nearly insensitive to the applied potential. These results suggest that the charge-scaling treatment suppresses the electric-field-driven rearrangement of interfacial solvation structures, leading to a reduced response of the system to the applied potential. The corresponding trends are shown in Figure R1-4.

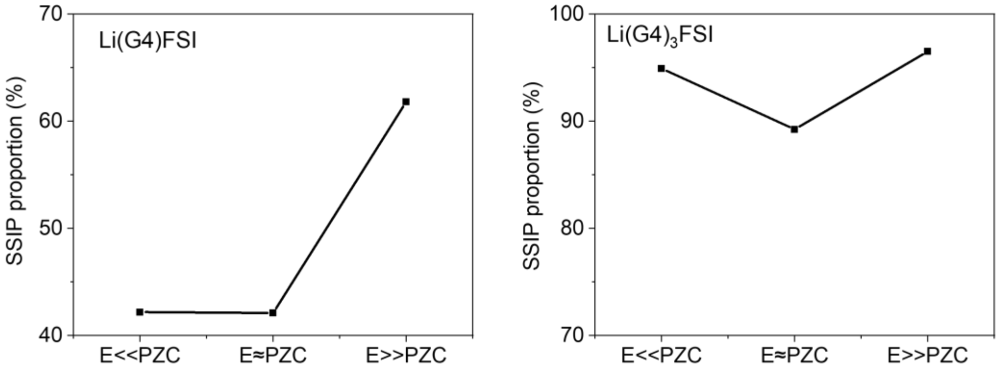

Figure R1-4. SSIP fraction vs. potential in Li(G4)FSI and $Li(G4)_3FSI$ under charge scaling treatment.

Comment 3: In the reply, the authors claimed "The new interfacial simulation results clearly demonstrate that the trend observed in the bulk is preserved and even amplified within the EDL. Under polarization, the exchange frequency between Li+ and anions in the high-concentration system is significantly higher than that in the dilute system. The CPM results confirm that the "looser" solvation sheath identified by MLMD facilitates easier desolvation and reorganization at the interface, directly supporting the mechanism of enhanced reversibility." I do not see data that supports those statements.

Response: Thank you for this comment. We recognize that our previous response was not sufficiently clear, and we have now revised it for better clarity. We first evaluated the accuracy of the MLMD force field by calculating the ionic conductivity using the Einstein–Helfand formalism, and the simulated values are in good agreement with experimental measurements, confirming the reliability of the MLMD description. To ensure consistency between different simulation frameworks, the CPM force field was carefully tuned to closely reproduce the MLMD interactions (Figure R1–5a). Using this optimized CPM model, we further investigated the interfacial speciation. Specifically, the

solvation structures obtained from MLMD bulk simulations were compared with the interfacial compositions from CPM simulations at 0 V. As shown in Figure R1–5b, the two approaches yield highly consistent results, supporting the validity of the CPM model for describing interfacial behavior. Moreover, the interfacial simulation results indicate that the solvation trend observed in the bulk is also present within the EDL under polarization. In combination with the experimental results, this is consistent with interfacial solvation environment in the high-concentration electrolyte (Figure R1–5c). With increasing potential, the interfacial solvation structure undergoes continuous reorganization, while anions persistently participate in the primary solvation sheath. Such an anion-involved solvation environment is beneficial for the reversible intercalation and deintercalation of Li^+. As a result, the system maintains a high reversible capacity of 320 mAh g^{-1} after 100 cycles without inducing exfoliation of the graphite anode (Figure R1–5d). A similar perspective has been reported in previous work by Fan et al., where the coupling between bulk solvation dynamics and battery performance was systematically established (Nature, 2024, 627, 101–107).

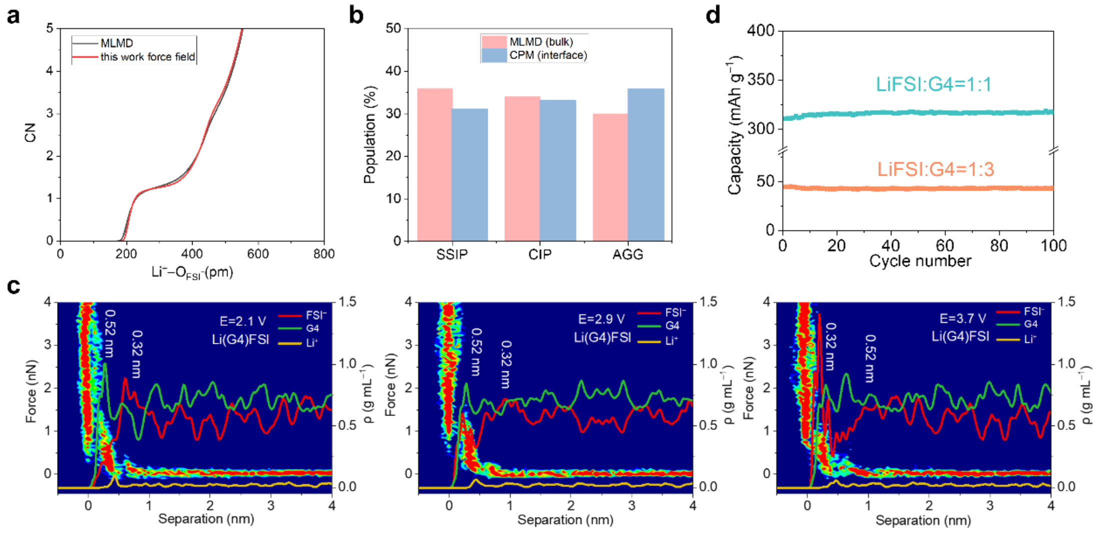

Figure R1-5. **a**, Comparison of different force fields. **b**, Comparison of bulk solvation structures obtained from MLMD simulations and interfacial solvation structures from CPM simulations. **c**, Two-dimensional AFM force-separation histograms from 20 independent force curves for Li(G4)FSI obtained at potentials of E=2.1 V_{Li}, E=2.9 V_{Li}, and E=3.7 V_{Li} and ion number density profiles of FSI^- (red), G4 (green) and Li^+ (yellow) from MD simulations at PZC−1.0 V, PZC, PZC+1.0 V. **d**, Cycle performance of Li(G4)FSI and $Li(G4)_3FSI$.

Comment 4: The authors used the Nernst-Einstein equation to calculate the ionic conductivity. Despite they claim the calculated ionic conductivity is close to the experimental value, it is widely known that the Nernst-Einstein equation does not consider ion correlation and thus does not apply to the highly-concentrated electrolytes at all (ACS Cent. Sci. 2019, 5, 7, 1250–1260).

Response: Thank you for this comment. We agree that the Nernst–Einstein equation neglects ion–ion correlations and therefore is not appropriate for quantitatively evaluating ionic conductivity in highly concentrated electrolytes. Our previous description was therefore not sufficiently rigorous. Following the reviewer's suggestion, we have recalculated the ionic conductivity using the Green–Kubo and Einstein–Helfand formalisms, which explicitly account for correlated ionic motion and are more suitable for concentrated electrolyte systems. The newly calculated conductivity values have been added to the Supporting Information in tabulated form. The conductivity values obtained from these correlation-based approaches remain in agreement with the experimental results. We have accordingly revised the relevant discussion in the manuscript to remove the potentially misleading use of the Nernst–Einstein equation and to clarify that the reported conductivity analysis is now based on the more appropriate Green–Kubo and Einstein–Helfand treatments.

"Nevertheless, the overall trend and the physical picture of the solvation structure remain consistent between the simulation and experiment. Moreover, the ionic conductivity was recalculated using the Einstein–Helfand formalism[81], which explicitly account for correlated ionic motion. The resulting conductivity values remain in agreement with the experimental measurements, confirming that the simulations capture the essential macroscopic transport behavior and provide a reliable basis for qualitative analysis." (Page 26, Line 658).

Glyme-Li salt mixture	$\sigma_{sim.}$ (mS cm^{-1})	$\sigma_{sim*.}$ (mS cm^{-1})	$\sigma_{ref.}$ (mS cm^{-1})
Li(G4)FSI	1.53	2.20	1.4[5]
$Li(G4)_3FSI$	7.16	9.89	~3.5[6]

σ: ionic conductivity. σ_{sim*} are calculated from the Einstein–Helfand formalism, whose trend is reasonable, while the remaining deviation in absolute value is likely due to the limited 10 ns MLMD trajectory length". (SI, Page 38).

Reviewer #4

I think that the additional experiments/simulations and corrections made by the authors have clearly improved the manuscript, that can now be published in Nature Communications. I just have a few comments that remain to be addressed:

Response: We sincerely thank the reviewer for the positive and encouraging evaluation of our revised manuscript. Below, we provide point-by-point responses to the remaining comments and have further revised the manuscript accordingly to address these concerns.

Comment 1: Regarding the response to my comment #1, EIS fit: The authors did not answer my point. They show that their fit is good by comparing with another equivalent circuit, which I never asked. My question was how sensitive was the fit with respect to the parameters variations, keeping the initial equivalent circuit. This can be done by looking at the covariance matrix, or even better by performing a global sensitivity analysis. In particular I would be interested in seeing how sensitive is the fit to the various contributions to the capacitance.

Response: We thank the reviewer for the clarification and sincerely apologize for not adequately addressing the sensitivity of the EIS fitting in our previous response. We have now analyzed the robustness of the fitting based on the initial equivalent circuit, taking the Li(G4)FSI system as a representative example.

The results show that the fitting exhibits good reproducibility and low fitting errors, indicating that the parameters are well-defined and that each parameter contributes meaningfully to the model. In contrast, overparameterization would typically lead to larger fitting errors. Analysis of the covariance matrix (in particular, the correlation matrix) reveals two strongly correlated parameter clusters. Specifically, parameters 1–3 (red box) show rather correlation and correspond to the high-frequency semicircle, while parameters 4–6 (blue box) are correlated and correspond to the intermediate-frequency semicircle. Parameter 7 shows negligible correlation with the others, indicating that it behaves as an independent variable and corresponds to the low-frequency semicircle, as shown in Figure R2-1. Overall, these results confirm the robustness of the fitting, and the conclusions derived from the EIS analysis remain unchanged.

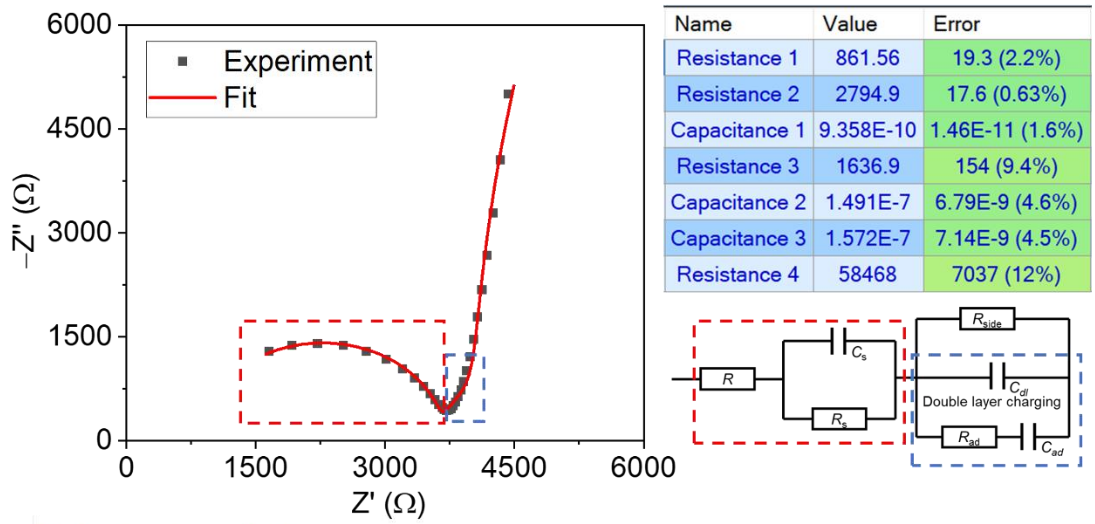

Name	Value	Error
Resistance 1	861.56	19.3 (2.2%)
Resistance 2	2794.9	17.6 (0.63%)
Capacitance 1	9.358E-10	1.46E-11 (1.6%)
Resistance 3	1636.9	154 (9.4%)
Capacitance 2	1.491E-7	6.79E-9 (4.6%)
Capacitance 3	1.572E-7	7.14E-9 (4.5%)
Resistance 4	58468	7037 (12%)

Covariance matrix:

1	70.40732787	-59.5730001	1.2457E-010	139.1964753	-8.863E-009	9.2941E-009	8353.720957
2	-59.5730001	57.62042108	-1.044E-010	-11.3699743	9.7456E-010	-9.638E-010	-1466.71113
3	1.2457E-010	-1.044E-010	2.5443E-022	2.8351E-010	-1.781E-020	1.8776E-020	1.4640E-008
4	139.1964753	-11.3699743	2.8351E-010	6774.508528	-2.766E-007	3.1856E-007	3.7047E+005
5	-8.863E-009	9.7456E-010	-1.781E-020	-2.766E-007	1.6387E-017	-1.654E-017	1.8716E-006
6	9.2941E-009	-9.638E-010	1.8776E-020	3.1856E-007	-1.654E-017	1.8234E-017	6.4230E-006
7	8353.720957	-1466.71113	1.4640E-008	3.7047E+005	1.8716E-006	6.4230E-006	1.9768E+008

Correlation matrix:

1	1.000000000	-0.93530300	0.930694407	0.201548890	-0.26092334	0.259393036	0.070809512
2	-0.93530300	1.000000000	-0.86231281	-0.01819837	0.031715156	-0.02973567	-0.01374285
3	0.930694407	-0.86231281	1.000000000	0.215943669	-0.27583603	0.275656114	0.065278423
4	0.201548890	-0.01819837	0.215943669	1.000000000	-0.83026166	0.906385195	0.320134787
5	-0.26092334	0.031715156	-0.27583603	-0.83026166	1.000000000	-0.95708613	0.032883353
6	0.259393036	-0.02973567	0.275656114	0.906385195	-0.95708613	1.000000000	0.106983699
7	0.070809512	-0.01374285	0.065278423	0.320134787	0.032883353	0.106983699	1.000000000

Figure R2-1. Equivalent circuit fitting and error analysis.

Comment 2: Regarding the response to my comment #4: The authors mention: "To address this limitation and provide a rigorous calculation of the PZC, we have incorporated Classical Molecular Dynamics simulations using the Constant Potential Method (CPM) in the revised manuscript. The results presented in Figure 3g (in the revised version) are now explicitly derived from these new CPM simulations, which allow us to simulate the open-circuit conditions and determine the electrode potential at zero surface charge density". However, the constant potential method corresponds to a fixed potential and not to open-circuit conditions. Can the authors update the method description accordingly?

Response: We thank the reviewer for pointing this out. We agree that our previous wording was not accurate. The CPM describes the interfacial structure under fixed applied potentials, rather than open-circuit conditions. In our simulation setup, 0 V was taken as the reference potential. We have therefore

revised the corresponding description in the Methods section to avoid confusion and to clarify the meaning of the CPM simulations.

"The system was first equilibrated for 10 ns at 0 V, which was taken as the reference potential in our CPM setup, to relax the interfacial structure. Following equilibration, production runs were performed for 10 ns under various constant potential conditions." (Page 25, Line 640).

Comment 3: In the methods section, reference 70 (doi:10.1103/PhysRevLett.110.196801) is quoted for the CPM calculations, but the corresponding article is not using/implementing this method. Please correct.

Response: We thank the reviewer for pointing this out. We agree that the citation given in the previous Methods section was incorrect, and we apologize for this oversight. In the revised manuscript, we have corrected the citation and updated the Methods section accordingly (J. Chem. Phys. 2007, 126, 084704; J. Chem. Phys. 1995, 102, 511-524). Specifically, the CPM methodology is now referenced to the appropriate literature describing constant-potential molecular dynamics simulations of electrochemical interfaces. Where relevant, we have also cited the corresponding implementation reference used in our simulations. The text in the Methods section has been revised accordingly to ensure that the methodological description and the cited references are fully consistent.

Point-by-point Response to Review Comments

Reviewer #3

I appreciate the authors' efforts to reply to the questions, but I still have big concerns about the simulation part. The reliability of the simulation data is highly doubted. I won't suggest publication of this work before these concerns are thoroughly addressed.

Response: We appreciate the reviewer's continued evaluation of our work. We recognize that our previous response was not as clear as it should have been, which may have caused confusion regarding the simulation methodology and data interpretation. To address these concerns, we have now added further benchmark calculations. These new results provide a clearer comparison among the different force-field treatments and further support the reliability of the simulation used in this work.

Importantly, we do not intend to use the simulations as standalone proof of the interfacial mechanism. Rather, the simulations are employed to provide molecular-level insights that complement the experimental observations. The key trends obtained from the simulations are consistent with the experimental results. This agreement supports the reliability of the proposed interpretation.

Comment 1: The authors tested a charge-scaling treatment by applying a uniform scaling factor of 0.8 to the original ionic charges, but found larger Li–O coordination number between Li^+ and the oxygen atoms in FSI^- compared to the case without charge scaling. This is awkward. A charge scaling of 0.8 must weaken the intertactions between Li^+ and FSI^-.

Response: We thank the reviewer for this important comment. We fully agree that, in principle, applying a uniform charge-scaling factor of 0.8 should weaken the Coulombic interactions between Li^+ and FSI^- relative to the unscaled force field. We apologize that our previous response did not explain this comparison clearly enough and may have caused confusion.

To clarify this point, we have now performed additional CMD simulations using three different force-field treatments: (i) the original ionic-liquid force field developed by Canongia Lopes and Pádua (denoted as ILFF), (ii) the uniformly

charge-scaled ILFF model with a scaling factor of 0.8, and (iii) the RESP-refitted force field adopted in the present manuscript. The resulting Li–O_{FSI} coordination structures were then directly compared with the MLMD benchmark. The revised results show a clear and internally consistent trend. As expected, the original ILFF gives the largest Li–O_{FSI} coordination number, indicating significant overestimation of Li^+–FSI^- association strength (shown in Figure R1-1). After applying the 0.8 charge scaling, the Li–O_{FSI} coordination number decreases noticeably, confirming that charge scaling indeed weakens the Li^+–FSI^- interaction.

However, although the 0.8-scaled ILFF partially alleviates the overbinding tendency, its Li–O_{FSI} coordination number remains higher than the MLMD reference result. In contrast, the RESP-refitted force field yields much closer agreement with MLMD. This difference arises because the RESP-refitted model not only reduce the overall charge magnitude, but also redistributes the local electrostatic environment through reparameterized atomic charges derived from DFT-based electrostatic potential fitting. (Polymers 2021, 13, 1131; J. Phys. Chem. C 2013, 117, 3747−3761; J. Chem. Theory Comput. 2021, 17, 4274−4290). Consequently, the RESP-refitted force field provides a more balanced description of Li^+–FSI^- coordination and solvation structure than the uniformly scaled ILFF model. Accordingly, the key conclusion is not that charge scaling strengthens Li^+–FSI^- interactions, but rather that uniform charge scaling only partially corrects the overbinding behavior of the original ILFF, whereas the RESP-refitted force field more accurately reproduces the MLMD benchmark solvation structure. Furthermore, for RESP-refitted force field, applying additional uniform charge scaling could over-attenuate the Li^+–FSI^- interaction, leading to excessive salt dissociation and consequently underestimate Li–O_{FSI} coordination numbers relative to the MLMD benchmark.

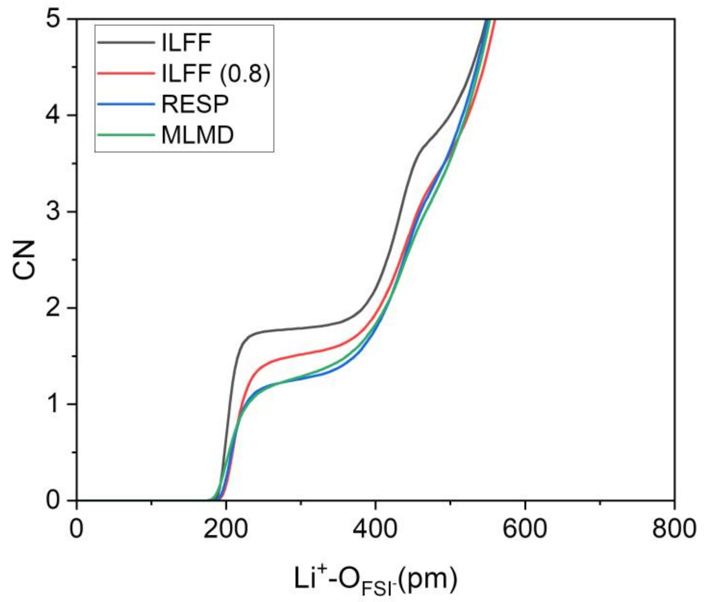

Figure R1-1. Radial distribution functions of Li–O_{FSI^-} pairs obtained from different force fields.

Comment 2: Why is the ionic conductivity calculated from the Einstein–Helfand formalism (2.2 mS/cm) even higher than that from Nernst-Eistein equation? This does not make sense. The ionic conductivity must drop, sometimes significantly, when ion correlation is considered, particularly for high concentration electrolytes (ChemElectroChem, 2020, 7 , 367 —385).

Response: We sincerely thank the reviewer for pointing out this important point regarding the conductivity analysis. In the previous revision, following the reviewer's valuable suggestion concerning ion-correlation effects in concentrated electrolytes, we additionally evaluated the ionic conductivity using the Einstein–Helfand (EH) formalism, which explicitly accounts for correlated ion motions beyond the Nernst–Einstein (NE) approximation.

We fully agree that, from a physical standpoint, the ionic conductivity obtained from the EH formalism should generally be lower than, or at most comparable to, that derived from the NE equation, because ion-ion cross-correlations in concentrated electrolytes typically reduce the net charge transport (ChemElectroChem, 2020, 7, 367–385). Therefore, the unexpectedly higher conductivity obtained from the EH formalism in the original manuscript does not indicate physically enhanced correlated ion transport, but rather reflects the

insufficient statistical convergence of the collective conductivity calculation within the accessible MLMD simulation timescale (~10 ns).

Specifically, the NE conductivity is calculated from the self-diffusion coefficients of individual ions and therefore benefits from extensive statistical averaging over many independent particle trajectories, leading to relatively rapid convergence. In contrast, the EH formalism evaluates the collective charge transport from the total charge displacement (or collective charge flux) of the entire simulation box. As a result, the EH conductivity is substantially more sensitive to long-timescale fluctuations and cancellation effects arising from correlated ion motions, particularly in highly concentrated electrolytes where strong ion pairing and clustering significantly slow the convergence of the distinct correlation contributions (Brehm, M. et al. ChemRxiv 2021, DOI:10.26434/chemrxiv.15002026/v3). Under such insufficient sampling conditions, the negative ion-correlation terms may not yet be fully converged, which can temporarily lead to nonphysical deviations such as $\sigma_{EH} > \sigma_{NE}$. Similar convergence difficulties for conductivity calculations based on collective correlation formalisms have also been widely discussed in previous methodological studies on concentrated electrolytes and ionic liquids (J. Chem. Theory Comput. 2023, 19, 5380−5393; J. Chem. Inf. Model. 2025, 65, 3477−3487).

We further note that the slow convergence of EH conductivity is a well-known methodological challenge for collective transport properties and is fundamentally different from the convergence behavior of structural or single-particle dynamical properties. In the present work, the MLMD simulations were primarily designed to validate the capability of the developed machine learning potential in reproducing local solvation structures and fundamental single-particle dynamics of the electrolyte. Achieving fully converged collective conductivity values would require substantially longer simulation trajectories than those employed in the present MLMD benchmarking study. Given that the computed conductivity already serves as good descriptors to confirm that our MLP can reproduce the underlying dynamics, we have revised the discussion Supporting Information to explicitly clarify these convergence limitations over the 10 ns timescale.

Page 38 in SI:

Supplementary Table 2. Ionic Conductivity for glyme-Li salt complexes at 30 °C

Glyme-Li salt mixture	σ_{sim*} (mS cm^{-1})	$\sigma_{ref.}$ (mS cm^{-1})
Li(G4)FSI	2.20	1.4[5]
Li(G4)$_3$FSI	9.89	~3.5[6]

σ: ionic conductivity. σ_{sim*} are calculated from the Einstein–Helfand formalism[7], whose trend is reasonable, while the remaining deviation in absolute value is likely due to the limited 10 ns MLMD trajectory length. The Einstein–Helfand relation tracks the net charge flux of the entire simulation box as a single collective particle, which suffers from severe statistical fluctuations and typically requires much longer trajectories to fully average out the noise and achieve high quantitative precision. Therefore, the σ_{sim*} values presented here are intended for qualitative reference and trend comparison.

Reviewer #4

The authors have well addressed my comments. I recommend publication in Nature Communications.

Response: We sincerely thank the reviewer for the positive evaluation of our work and for the constructive comments and suggestions throughout the review process. We greatly appreciate the reviewer's recommendation for publication in Nature Communications.

Point-by-point Response to Review Comments

Reviewer #3

I appreciate the authors' efforts to address these concerns. These simulation details should be added to the Supplementary Information, which should have been done at the very beginning, so that the simulation results can be reproduced. After that, I would suggest publication of this work. The next round review is necessary.

Response: We sincerely thank the reviewer for the positive assessment of our revisions and for recognizing our efforts to address the previous concerns.

Following the reviewer's suggestion, we have now incorporated the relevant simulation details into the Methods and Supplementary Information to ensure full transparency and reproducibility of the computational results. We believe that these additions provide sufficient information for reproducing the reported simulation results. The corresponding revisions have been highlighted in the Methods and Supplementary Information.

We thank the reviewer again for the constructive comments and recommendation for publication.

Page 21 in Methods:

simulations. As shown in Figure S32, we further performed CMD simulations for Li(G4)FSI electrolytes using the original ILFF, the 0.8 charge-scaled ILFF model, and

the RESP-refitted force field used in this work and the Li^+–O_{FSI}^- coordination numbers were compared with the MLMD benchmark. The results show a consistent trend ILFF overestimates Li^+–O_{FSI}^- coordination charge scaling reduces the coordination as expected but still does not reach the MLMD result while the RESP-refitted model provides the best agreement with MLMD. Therefore, the RESP-refitted force field was therefore used in the subsequent EDL simulations. A critical feature of this study is the

Page 37 in SI:

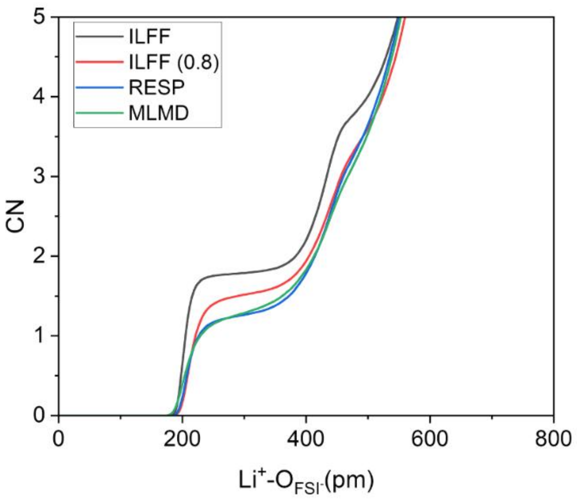

Supplementary Fig. 32. Coordination number (CN) of Li^+–O_{FSI^-} pairs in Li(G4)FSI electrolyte obtained from CMD simulations with different force fields.
